# Effect of Heterocyclic Ring on Ln^III^ Coordination, Luminescence and Extraction of Diamides of 2,2′-Bipyridyl-6,6′-Dicarboxylic Acid

**DOI:** 10.3390/molecules25010062

**Published:** 2019-12-23

**Authors:** Nataliya E. Borisova, Alexey V. Ivanov, Anastasia V. Kharcheva, Tsagana B. Sumyanova, Uliana V. Surkova, Petr I. Matveev, Svetlana V. Patsaeva

**Affiliations:** 1 Chemistry Department, M. V. Lomonosov Moscow State University, Moscow 119991, Russia; phthaliv@gmail.com (A.V.I.); harcheva.anastasiya@physics.msu.ru (A.V.K.); ts.sumyanova90@gmail.com (T.B.S.); uvsurkova@gmail.com (U.V.S.); petr.i.matveev@gmail.com (P.I.M.); 2 Department of Physics, M. V. Lomonosov Moscow State University, Moscow 119991, Russia; spatsaeva@mail.ru

**Keywords:** lanthanides, complexes, X-Ray structure, bipyridine, luminescence, stability constants

## Abstract

We have synthesized and examined several complexes of lanthanides with diamides of 2,2′-bipyridyl-6,6′-dicarboxylic acid bearing various hetaryl-based side chains for the elucidation of the effect of the heterocycle on the structure and properties of the ligands. The multigram scale methods for the preparation of various *N*-alkyl-hetaryls and their diamides were developed. The solid state structure of 6-methyl-2-pyridylamide of 2,2′-bipyridyl-6,6′-dicarboxylic acid possesses a flat structure where the conformation is completely different from that previously observed for *N*-alkylated 2,2′-bipyridyl-6,6′-dicarboxamides and 2,6-pyridinedicarboxamides. The complexes of new ligands were synthesized and NMR and X-Ray studied their structure in solution and solid state. The results demonstrate that complexes possess the same structures both in solid state and in solution. Stability constants of the complexes were less when comparing with dimethyl-substituted diamides, but higher than for unsubstituted dianilide. Contrarily, the extraction ability of 2-pyridyl-diamide is significantly lower than for corresponding anilide. Specific interaction of extractant with solvent molecules, which is not available for electron-sink pyridine amides, can explain this. The luminescence of new Eu complexes was significantly higher than for all previously 2,2′-bipyridyl-6,6′-dicarboxamides and QY reaches 18%. Asymmetry ratios of Eu complexes were 25% higher when compared other complexes with 2,2′-bipyridyl-6,6′-dicarboxamides, which indicates large deviation from the inversion center.

## 1. Introduction

Metal complexes with 2,2′-bipyridyl based ligands are widely used as fluorescent materials, in the creation of solar cells, as well as analytical reagents [1,2,3]. Complexes of corresponding compounds and rare earth elements (REEs) can be used as luminescent materials for creating lasers, waveguide amplifiers, photomultipliers [4], organic light-emitted diodes [5], radiation detectors of various nature [6,7], and 5f-metals separation extractants [8,9]. The optical properties of the complexes can be tuned finely with changes of the structure nearest environment of metal due to the ′antenna effect′ [10]. The metal separation efficiency also strongly depends on the substitution on the extractant molecule [9,11]. One of the promising classes of compounds for both f-metal separation and luminescent devices is derivatives of 2,2′-bipyridyl-6,6′-dicarboxylic acid (BPDA), which are tetradentate ligands of *N*,*N*′,*O*,*O*′-type. Previously, it has been shown that tertiary amides of 2,2′-bipyridyl-6,6′-dicarboxylic acid bearing substituted *N*-ethylanilines are potential materials for Am(III) separation [11,12]. Such ligands effectively separate the ions of 4f- and 5f-elements—a crucial step of modern closed (waste-free) nuclear fuel cycle. Moreover, their complexes with REE ions are of interest as potential luminescent materials [13], where the properties are also dependent on the substitution on the amidic moiety of the molecule [12]. Although the synthesis of substituted amides of 2,2′-bipyridyl-6,6′-dicarboxylic acid is already represented [12,13], the preparation of diamides of 2,2′-bipyridyl-6,6′-dicarboxylic acid that is based on heterocyclic amines has not been described in the literature. In light of the above, it is interesting to compare the properties of diamides that are based on anilines and aminopyridines as well as photophysical properties of their complexes with lanthanides metals and metal extraction. We expected that pyridine-containing amides by their characteristics should be similar to ligands that are based on anilines with electron acceptor groups, namely nitro-group [11]. In addition, these compounds and their complexes with metals can serve as convenient blocks in the synthesis of coordination polymers due to the coordination of the metal ion at the additional donor center—the nitrogen atom of the pyridine ring of the amide group.

## 2. Results and Discussion

### 2.1. Synthesis

#### 2.1.1. Preparation and Structure of Diamide-Based Ligands

**Scheme 1:**
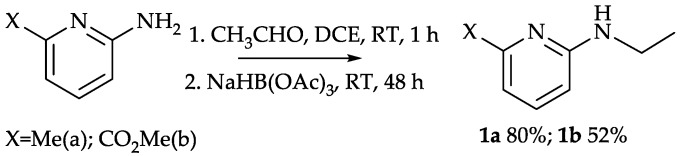
Reductive amination of 6-substituted 2-aminopyridines with acetaldehyde.

The effective methods for preparation of *N*-ethylaminopyridines is needed to retrieve the target diamide, 6-Methyl-2-*N*-ethylaminopyridine and 6-methylcarboxy-2-*N*-ethyl-aminopyridine were obtained by reductive amination of acetaldehyde with corresponding aminopyridine (Scheme 1). 

2-, 3- and 4-*N*-ethylaminopyridines cannot be obtained by this method due to partial reduction of the pyridine cycle. It should also be noted that the *N*-ethylation of aminopyridines by acetonitrile in presence of palladium [14] or by acetic acid in the presence of sodium borohydride [15] given in the literature do not lead to the production of ethylamine derivatives in preparative quantities.

**Scheme 2:**
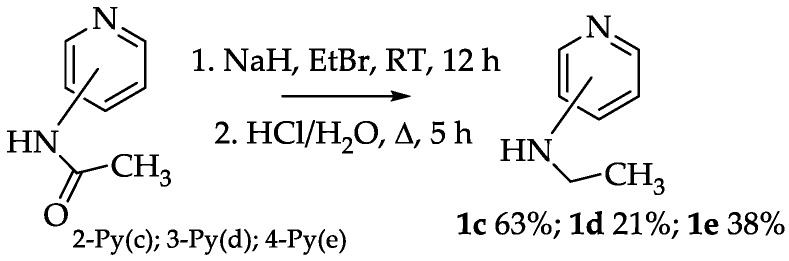
Ethylation and hydrolysis of *N*-acetylaminopyridines.

Alkylation of the corresponding *N*-acetylaminopyridines by ethylbromide in the presence of sodium hydride with subsequent hydrolysis of the acetyl group synthesized the required compounds (Scheme 2). The tert-butoxycarbonyl can be utilized instead of acetyl group, which allows for increasing the yield of *N*-ethylaminopyridines up to 70–90%.

In addition, we found that the preparation of 4-ethylaminopyridine is more convenient if the nucleophilic substitution of halogen in 4-chloropyridine with an excess of ethylamine at 115 °C in autoclave is utilized.

**Scheme 3:**
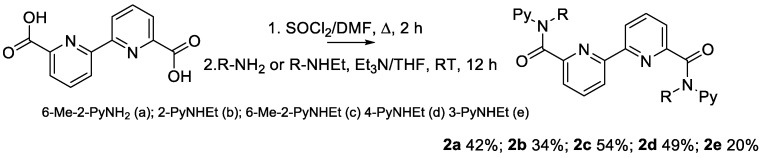
Synthesis of target diamides.

The preparation of the target diamides were made by acylation of corresponding *N*-aminopyridines by 2,2′-bipyridyl-6,6′-dicarboxylic acid dichloroanhydride generated in situ (Scheme 3). After preliminary study of the primary 6-methyl-2-aminopyridine acylaion, the corresponding procedure were expanded on the preparation of secondary amides to obtain the *N*,*N*′-diethyldiamides.

The yields of target compounds are somewhat lower than when using the *N*-ethylaniline due to the acceptor effect of the pyridine ring [11,12]. It is interesting to note the most acceptor 6-methylcarboxy-2-ethylaminopyridine cannot be acylated under these conditions at all.

^1^H- and ^13^C NMR spectroscopy and mass-spectrometry (MALDI-TOF) confirmed the structures of the synthesized compounds. The assignment of signals in the ^1^H-NMR spectra were carried out on the basis of 2D COSY experiments (Table 1 and Figures S1–S8 at ESI). Thus, two groups of cross-peaks are observed in the COSY spectrum of ligand **2c** in the region of aromatic protons: one between protons with chemical shifts 7.35−6.85 and 7.35−6.70 ppm, and the second, which includes signals at 7.70−7.80 and 7.70−7.57 ppm. These two groups of signals (spin systems AMX) belong to protons of two different pyridine rings. The determination of the belonging of these signals to the fragments of the bipyridyl or 6-methylpyridyl system can be carried out by analysis of the 2D ROESY spectrum. The cross-peaks between the 6-methyl group of the aminopyridine ring and proton at 6.64 ppm were only observed. The latter belongs to the proton in 5-position of the aminopyridine fragment and the whole group of signals of aromatic protons in the region 6.7−7.35 ppm attributed to the amine fragment of the ligand. The signals of protons of the rest of the ligands were assigned by the same way.

**Figure 1:**
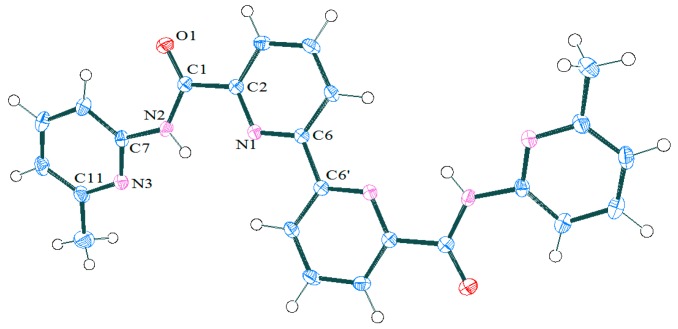
Molecular structure of **2a** showing the atom numbering scheme. Displacements ellipsoids are drawn at the 50% probability level and H atoms are shown as small spheres arbitrary radius.

X-ray determined the structure of the secondary diamide **2a** (Figure 1, Table 2 and Tables S1–S4 at ESI). The molecule possesses the center of symmetry at the middle of C6-C6′ bond of bipyridyl moiety. Aminopyridine rings are cis relative the oxygen atom of amidic group. The latter is fundamental difference of represented structure from *N*-alkylanilides of 2,2′-bipyridyl-6,6′-dicarboxylic acid [11,13] and *N*-alkylanilides of 2,6-pyridinedicarboxylic acid [16,17]. Contrary to the tertiary amides of 2,2′-bipyridyl-6,6′-dicarboxylic acid, the molecule of **2a** is almost flat: the torsion angle N1-C6-C6′-N1′ is 180.0(1)°. The angle between pyridine rings of bypyridyl moiety and aminopyridine groups is 23.19°. Torsion angle N1-C2-C1-N2 is 3.4(2)°, which evidenced the better conjugation between bipyridyl moiety and amidic group and aminopyridine aromatic ring when comparing with *N*-ethyl-derivatives [16,17].

#### 2.1.2. Synthesis and Structures of Ln^III^ Complexes

**Scheme 4:**
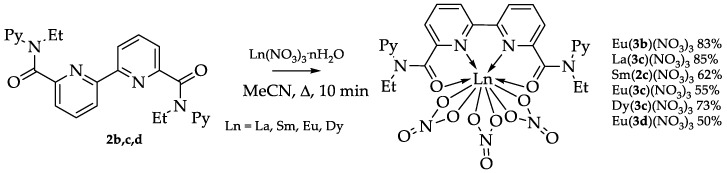
Preparation of Ln^III^ complexes.

The complexes with nitrates of lanthanum, samarium, europium, and dysprosium were synthesized by interaction of the ligands with nitrates of corresponding metals in acetonitrile (Scheme 4).

Standard spectroscopic techniques characterized the complexes. MADI-TOF mass spectra of the complexes show only one characteristic [LM(NO_3_)_3_]^+^ ion; this ion formed by the loss of one nitrate counterion. A number of most soluble complexes were studied by NMR technique (Table 1). The signals assignments were made by the same way as for the ligand (see before). The NMR shows that both of the pyridine rings signals are affected by metal ion coordination. The signals of bipyridyl moiety in europium complex are shifted to strong field, due to paramagnetic nature of the metal ion, but the signals of the pyridine ring of amidic group are moved downfield. The samarium and lanthanum complexes also demonstrate downfield displacement of amidic pyridine signals, but the bipyridyl protons are also deshielded. This feature is mostly appearing from the effect of metal ion coordination with bipyridyl moiety, but the amidic parts of the ligand are free of metal ion binding.

The spatial positions of pyridine rings in acetonitrile solutions were elucidated by 2D NOESY spectra (Figures S9–S14 at ESI). There were no cross-peaks between methylene-protons of ethyl group and any of pyridine protons for 3c-Sm complex indicating the pyridine rings are close to each other. However, no peaks were observed between both the pyridine rings. The later shows protons of the pyridine are the neighbors of nitrogen, but not protons of the amidic pyridine rings. Such mutual arrangement of the pyridine rings was also observed in crystal (see later).

**Figure 2:**
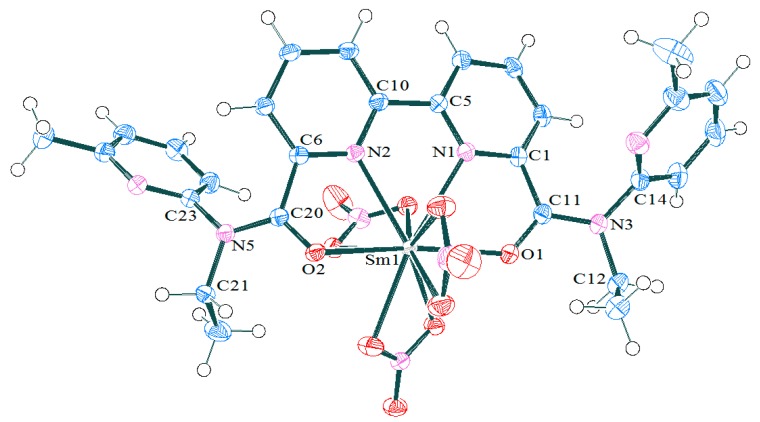
Molecular structure of Sm(**3c**)(NO_3_)_3_ showing the atom numbering scheme. Displacement ellipsoids are drawn at the 50% probability level, and H atoms are shown as small spheres of arbitrary radius.

**Figure 3:**
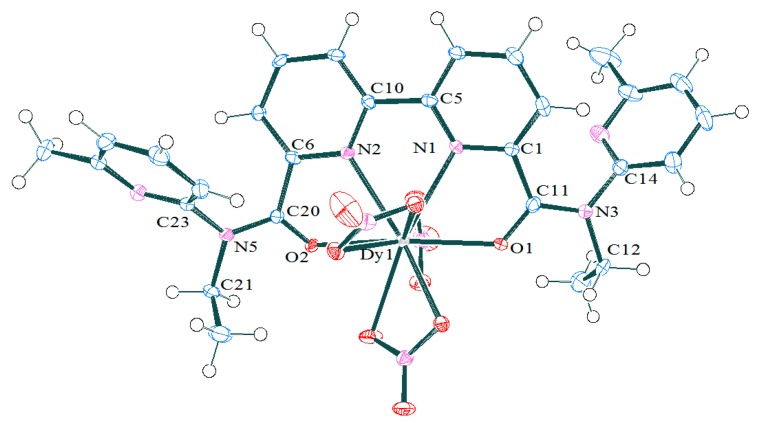
Molecular structure of Dy(**3c**)(NO_3_)_3_ showing the atom numbering scheme. Two molecules represent isomers of the complex, found for two different crystals. Displacement ellipsoids are drawn at the 50% probability level, and H atoms are shown as small spheres of arbitrary radius.

Structures of the complexes with lanthanide ions from beginning (Sm) and the end (Dy) of the row were studied by X-ray analysis (Figure 2; Figure 3, Table S1 at ESI). Selected bond lengths and angles are represented in Table 3, together with corresponding data for complexes with *N*-ethylanilide of 2,2′-bipyridyl-6,6′-dicarboxylic acid [13]. All of the complexes are mononuclear species and the composition of M_1_L_1_ found for all of the studied compounds. The metal ion is inside the pseudo-cavity of the ligand and bind with tetradentate organic ligand and three bidentate coordinated nitrate-counterions. The coordination polyhedron of the metal ions can be described as a distorted two-capped square antiprism, in which metal ions are placed above the middle of square planes. The planes are formed by N2, O2N, O1, O7N, and O5N, O1N, O8N, O2 atoms with N1 and O4N at two caps. The caps are displaced from the normal to the corresponding planes on 5−6 or 9−10°, correspondingly. This deviation originates from the structural reasons: both of atoms on the caps belong to the structurally rigid fragments, one of them to the ligand and second to the bidentate coordinated nitrate-counterion. The same reason also explains the small angle (1.6−3.3°) between square planes of antiprism. Moreover, the angle grows from samarium to dysprosium, which is associated with diminishing of ionic radius of metal and, consequently, the growth of the tensions in structurally rigid bipyridyl ligand. The inequivalence of oxygen (O1 and O2) and nitrogen (N1 and N2) positions (differences in corresponding M-O or M-N distances are represented in Table 3 and Tables S5–S19 at ESI) is one more effect of the coordination of tetradentate ligand in different positions of the square antiprism. In whole, the M-O1(2) distances for complexes of type 3 are less differentiated than was found for complexes with BPDA ligand, but M-N1(2) distances differ from each over with the same extent.

### 2.2. Stability of the Ln^III^ Complexes and Extraction Efficiency of the Ligands

**Figure 4:**
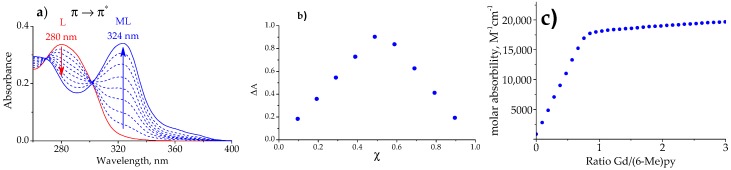
Spectrophotometric titration of **3c** with Gd(NO_3_)_3_∙6H_2_O in acetonitrile, where 1 μL of 3.82 mM titrant solution Gd(NO_3_)_3_∙6H_2_O were added to 2 mL of 2.01 × 10^−2^ mM **2c** (0–3.0 equiv.). (**a**) UV-vis titration of **2c** observed upon the addition of 0–1.0 equiv. of Gd(NO_3_)_3_∙6H_2_O at 25 °C; (**b**) the Job′s plot at 324 nm and (**c**) titration curve at 324 nm.

The stability of the complexes of lanthanides with ligand **2c** was studied in acetonitrile solution by the UV-vis titration technique (Figure 4) [12]. The UV-vis spectrum of the free ligands shows that the broad band at 280 nm corresponds to the π → π * conversion at pyridine rings. This band is bathochromic shifted when comparing with corresponding diamides bearing aniline side chain [13]. Additionally, broad peak at long wavelength descent of the band is observed for pyridineamine bearing ligands, which can be associated with side pyridine groups. After increasing the amount of metal salt addition, a new peak at 324 nm with good isobestic behavior is appear (Figure 4a). During the titration, the pyridine π-π* absorption band of the diamide was significantly enhanced by metal solution addition, showing metal coordination with the pyridine rings. The M_1_L_1_ complex composition was found for all of the studied Ln-ligand pairs by both the continuous variation method (Figure 4b) and the titration method (Figure 4c). Factor analysis shows the presence of two absorbing species that correspond to the free ligand and the complex for metal-to-ligand ratios below 10. Accordingly, the side aminopyridine rings does not take part in metal coordination for ligands bearing 2-aminopyridine unit. The stability constants (lgβ_1_) of lanthanide complexes were calculated while using the HypSpec2014 program [18] (Table 4). The titration was repeated twice for several metal ions and the results were within the confidence interval. The lgβ values are within 5.5−7.0 intervals; these results indicate the electron withdrawing nature of the pyridine rings. The stability of the complexes are less than aromatic diamides possessing electron donating substituents [12], but slightly higher than for BPDA ligand [13].

**Figure 5:**
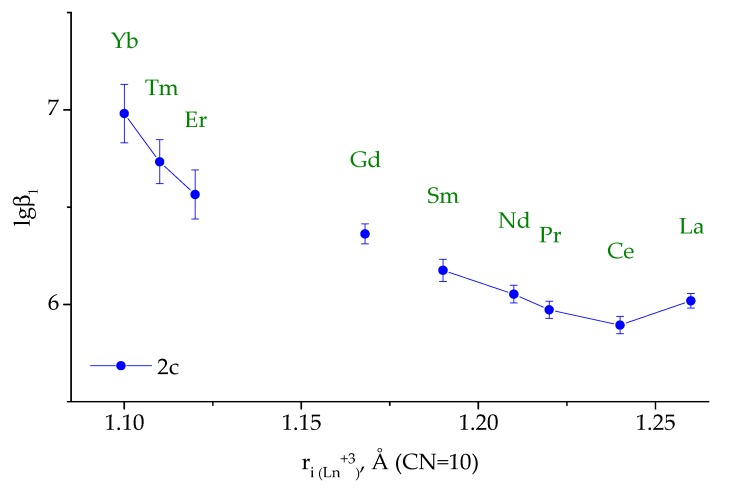
The stability constants (lgβ) of complexes of ligand **2c** with Ln^III^ as a function of ionic radii of ions.

The stability of the complexes steadily drops down from the ytterbium to lanthanum (Figure 5, Table 4). The metal-to-metal selectivity seems to depend on the electron withdrawing properties of the pyridine ring at amidic moiety, due to the changing of the pyridine to methylpyridine amidic side group leading to the diminishing of differences between the smallest and the largest stability constants in the series.

The 2-aminopyridine bearing diamide was evaluated for their ability to extract Am(III) and Eu(III) ions from 5M HNO_3_ in nitrobenzene. The solubility of the diamide **2c** is equal to the BPDA [13], however it shows significantly fewer metal extraction efficiency and selectivity. The separation factors for Am(III) and Eu(III) are 0.01 and 0.004, correspondingly. Hence, the substitution of the amidic part of the molecule by electron sink pyridine rings leads to a diminishing of the extraction strength.

### 2.3. Photophysical Properties of Ln(3)(NO_3_)_3_ Complexes

**Figure 6:**
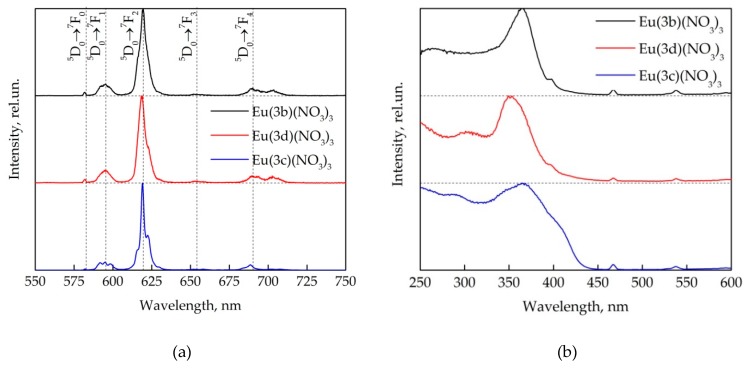
(**a**) Luminescence emission spectra of europium complexes in solid state, excitation wavelength λ_ex_ = 320 nm and (**b**) luminescence excitation spectra of europium complexes in solid state, emission wavelength λ_em_ = 618 nm.

Luminescence emission spectra of europium complexes represented narrow peaks that are characteristic for europium ion emission at the visible and near-IR regions [19]. We measured the photophysical properties of three europium complexes with 2-pyridyl-based ligands (**3b** and **3c**) and 4-pyridyl-bering ligand (**3d**). Integral luminescence intensity of Eu(**3c**)(NO_3_)_3_ complex was 5.5 times higher than the intensity of Eu(**3b**)(NO_3_)_3_ and Eu(**3d**)(NO_3_)_3_ complexes (Figure 6).

The peaks in luminescence emission spectra of europium complexes corresponded to ^5^D_0_→^7^F_J_ (J = 0−4) transitions [12,13,19,20]. The most intensive peak of luminescence spectra corresponds to ^5^D_0_→^7^F_2_ transition and it had maximum at the wavelength 619.4 nm for Eu(**3b**)(NO_3_)_3_ complex, 618.4 nm for Eu(**3d**)(NO_3_)_3_ complex, and 619.0 nm for Eu(**3c**)(NO_3_)_3_ complex; the integral intensity of this peak was more than half total europium luminescence: 69% for Eu(**3b**)(NO_3_)_3_ and Eu(**3d**)(NO_3_)_3_ complexes and 75% for Eu(**3c**)(NO_3_)_3_ complexes. The shape of this spectral line differed for all three studied complexes and deviated from the Gaussian, due to the observation of the Stark structure of the europium ion energy levels under the influence of the environment of the central ion. The luminescent peak that corresponds to magnetic dipole transition ^5^D_0_ → ^7^F_1_ located at the wavelength 590−605 nm.

We calculated the asymmetry coefficient R = I(^5^D_0_→^7^F_2_)/I(^5^D_0_→^7^F_1_) as the ratio of the luminescence emission integral intensities of peaks that corresponds to ^5^D_0_→^7^F_2_ and ^5^D_0_→^7^F_1_ transitions (Table 5), which allows for estimating the deviation of europium from the inverse center (R = 1 for central symmetry) [19,20]. This coefficient was approximately the same and it equaled to 5.52, 5.49, and 5.77 for Eu(**3b**)(NO_3_)_3_, Eu(**3d**)(NO_3_)_3_, and Eu(**3c**)(NO_3_)_3_ complexes, respectively. Thus, the asymmetry ratio did not depend on the position of the nitrogen atom in the substituent; however, the addition of an additional methyl substituent leads to a slight increase in the asymmetry coefficient. 

The radiative luminescence lifetime of europium complexes can be defined as the inverse of the Einstein coefficients of spontaneous emission A(Ψ_J_,Ψ_J′_). The search for this value for a europium ion can be simplified to the following form [21]:

(1) $$A\left(\Psi_{j},\Psi_{j\prime }\right)=\frac{1}{\tau_{rad}}=A_{MD,0}n^{3}\frac{I_{\Sigma }}{I_{MD}}$$

where *A_MD,0_*—constant equals to 14.65 cm^−1^, n—refractive index (n = 1.55 for samples in solid state), and *I_Σ_/I_MD_*—total luminescence intensity and intensity of luminescence peak corresponding to magnetic dipole transition ratio. It equaled 2.28 ms for Eu(**3b**)(NO_3_)_3_ complex, 2.29 ms for Eu(**3d**)(NO_3_)_3_ complex and 2.38 ms for the Eu(**3c**)(NO_3_)_3_ complex.

The observed luminescence lifetime was determined while using phosphorescence decay of energy transition from ^5^D_0_ level to ^7^F_2_ level, since the kinetics correspond to monoexponential decay. For Eu(**3b**)(NO_3_)_3_ and Eu(**3d**)(NO_3_)_3_ complexes, it was quiet low and equaled to 0.75 and 0.87 ms, respectively. However, under adding of methyl substituent the observed luminescence lifetime has doubled, so for the Eu(**3c**)(NO_3_)_3_ complex, it equaled to 1.36 ms.

The internal luminescence quantum yield of europium complexes was calculated as the ratio of the observed and radiative luminescence lifetimes Q_Eu_ = τ_obs_/τ_rad_. It was the lowest for Eu(**3b**)(NO_3_)_3_ complex and equaled 33%. For Eu(**3d**)(NO_3_)_3_ complex it equaled 40%, the highest luminescence internal quantum yield was for Eu(**3c**)(NO_3_)_3_ complex and it equaled 57%.

The excitation spectra of europium complexes help to determine the path of energy migration in the complex. A simplified scheme of energy transfer in REE complexes can be described in three stages: light absorption by the ligand (^0^S→^1^S*), and then the transfer of energy through the triplet state of the ligand (^1^S*→^3^T*) on rare earth ion (^3^T*→Ln*) and the lanthanide characteristic luminescence emission [19]. The luminescence emission of studied europium complexes was mainly excited by the absorption of light by the ligand (broad band in UV spectral region), but also the direct excitation of high-lying energy levels of the europium ion was observed (narrow peaks at 370−600 nm). The shape of luminescence excitation spectra of Eu(**3b**)(NO_3_)_3_ and Eu(**3d**)(NO_3_)_3_ complexes was quite similar, but for Eu(**3d**)(NO_3_)_3_ complex hypsochromic shift of the excitation spectrum maximum by 16 nm was observed. The luminescence emission maximum of Eu(**3c**)(NO_3_)_3_ complex was at the same wavelength as for the Eu(**3b**)(NO_3_)_3_ complex, but this peak was much wider.

The external luminescence quantum yield was determined for Eu(**3b**)(NO_3_)_3_ и Eu(**3c**)(NO_3_)_3_ complexes dissolved in acetonitrile. We prepared solutions of complexes with concentration of (0.4−7.0) × 10^−5^ mol/L, and then we used reference dye method (Rhodamide 6G as etalon) to determine the external luminescence quantum yield. It equaled 18% for Eu(**3c**)(NO_3_)_3_ complex, for Eu(**3b**)(NO_3_)_3_ complex—practically four times lower, 5%. 

The luminescence quantum yields of studied europium complexes with diamides of 2,2′-bipyridyl-6,6′-dicarboxylic acid are slightly lower (in case Eu(3b)(NO_3_)) or 1.75 and 2.25 times higher (in case Eu(3b)(NO_3_) and Eu(3c)(NO_3_), respectively) when compared with europium complex with free diamides of 2,2′-bipyridyl-6,6′-dicarboxylic acid ligand [13] and more than ten times higher when compared lanthanide complexes of 2,2′-bipyridyl-6,6′-dicarboxylic dimethylanilides [12]. This fact suggests that the process of energy transfer in complexes of this type probably occurs a little differently. We assume that the energy from the ligand triplet level is first transferred to the high-energy ^5^D_1_ or ^5^D_2_ energy level, after this nonradiative energy transfer to the ^5^D_0_ level take place and, finally, luminescence, in these europium complexes [22]. The energy of ligand triplet level determines this fact, which was determined while using the phosphorescence of gadolinium with 6-methyl-2-pyridylamide of 2,2′-bipyridyl-6,6′-dicarboxylic acid at 77 K (E(Tr) = 21978 cm^−1^): energy difference between ligand triplet level and the europium ^5^D_0_ level of the is too large. The energy transfer efficiency from the ligand to europium should be weak and, therefore, the luminescence quantum yield of the europium complex should be low; however, we observe the opposite.

The luminescence lifetimes of europium complexes were approximately the same as the other europium complexes with diamides of 2,2′-bipyridyl-6,6′-dicarboxylic acid. The radiative luminescence lifetimes and internal luminescence quantum yields were also practically the same. The asymmetry coefficient of europium complexes was 25% higher as compared to other previously studied complexes. This suggests that the europium ion under the influence of the immediate environment undergoes a significant deviation from the inverse center.

## 3. Materials and Methods

### 3.1. General

The NMR spectra were measured with a BRUKER AVANCE-600 MHz and AVANCE-400 MHz NMR spectrometers (Bruker Corporation, Billerica, Massachusetts, USA) at 24 °C. The IR spectra were recorded with a Varian 640t FTIR spectrometer (Varian Medical Systems, Palo Alto, California, USA) with samples in KBr pellets. The mass spectra were obtained with a MALDI-TOF Reflex 3 instrument (BRUKER (Bruker Corporation, Billerica, Massachusetts, USA)) in the positive ion mode (UV laser, 337 nm), without use of matrix. All of the reagents and solvents were obtained from commercial sources. The Acetonitrile (99.95%, Biosolve BV (Biosolve Chemicals, Dieuze, France)) was dried over molecular sieves (zeolite KA, 3 Å, balls, diameter 1.6–2.5 mm) prior to use. The water content was estimated as 40 ± 5 ppm by Karl Fisher titration (Mettler Toledo, C20, coulometric KF titrator (Mettler-Toledo, Inc., Columbus, Ohio, USA). Lanthanide metal nitrates Ln(NO_3_)_3_·6H_2_O (*n* = 4–6, purity > 99%) were stored in a closed container over silica gel balls. The stock solutions of ligands and metal salts were prepared by weighing the amounts of the respective chemicals and dissolving them in acetonitrile.

### 3.2. Spectrophotometric Titration

Ultraviolet–visible (UV-vis) spectra were recorded at ambient temperature (24.5 ± 1.0 °C) in the wavelength region of 260–500 nm (1 nm interval) on a Hitachi U-1900 spectrophotometer (Hitachi, Tokyo, Japan) while using 10 mm path length quartz cells. The implementation of the Beer–Lambert law was determined for ligand within the range 0.01–0.1 mM. The method of continuous variation determined the binding stoichiometry of one-step complex formation between two different molecules (Job′s plot). The solutions of ligand **2c** and metal salt were prepared at a concentration of ≈ 0.1 mM. For the spectrophotometric titration, solution of ligand **2c** was prepared ca. 20–45 μM. A titrant solutions Ln(NO_3_)_3_·6H_2_O (ca. 3–7 mM) was prepared by dissolution of a sample of Ln nitrate hydrate in solution of ligand **2c**. A 2 mL of solution of ligand **2c** was titrated with 1 μL Ln(NO_3_)_3_·6H_2_O solution. The kinetic experiments showed that the complexation reaction is fast and the absorbance becomes stable within 1–2 s. 

### 3.3. Luminescent Measurements

The luminescence emission spectra were recorded while using Hitachi F-7000 luminescence spectrometer (Hitachi, Tokyo, Japan) at room temperature. The reflection geometry was used for solid samples and 90°-geometry was used for acetonitrile solutions placed in standard quartz cuvette with optical path length 10 mm). The excitation wavelength was set at 320 nm, registration was performed within he spectral region 350–800 nm with spectral interval 0.2 nm. The luminescence excitation spectra were registered at 618 nm with excitation in spectral range 250−600 nm with spectral interval 1 nm. The scan speed was –1200 nm/min., spectral slits (excitation monochromator and emission monochromator) were 5 × 1 nm for solid state measurements and 5 × 5 nm for acetonitrile solutions. PMT voltage was 400 V. The absorption spectra of complexes in acetonitrile solutions were determined while using spectrophotometer Hitachi U-1900 in standard quartz cuvette with optical path length 10 mm.

### 3.4. Reductive Amination Using Sodium Triacetoxyborohydride

3 mL of acetaldehyde and two drops of glacial acetic acid were added to the solution of 0.05 mol of the corresponding 2-aminopyridine in 350 mL of 1,2-dichloroethane. The resulting mixture was stirred for one hour and 15.9 g (0.075 mol) of sodium triacetoxyborohydride was added. The reaction mixture was stirred at room temperature for 48 h and treated by saturated solution of potassium carbonate until the gas was released. The organic layer was separated and the aqueous layer was extracted with 1,2-dichloroethane (2 × 50 mL). Combined organic extracts were dried with anhydrous potassium carbonate. The corresponding ethylamine was obtained after the evaporation of the solvent.

*2-Ethylamino-6-metylpyridine* (**1a**) [23,24]. Yield 5.2 г (80%) of pale yellow oil. Bp 98−100 °C/9 torr. ^1^H; NMR: 1.2 (tr, *J* = 7.5 Hz, 3 H) 2.4 (s, 1H) 3.3 (quadr, *J* = 7.5, Hz, 2 H) 6.7 (d, *J* = 8,5, Hz, 1 H) 7.0 (d, *J* = 7.3 Hz, 1 H) 7.5 (dd, *J* = 8.5, 7.3, Hz, 1 H).

*2-Ethylamino-6-methylcarboxypyridine* (**1b**). Yield 4.7 г (52%) of gray powder. ^1^H NMR: 1.3 (tr, *J* = 7.5, Hz, 3 H) 3.6 (quadr, *J* = 7.5, Hz, 2 H) 4.1 (s, 3 H) 6.7 (d, *J* = 8.9 Hz, 1 H) 7.4 (d, *J* = 7.7 Hz, 1 H) 7.7 (dd, *J* = 8.9, 7.7 Hz, 1 H).

### 3.5. Alkylation and Hydrolysis of the N-acetylaminopyridines

#### 3.5.1. General Method 1

Sodium hydride (2.12 g 60% suspension in mineral oil, 0.075 mol) was added to 200 mL of absolute DMF when cooled in a water bath and stirred. The resulting mixture was stirred for 10 min. and after that 6.8 g (0.05 mol) of the corresponding acetylaminopyridine was added in small portions. At the end of the addition, the mixture was stirred for 30 min. under the same conditions. A solution of 4.5 mL (0.06 mol) ethyl bromide in 10 mL of absolute DMF was added dropwise to the reaction mixture. The reaction was stirred for 2 h and then left overnight. The mixture was neutralized by addition of acetic acid (4 mL in 10 mL of water) and the solvent was evaporated in vacuo. The residue was extracted with CH_2_Cl_2_ (4 × 50 mL) and the solvent was removed. The residue was boiled for 5 h in 100 mL of 25% hydrochloric acid. Water was evaporated to dryness; the residue was suspended in 250 mL of CH_2_Cl_2_ and then treated with a saturated solution of K_2_CO_3_, until the end of the gas release. The organic phase was separated and the aqueous phase was extracted with CH_2_Cl_2_ (2 × 25 mL). Combined organic extracts were dried with anhydrous K_2_CO_3_. Target *N*-ethylaminopyridine was obtained after the removal of the solvent.

#### 3.5.2. General Method 2

In a glass vial was mixed 3 g (0.02 mol) of the hydrochloride of 4-chloropyridine, 3 mL of 70% aqueous ethylamine and 15 mL of acetonitrile. The vial was sealed and heated at 115 °C; for 6 h. After cooling the reaction mixture, the vial was opened, and the solvent and excess ethylamine were removed in a vacuum. The residue was suspended in 100 mL of CH_2_Cl_2_ and treated with a saturated potash solution until the end of gas evolution. The organic phase was separated, the aqueous phase was extracted with CH_2_Cl_2_ (2 × 25 mL). Combined organic extracts were dried with anhydrous K_2_CO_3_. After the removal of the solvent, pure 4-ethylaminopyridine as yellow oil was obtained. Yield 2 g (82%).

*2-Ethylaminopyridine* (**1c**) [24,25,26,27]: Yield 3.8 g (63%) of pale yellow oil. ^1^H NMR (400 MHz, CHLOROFORM-D) ppm 1.2 (d, *J* = 7.2 Hz, 3 H) 3.3 (qd, *J* = 7.2, 5.6 Hz, 2 H) 4.6 (s, 1 H) 6.3 (d, *J* = 8.4 Hz, 1 H) 6.5 (ddd, *J* = 7.2, 5.0, 0.9 Hz, 1 H) 7.4 (ddd, *J* = 8.5, 7.0, 1.8 Hz, 1 H) 8.0 (dt, *J* = 5.0, 0.9 Hz, 1 H).

*3-Ethylaminopyridine* (**1d**) [15,28,29]: Yield 1.3 g (21%) of yellow oil. ^1^H NMR (400 MHz, CHLOROFORM-D) ppm 1.1 (t, *J* = 7.2, Hz, 3 H) 2.9 (quadr, 2 H) 6.8 (d, *J* = 8.3 Hz, 1 H) 7.0 (m, 1 H) 7.9 (m, 1 H) 8.2 (s, 1 H).

*4-Ethylaminopyridine* (**1e**) [30,31]: Yield 2.1 g (38%) of yellow oil. ^1^H NMR (400 MHz, CHLOROFORM-D) ppm1.2 (t, *J* = 7.2 Hz, 3 H) 3.1 (quadr, *J* = 7.2 Hz, 2 H) 6.4 (d, *J* = 6.1 Hz, 2 H) 8.1 (d, *J* = 5.4 Hz, 2 H).

### 3.6. Synthesis of 2,2′-bipyridyl-6,6′-dicarboxylic acid diamides (General Method)

2,2′-Bipyridyl-6,6′-dicarboxylic acid (2.44 g, 0.01 mol) in 15 mL of thionyl chloride with 0.1 mL of DMF was boiled for 2.5 h. Thionyl chloride was removed by distillation and the remaining chloroanhydride was dried in a vacuo and dissolved in 30 mL of absolute tetrahydrofuran. The resulting solution was added portionwise to the mixture of 0.022 mol of the corresponding amine, 5 mL of triethylamine and 50 mL of absolute tetrahydrofuran with stirring. The resulting mixture was stirred for 4−5 h at 40−50 °C and then left overnight. An equal volume of water was added to the reaction mixture and the organic layer was separated. The aqueous fraction was extracted with diethyl ether (3 × 25 mL). The combined organic fractions were washed with water and then dried over anhydrous Na_2_SO_4_. After evaporation of the solvents, the residue was treated with a minimal amount of ethyl acetate. The precipitate was filtered out, washed with cold ethyl acetate, hexane, and then dried on air.

*N,N′-bis(6-methylpyridin-2-yl)-2,2′-bipyridine-6,6′-dicarboxamide* (**2a**): Yield 1.78 g (42%). MALDI-TOF MS: *m*/*z* (%) = 425 ([M + H]^+^), 447 ([M + Na]^+^), 453 ([M + K]^+^). ^1^H NMR (400 MHz, CHLOROFORM-D) d ppm 2.6 (s, 6 H) 7.0 (dd, *J* = 7.6, 1.5 Hz, 2 H) 7.7 (t, *J* = 7.8 Hz, 2 H) 8.2 (dd, *J* = 7.5, 6.7 Hz, 2 H) 8.3 (dd, *J* = 8.1, 1.2 Hz, 2) 8.4 (br.s, 2 H) 8.8 (dd, *J* = 6.7, 2.1 Hz, 2 H) 10.3 (s, 2 H). 13C NMR (101 MHz, CHLOROFORM-*d*) 24.1 (CH3); 111.1 (2Py_Am_); 119.2 (2Py_Am_); 123.8 (2Py_Ac_); 124.5 (5Py_Ac_); 127.6 (5Py_Ac_) 138.8 (4Py_Ac_) 139.1 (4Py_Am_); 140.6 (6Py_Ac_); 150.1 (6Py_Am_); 155.3 (2Py_Ac_); 161.2 (2Py_Am_); 162.1 (C=O).

*N,N′-diethyl-N,N′-dipyridin-2-yl-2,2′-bipyridine-6,6′-dicarboxamide* (**2b**): Yield 1.53 g (34%). MALDI-TOF MS *m*/*z*: 453 ([M + H]^+^), 476 ([M + Na]^+^), 492 ([M + K]^+^.). 1H NMR (600 MHz, CHLOROFORM-*d*) ppm 1.27 (t, *J* = 7.06 Hz, 3 H) 4.18 (q, *J* = 7.15 Hz, 2 H) 6.93 (d, *J* = 7.34 Hz, 1 H) 6.96 (ddd, *J* = 7.34, 4.86, 0.73 Hz, 1 H) 7.45 (td, *J* = 7.79, 1.56 Hz, 1 H) 7.49 (d, *J* = 7.15 Hz, 1 H) 7.65 (t, *J* = 7.79 Hz, 1 H) 7.78 (dd, *J* = 7.70, 0.92 Hz, 1 H) 8.32 (dd, *J* = 4.77, 1.19 Hz, 1 H). 13C NMR (151 MHz, CHLOROFORM-*d*) ppm 13.22 (CH3) 43.96 (CH2) 121.06 (3Py_Am_) 121.34 (3Py_Ac_) 121.66 (5Py_Am_) 124.69 (5Py_Ac_) 137.28 (4Py_Ac_) 137.30 (4Py_Am_) 148.49 (6Py_Am_) 152.37 (6Py_Ac_) 153.05 (2Py_Ac_) 156.30 (2Py_Am_) 167.91 (C=O).

*N,N′-diethyl-N,N′-bis(6-methylpyridin-2-yl)-2,2′-bipyridine-6,6′-dicarboxamide* (**2c**): Yield 2.59 г (54%). Mass-spectrum (MALDI-TOF) *m*/*z*: 481 ([M + H]^+^), 503 ([M + Na]^+^), 519 ([M + K]^+^). 1H NMR (600 MHz, CHLOROFORM-*d*) ppm 1.26 (t, *J* = 7.06 Hz, 3 H, CH3) 2.41 (s, 3 H, CH3_Py_) 4.17 (q, *J* = 7.15 Hz, 2 H, CH2) 6.64 (br. d., *J* = 8.38 Hz, 1 H, 5Py_Am_) 6.80 (d, *J* = 7.58 Hz, 1 H, 3Py_Am_) 7.30 (t, *J* = 7.95 Hz, 1 H, 4Py_Am_) 7.51 (br. d., *J* = 8.19 Hz, 1 H, 3Py_Ac_) 7.64 (t, *J* = 7.70 Hz, 1 H, 4Py_Ac_) 7.76 (d, *J* = 7.40 Hz, 1 H, 5Py_Ac_). 13C NMR (151 MHz, CHLOROFORM-*d*) ppm 12.99 (CH3) 23.92 (CH3_Py_) 43.60 (CH2) 118.23 (2Py_Am_) 120.45 (2Py_Ac_) 121.37 (5Py_Am_) 124.43 (5Py_Ac_) 136.90 (4Py_Ac_) 137.37 (4Py_Am_) 152.47 (6Py_Ac_) 152.90 (6Py_Am_) 155.30 (2Py_Ac_) 157.65 (2Py_Am_) 167.65 (C=O).

*N,N′-diethyl-N,N′-dipyridin-4-yl-2,2′-bipyridine-6,6′-dicarboxamide* (**2d**): Yield 2.21 g (49%). MALDI-TOF MS *m*/*z*: 453 ([M + H]^+^.), 476 ([M + Na]^+^.), 492 ([M + K]). 1H NMR (400 MHz, CHLOROFORM-*d*) ppm 1.28 (t, *J* = 7.09 Hz, 3 H) 4.06 (q, *J* = 7.09 Hz, 2 H) 6.98 (d, *J* = 6.11 Hz, 2 H) 7.32 (d, *J* = 7.83 Hz, 1 H) 7.70 (t, *J* = 7.83 Hz, 1 H) 7.81 (dd, *J* = 7.58, 0.73 Hz, 1 H) 8.44 (d, *J* = 5.87 Hz, 2 H). 13C NMR (101 MHz, CHLOROFORM-*d*) ppm 13.11 (CH3) 45.44 (CH2) 121.36 (3,5Py_Am_) 122.02 (3Py_Ac_) 124.99 (5Py_Am_) 137.73 (4Py_Ac_) 150.53 (2,6Py_Am_) 151.48 (6Py_Ac_) 153.16 (2Py_Ac_) 167.18 (C=O).

*N,N′-diethyl-N,N′-dipyridin-3-yl-2,2′-bipyridine-6,6′-dicarboxamide* (**2e**): Yield 127mg (20%). MALDI-TOF MS *m*/*z*: 453 ([M + H]^+^.), 476 ([M + Na]^+^.), 492 ([M + K]^+^.). 1H NMR (400 MHz, CHLOROFORM-*d*): 1.2 (t, *J* = 7.1 Hz, 6 H) 4.0 (q, *J* = 7.1 Hz, 4 H) 7.1 (s, 2 H) 7.4 (m4 H) 7.7 (m, 4 H) 8.3 (d, 2 H) 8.4 (d, 2 H). 13C NMR (101 MHz, CHLOROFORM-*d*) 12.4 (CH3), 46.4 (CH2), 121.2 (5Py_Ac_, 5Py_Am_), 122.9 (3Py_Ac_), 134.1 (4Py_Am_), 137.0 (4Py_Ac_), 139.7 (3Py_Am_), 146.9 (2,6Py_Am_), 151.5 (6Py_Ac_), 152.7 (2Py_Ac_), 168.8 (C=O).

### 3.7. Synthesis of Complexes with Lanthanides Nitrates (General Method)

A corresponding ligand (70 mg, 15.5 × 10^−5^ mol) was dissolved in 2 mL of dry acetonitrile. 15.5 × 10^−5^ mol of trinitrate of the corresponding metal was added to the resulting solution. The solution obtained was heated to 80 °C and cooled to room temperature. The precipitate was filtered out, washed with acetonitrile (1−1.5 mL), and then dried in air to a constant mass.

*N,N′-diethyl-N,N′-dipyridin-2-yl-2,2′-bipyridine-6,6′-dicarboxamideeuropium trinitrate* Eu(**3b**)(NO_3_)_3_: Yield 101 mg (83%). Mass-spectrum (MALDI-TOF), *m*/*z*: 700 [M-NO_3_]^+^. Found, %: C 39.42, H 3.29, N 15.85. Calculated for C_26_H_24_EuN_9_O_11_·, %: C 39.50, H 3.06, N 15.95.

*N,N′-diethyl-N,N′-bis(6-methylpyridin-2-yl)-2,2′-bipyridine-6,6′-dicarboxamidelanthanum trinitrate* La(**3c**)(NO_3_)_3_: Yield 100 mg (85%). Mass-spectrum (MALDI-TOF) *m*/*z*: 743 [M-NO_3_]^+^. Found, %: C 39.70, H 3.89, N 14.73. Calculated for C_28_H_28_LaN_9_O_11_·2(H_2_O), %: C 39.96, H 3.83, N 14.98.

*N,N′-diethyl-N,N′-bis(6-methylpyridin-2-yl)-2,2′-bipyridine-6,6′-dicarboxamidesamarium trinitrate* Sm(**2c**)(NO_3_)_3_: Yield 74 mg (62%). Mass-spectrum (MALDI-TOF), *m*/*z*: 755 [M-NO_3_]^+^. Found, %: C 39.86, H 3.61, N 14.74. Calculated for C_28_H_28_SmN_9_O_11_·2(H_2_O), %: C 39.43, H 3.78, N 14.78. 1H NMR (400 MHz, ACETONITRILE-*d*_3_) ppm 1.44 (t, *J* = 6.11 Hz, 3 H) 2.55 (br. s., 3 H) 4.48 (q, *J* = 6.85 Hz, 2 H) 6.91 (d, *J* = 8.07 Hz, 1 H) 7.02 (d, *J* = 7.58 Hz, 1 H) 7.33 (d, *J* = 6.60 Hz, 1 H) 7.63 (t, *J* = 7.83 Hz, 1 H) 7.97 (t, *J* = 7.82 Hz, 1 H) 8.35 (d, *J* = 7.34 Hz, 1 H).

*N,N′-diethyl-N,N′-bis(6-methylpyridin-2-yl)-2,2′-bipyridine-6,6′-dicarboxamideeuropium trinitrate* Eu(**3c**)(NO_3_)_3_: Yield 66 mg (55%). Mass-spectrum (MALDI-TOF), m/z: 756 [M-NO_3_]^+^. Found, %: C 39.16, H 3.65, N 14.50. Calculated for C_28_H_28_EuN_9_O_11_·2(H_2_O), %: C 39.35, H 3.77, N 14.75. 1H NMR (400 MHz, ACETONITRILE-*d*_3_) ppm 0.53 (br. s., 3 H, CH3) 3.04 (br. s., 2 H, CH2) 4.25 (br. s., 1 H) 4.34 (br. s., 1 H) 5.84 (br. s., 1 H) 7.25 (br. s., 1 H) 7.83 (br. s., 1 H) 7.97 (br. s., 1 H). 13C NMR (151 MHz, ACETONITRILE-*d*_3_) ppm 10.90, 24.15, 45.71, 93.76, 95.07, 119.61, 124.84, 140.45, 145.29, 148.26, 150.21, 160.72, 163.44, 172.03.

*N,N′-diethyl-N,N′-bis(6-methylpyridin-2-yl)-2,2′-bipyridine-6,6′-dicarboxamidedysprosium trinitrate* Dy(**3c**)(NO_3_)_3_: Yield 88 mg (73%). Mass-spectrum (MALDI-TOF) *m*/*z*: 767 [M-NO_3_]^+^. Found, %: C 40.43, H 3.45, N 14.68. Calculated for C_28_H_28_N_9_O_11_Dy, %: C 40.56, H 3.40, N 15.20.

*N,N′-diethyl-N,N′-dipyridin-4-yl-2,2′-bipyridine-6,6′-dicarboxamideeuropium trinitrate* Eu(**3d**)(NO_3_)_3_: Yield 61 mg (50%). Mass-spectrum (MALDI-TOF), *m*/*z*: 700 [M-NO_3_]^+^. Found, %: C 39.37, H 2.94, N 15.79. Calculated for C_26_H_24_EuN_9_O_11_, %: C 39.50, H 3.06, N 15.95.

## Figures and Tables

**Table 1:** Chemical shifts (in ppm) of aromatic protons for **2c** ligand and their complexes.

Compound Number and Structure 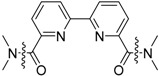		Acid Moiety			Amidic Moiety		
		3Py	4Py	5Py	3Py′	4Py′	5Py′
**2a**	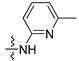	8.3 d	8.4 br.s	8.8 dd	7.0 dd	7.7 t	8.2 dd
**2b**	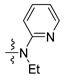	7.49 d	7.65 t	7.78 dd	6.93 d	7.45 td	6.96 ddd
**2c**	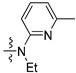	7.51 br.d	7.64 t	7.76 d	6.64 br.d	7.30 t	6.80 d
**2d**	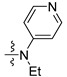	7.32 d	7.70 t	7.81 d	8.44 d	-	8.44 d
**2e**	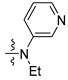	7.4	7.7	7.7	-	8.3	8.4
Sm(**3c**)(NO_3_)_3_		8.35 d	7.97 t	7.02 d	7.33 d	7.63 t	6.91 d
Eu(**3c**)(NO_3_)_3_		4.25br.s	5.84 br.s	4.34 br.s	7.25 br.s	7.83 br.s	7.97 br.s

**Table 2:** Comparison of the several interatomic distances (Å) and angles (°) of the primary diamide **2a** with secondary diamide analogs.

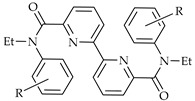 BPDA (R = H); F-BPDA (R = 2F); Bridge-BPDA (R = 3,4-(CH_2_)_4_				
Bonds/Angles	2a	F-BPDA [11]	Bridge-BPDA [11]	BPDA [13]
C1-C2	1.512(2)	1.519(3)	1.502(2)	1.511(2)
C1-O1	1.224(2)	1.239(3)	1.222(2)	1.230(1)
C1-N2	1.361(2)	1.371(3)	1.358(2)	1.357(2)
N2-H	0.85(2)	-	-	-
N2-C7	1.411(2)	1.447(3)	1.436(2)	1.435(2)
N1-C6-C6′-N1′	180.0(1)	180.0(2)	180.0(2)	180.0(1)
N1-C2-C1-O1	177.2(1)	142.0(2)	-151.4(2)	138.7(1)
Angle between amidic Ar and Py planes	23.19	61.0	68.8	63.8
Angle between O=CNC and Ar-plane	20.05	63.3	68.4	64.6
Angle between O=CNC and Py-plane	3.64	35.7	24.7	37.5

**Table 3:** Comparison of the interatomic distances (Å) and angles (°) in the coordination sphere of the metal ions for complexes of **3c** with 2,2′-bipyridyl-6,6′-dicarboxylic acid (BPDA) ligand complexes.

**Bonds/Angles**	NdBPDA(NO_3_)_3_ [13]	TbBPDA(NO_3_)_3_ [13]	Sm(3c)(NO_3_)_3_	Dy(3c)(NO_3_)_3_	
M–O1 M–O2	2.481(1) 2.409(1)	2.402(3) 2.325(3)	2.447(2) 2.405(2)	2.424(3) 2.387(3)	2.407(4) 2.346(5)
*d*M-O1–*d*M-O2	0.072(1)	0.077(3)	0.042(2)	0.037(3)	0.61(5)
M–N1 M–N2	2.605(2) 2.683(2)	2.514(3) 2.611(3)	2.553(2) 2.649(2)	2.529(3) 2.636(3)	2.497(6) 2.603(5)
*d*M–N1–*d*M–N2	0.078(2)	0.097(3)	0.096(2)	0.107(3)	0.106(6)
O1–M–O2	161.48(5)	154.66(8)	158.78(6)	157.36(9)	155.9(2)
N1–M–N2	61.30(5)	62.65(6)	61.64(6)	61.9(1)	62.4(2)
Deviation of caps from normal to the square planes	15		4.821 9.277	5.531 9.456	6.749 10.049
Angle between two planes of antiprism	1.2–1.7		1.67	2.23	3.32
Mean deviation of atoms from the square planes	-		0.089 0.189	0.086 0.184	0.073 0.174

**Table 4:** Log *β*_1_ values for the stability of trivalent lanthanides ions with **2c** ligand in acetonitrile.

Ln^3+^	La	Ce	Pr	Nd	Sm	Gd	Er	Tm	Yb
**2c**	6.02 ± 0.04	5.89 ± 0.04	5.97 ± 0.04	6.05 ± 0.05	6.18 ± 0.06	6.36 ± 0.05	6.6 ± 0.1	6.7 ± 0.1	7.0 ± 0.1
**2,5-diMeBPDA** [12]	6.11	6.42	6.59	6.82	6.71	6.30	6.7	6.33	6.5
**2,4-diMeBPDA** [12]	6.45	6.41	7.11	7.36	6.96	6.59	7.29	6.91	7.05
**3,4-diMeBPDA** [12]	5.6	6.91	7.6	8.3	7.6	6.76	7.35	7.28	6.9
**3,5-diMeBPDA** [12]	6.0	6.51	7.19	7.6	6.89	6.69	7.3	7.23	6.86
**BPDA** [13]	6.2	5.6	5.9	5.9	5.7	5.7	5.4	5.9	5.6

**Table 5:** Luminescent characteristics of europium complex.

Europium Complex	Eu(3b)(NO_3_)_3_	Eu(3d)(NO_3_)_3_	Eu(3c)(NO_3_)_3_
Wavelength of absorption maximum, nm	322	320	323
Asymmetry ratio	11.95	11.94	11.90
Observed luminescence lifetime, ms	1.30	1.21	1.23
Radiative lifetime, ms	1.91	1.91	1.92
Internal luminescence quantum yield, %	65	63	64
External luminescence quantum yield, %	5	14	18

## Supplementary Materials

The following are available online at https://www.mdpi.com/1420-3049/25/1/62/s1, X-Ray parameters (Table S1) and structure data (Table S2−S19), UV-vis Titration Data in CH_3_CN (Table S1; Table S2) and 1D and 2D NMR (Figure S1−S14).
