# Supplementary material for: Effect of Heterocyclic Ring on LnIII Coordination, Luminescence and Extraction of Diamides of 2,2′-Bipyridyl-6,6′-Dicarboxylic Acid
Source: Molecules. 2019 Dec 23;25(1):62. doi: 10.3390/molecules25010062 (PMC6983261; doi:10.3390/molecules25010062)
Supplement: Supplementary file 1 [file molecules-25-00062-s001.pdf]

Supplementary Information

for

Effect of heterocyclic ring on Ln^{III} coordination, luminescence and extraction of diamides of 2,2′-bipyridyl-6,6'-dicarboxylic acid

Nataliya E. Borisova [1*], Alexey V. Ivanov [1], Tsagana B. Sumyanova [1], Anastasia V. Kharcheva [1,2], Pert I. Matveev [1] and Svetlana V. Patsaeva [2]

[1] Chemistry Department, M. V. Lomonosov Moscow State University; Borisova.nataliya@gmai.com
[2] Department of Physics, M. V. Lomonosov Moscow State University; harcheva.anastasiya@physics.msu.ru
* Correspondence: Borisova.nataliya@gmai.com

Content

X-Ray data tables 3
Table S1 Crystal data and structure refinements 3
Structure parameters for 2a 4
Table S2 Atomic coordinates and equivalent isotropic displacement parameters (Å) for 2a. U(eq) is defined as one third of the trace of the orthogonalized Uij tensor. 4
Table S3 Bond lengths [Å] and angles [°] for 2a. 4
Table S4 Torsion angles [°] for 2a. 5
Structure parameters for $Sm(3c)(NO_3)_3$ 5
Table S5 Atomic coordinates (x 104) and equivalent isotropic displacement parameters (Å2x 103) for $Sm(3c)(NO_3)_3$. U(eq) is defined as one third of the trace of the orthogonalized Uij tensor. 6
Table S6 Bond lengths [Å] and angles [°] for $Sm(3c)(NO_3)_3$. 8
Table S7 Anisotropic displacement parameters (Å2x 103) for $Sm(3c)(NO_3)_3$. The anisotropic displacement factor exponent takes the form: -2?2[h2 a*2U11 + ... + 2 h k a* b* U12] 12
Table S8 Hydrogen coordinates (x 104) and isotropic displacement parameters (Å2x 10 3) for $Sm(3c)(NO_3)_3$. 14
Table S9 Torsion angles [°] for $Sm(3c)(NO_3)_3$. 14
Structure parameters for $Dy(3c)(NO_3)_3$ – 1st types of crystals 17
Table S10 Atomic coordinates (x 10^4) and equivalent isotropic displacement parameters (Å^{2}x 10^3) for $Dy(3c)(NO_3)_3$ – 1st types of crystals. U(eq) is defined as one third of the trace of the orthogonalized U^{ij} tensor. 17
Table S11 Bond lengths [Å] and angles [°] for $Dy(3c)(NO_3)_3$ – 1st types of crystals. 18

Table S12 Anisotropic displacement parameters ($Å^2$x 10^3) for Dy(3c)$(NO_3)_3$ – 1st types of crystals. The anisotropic displacement factor exponent takes the form: $-2\pi^2$[h^2 $a^{*2}U^{11}$ + ... + 2 h k a* b* U^{12}]........21

Table S13 Hydrogen coordinates (x 10^4) and isotropic displacement parameters ($Å^2$x 10^3) for Dy(3c)$(NO_3)_3$ – 1st types of crystals........22

Table S14 Torsion angles [°] for Dy(3c)$(NO_3)_3$ – 1st types of crystals........23

Structure parameters for Dy(3c)$(NO_3)_3$ – 2nd types of crystals........24

Table S15 Atomic coordinates (x 10^4) and equivalent isotropic displacement parameters ($Å^2$x 10^3) for Dy(3c)$(NO_3)_3$ – 2nd types of crystals. U(eq) is defined as one third of the trace of the orthogonalized U^{ij} tensor........24

Table S16 Bond lengths [Å] and angles [°] for Dy(3c)$(NO_3)_3$ – 2nd types of crystals........25

Table S17 Anisotropic displacement parameters ($Å^2$x 10^3) for Dy(3c)$(NO_3)_3$ – 2nd types of crystals. The anisotropic displacement factor exponent takes the form: $-2\pi^2$[h^2 $a^{*2}U^{11}$ + ... + 2 h k a* b* U^{12}]........28

Table S18 Hydrogen coordinates (x 10^4) and isotropic displacement parameters ($Å^2$x 10^3) for Dy(3c)$(NO_3)_3$ – 2nd types of crystals........29

Table S19. Torsion angles [°] for Dy(3c)$(NO_3)_3$ – 2nd types of crystals........30

UV-vis Titration Data in CH_3CN........31

2c ligand with trivalent lanthanide ions........31

2c with La$(NO_3)_3$·6H_2O........31

2c with Ce$(NO_3)_3$·6H_2O........31

2c with Pr$(NO_3)_3$·6H_2O........32

2c with Nd$(NO_3)_3$·6H_2O........32

2c with Sm$(NO_3)_3$·6H_2O........32

2c with Gd$(NO_3)_3$·6H_2O........33

2c with Er$(NO_3)_3$·5H_2O........33

2c with Tm$(NO_3)_3$·5H_2O........33

2c with Yb$(NO_3)_3$·5H_2O........34

Repeated check of selective result........34

2c with Tm$(NO_3)_3$·5H_2O........34

NMR of Ligands and their REE complexes........35

Fig S1 1H Spectrum of 6-Me-2-Py amide 2c in CDCl3........35

Fig S2 13C Spectrum of 6-Me-2-Py amide 2c in CDCl3........35

Fig S3 2D COSY Spectrum of 6-Me-2-Py ligand 2c in CDCl3........36

Fig S4 2D ROESY Spectrum of 6-Me-2-Py ligand 2c in CDCl3........37

Fig S5 1H Spectrum of 2-Py ligand 2b in CDCl3........38

Fig S6 13C Spectrum of 2-Py ligand 2b in CDCl3........38

Fig 7 1H Spectrum of 4-Py ligand 2d in CDCl3........39

Fig S8 13C Spectrum of 4-Py ligand 2d in CDCl3........39

Fig S9 1H Spectrum of 3c-Sm in acetonitrile-d3..40
Fig S10 2D COSY Spectrum of 3c-Sm in acetonitrile-d3 41
Fig S11 2D NESY Spectrum of 3c-Sm in acetonitrile-d3 42
Fig 12 1H Spectrum of 3c-Eu in acetonitrile-d3 43
Fig S13 13C Spectrum of 3c-Eu in acetonitrile-d3..43
Fig S14 2D COSY Spectrum of 3c-Eu in acetonitrile-d3 44

X-Ray data tables

Table S1 Crystal data and structure refinements.

Identification code	**2a**	Sm(**3c**)(NO_3)$_3$	Dy(**3c**)(NO_3)$_3$ – 1st types of crystals	Dy(**3c**)(NO_3)$_3$ – 2nd types of crystals
Empirical formula	C24 H20 N6 O2	C30 H31 N10 O11 Sm	C30 H31 Dy N10 O11	C30 H33 Dy N10 O12
Formula weight	424.46	858.00	870.15	888.16
Temperature	120(2) K			
Wavelength	0.71073	1.54178 Å	0.71073 Å	0.71073 E
Crystal system	Monoclinic	Monoclinic	Monoclinic	Monoclinic
Space group	P 21/n	$P2_1/c$	$P2_1/c$	$P2_1/c$
Unit cell dimensions	a = 11.6030(9) Å<br>α = 90°<br>b = 6.1039(5) Å<br>β = 106.0700(17)<br>c = 15.0954(12) Å<br>γ = 90°	a = 26.9725(5) Å<br>α = 90°<br>b = 8.07470(10) Å<br>β = 99.5840(10)°<br>c = 16.3105(3) Å<br>γ = 90°	a = 27.033(3) Å<br>α= 90°<br>b = 8.0191(9) Å<br>β= 99.668(3)°<br>c = 16.2653(18) Å<br>γ = 90°	a = 27.1385(14)Å<br>α= 90°<br>b = 8.0371(4) Å<br>β= 99.4800(10)°<br>c = 16.2950(8) Å<br>γ = 90°
Volume	1027.33	3502.76(10) Å3	3475.9(7) Å3	3505.6(3) $Å^3$
Z	2	4	4	4
Density (calculated)	1.372 Mg/m3	1.627 Mg/m3	1.663 Mg/m3	1.683 Mg/m^3
Absorption coefficient	0.092	13.223 mm-1	2.223 mm-1	2.208 mm^{-1}
F(000)	444.0	1724	1740	1780
Crystal size	0.211 x 0.146 x 0.102 mm3	0.38 x 0.08 x 0.07 mm3	0.260 x 0.220 x 0.150 mm3	0.134 x 0.023 x 0.016 mm^3
Theta range for data collection	1.972 to 28.995°	1.66 to 72.00°	2.293 to 27.999°	0.761 to 26.999°.
Index ranges	-15<=h<=15, -7<=k<=8, -16<=l<=20	-31<=h<=33, -9<=k<=9, -19<=l<=19	0<=h<=38, -11<=k<=0, -9<=l<=22	-34<=h<=34, -10<=k<=10, -20<=l<=20
Reflections collected	8135	46719	8382	35532
Independent reflections	2730 [R(int) = 0.0330]	6841 [R(int) = 0.0646]	8382 [R(int) = 0.1161]	7667 [R(int) = 0.0678]
Completeness to theta = 25.242°		99.5 %	99.9 %	100.0 %
Absorption correction	Semi-empirical from equivalents			
Max. and min. transmission	0.991 and 0.981	0.470 and 0.174	0.743 and 0.582	0.979 and 0.742
Refinement method	Full-matrix least-squares on F^2			
Data / restraints /	2730 / 0 / 185	6841 / 0 / 474	8382 / 0 / 474	7667 / 0 / 474

parameters				
Goodness-of-fit on F^2	1.024	1.045	1.011	1.017
Final R indices [I>2sigma(I)]	R1 = 0.0451, wR2 = 0.1065	R1 = 0.0257, wR2 = 0.0605	R1 = 0.0696, wR2 = 0.1085	R1 = 0.0354, wR2 = 0.0758
R indices (all data)	R1 = 0.0647, wR2 = 0.1176	R1 = 0.0289, wR2 = 0.0622	R1 = 0.1270, wR2 = 0.1259	R1 = 0.0574, wR2 = 0.0861
Largest diff. peak and hole	0.370 and -0.239 e.Å-3	0.960 and -0.704 e.Å-3	1.536 and -2.218 e. Å-3	0.860 and -0.811 e. $Å^{-3}$

Structure parameters for 2a

Table S2 Atomic coordinates and equivalent isotropic displacement parameters (Å) for 2a. U(eq) is defined as one third of the trace of the orthogonalized U_{ij} tensor.

	x	y	z	U(eq)
O(1)	0.54389(9)	0.26667(16)	0.19554(7)	0.0232(2)
N(1)	0.48476(10)	0.74899(18)	0.05859(8)	0.0153(2)
N(2)	0.36720(10)	0.4224(2)	0.11188(8)	0.0183(3)
H(2N)	0.3419(16)	0.538(3)	0.0809(12)	0.031(5)
N(3)	0.17091(10)	0.36088(19)	0.10799(8)	0.0211(3)
C(1)	0.48867(12)	0.4120(2)	0.14565(9)	0.0172(3)
C(2)	0.55269(11)	0.6030(2)	0.11647(9)	0.0162(3)
C(3)	0.67669(12)	0.6208(2)	0.15063(10)	0.0197(3)
H(3)	0.7224(14)	0.511(3)	0.1923(11)	0.023(4)
C(4)	0.73217(12)	0.8001(3)	0.12350(10)	0.0217(3)
H(4)	0.8181(14)	0.819(3)	0.1477(10)	0.018(4)
C(5)	0.66367(12)	0.9529(2)	0.06432(9)	0.0184(3)
H(5)	0.6997(15)	1.086(3)	0.0453(12)	0.029(4)
C(6)	0.53926(11)	0.9210(2)	0.03222(9)	0.0152(3)
C(7)	0.28100(12)	0.2739(2)	0.12634(9)	0.0173(3)
C(8)	0.30844(13)	0.0585(2)	0.15465(10)	0.0207(3)
H(8)	0.3884(15)	0.006(3)	0.1682(11)	0.024(4)
C(9)	0.21449(14)	-0.0729(2)	0.16283(10)	0.0236(3)
H(9)	0.2295(15)	-0.229(3)	0.1806(12)	0.032(5)
C(10)	0.10008(14)	0.0143(3)	0.14539(11)	0.0264(3)
H(10)	0.0336(16)	-0.070(3)	0.1527(13)	0.035(5)
C(11)	0.08121(13)	0.2329(2)	0.11919(10)	0.0244(3)
C(12)	-0.03924(15)	0.3409(3)	0.10354(16)	0.0403(5)
H(12C)	-0.099(2)	0.248(4)	0.1210(16)	0.061(7)
H(12B)	-0.069(2)	0.398(4)	0.047(2)	0.079(9)
H(12A)	-0.030(3)	0.479(5)	0.149(2)	0.095(9)

Table S3 Bond lengths [Å] and angles [°] for 2a.

O1 C1 1.2239(16)	N2 C1 1.3611(17)	N3 C7 1.3395(18)
N1 C2 1.3406(17)	N2 C7 1.4108(17)	N3 C11 1.3490(18)
N1 C6 1.3415(17)	N2 H2N 0.854(19)	C1 C2 1.5119(19)

C2 C3 1.3918(18)
C3 C4 1.388(2)
C3 H3 0.970(17)
C4 C5 1.381(2)
C4 H4 0.969(15)
C5 C6 1.4033(17)
C5 H5 0.992(18)
C6 C6 1.489(3) 3_675 ?
C7 C8 1.392(2)
C8 C9 1.386(2)
C8 H8 0.949(17)
C9 C10 1.386(2)
C9 H9 0.995(18)
C10 C11 1.392(2)
C10 H10 0.958(18)
C11 C12 1.504(2)
C12 H12C 0.99(2)
C12 H12B 0.89(3)
C12 H12A 1.08(3)
C2 N1 C6 118.24(11)
C1 N2 C7 128.10(12)
C1 N2 H2N 114.0(12)
C7 N2 H2N 117.8(12)
C7 N3 C11 117.99(12)
O1 C1 N2 125.15(13)
O1 C1 C2 121.50(12)
N2 C1 C2 113.34(11)
N1 C2 C3 123.40(12)
N1 C2 C1 117.05(11)
C3 C2 C1 119.55(12)
C4 C3 C2 117.95(13)
C4 C3 H3 121.4(9)
C2 C3 H3 120.7(9)
C5 C4 C3 119.51(12)
C5 C4 H4 120.9(10)
C3 C4 H4 119.6(9)
C4 C5 C6 118.84(13)
C4 C5 H5 121.8(10)
C6 C5 H5 119.3(10)
N1 C6 C5 122.04(12)
N1 C6 C6 116.59(14) 3_675 ?
C5 C6 C6 121.36(15) 3_675 ?
N3 C7 C8 124.17(12)
N3 C7 N2 112.94(12)
C8 C7 N2 122.87(12)
C9 C8 C7 117.07(13)
C9 C8 H8 122.0(10)
C7 C8 H8 121.0(10)
C10 C9 C8 119.78(14)
C10 C9 H9 120.5(10)
C8 C9 H9 119.7(10)
C9 C10 C11 119.27(13)
C9 C10 H10 122.1(11)
C11 C10 H10 118.6(11)
N3 C11 C10 121.65(14)
N3 C11 C12 116.23(14)
C10 C11 C12 122.10(14)
C11 C12 H12C 113.2(13)
C11 C12 H12B 114.4(17)
H12C C12 H12B 111(2)
C11 C12 H12A 108.4(15)
H12C C12 H12A 103.6(19)
H12B C12 H12A 105(2)

Table S4 Torsion angles [°] for 2a.

C7 N2 C1 O1 0.1(2)
C7 N2 C1 C2 -179.23(12)
C6 N1 C2 C3 0.06(19)
C6 N1 C2 C1 179.46(11)
O1 C1 C2 N1 177.24(12)
N2 C1 C2 N1 -3.41(17)
O1 C1 C2 C3 -3.3(2)
N2 C1 C2 C3 176.02(12)
N1 C2 C3 C4 0.4(2)
C1 C2 C3 C4 -178.99(12)
C2 C3 C4 C5 0.0(2)
C3 C4 C5 C6 -0.8(2)
C2 N1 C6 C5 -0.90(19)
C2 N1 C6 C6 179.87(13)
C4 C5 C6 N1 1.3(2)
C4 C5 C6 C6 -179.54(15)
C11 N3 C7 C8 1.2(2)
C11 N3 C7 N2 179.78(12)
C1 N2 C7 N3 160.89(13)
C1 N2 C7 C8 -20.5(2)
N3 C7 C8 C9 0.9(2)
N2 C7 C8 C9 -177.54(13)
C7 C8 C9 C10 -1.7(2)
C8 C9 C10 C11 0.4(2)
C7 N3 C11 C10 -2.5(2)
C7 N3 C11 C12 176.33(15)
C9 C10 C11 N3 1.7(2)
C9 C10 C11 C12 -177.06(17)

Structure parameters for $Sm(3c)(NO_3)_3$

Table S5 Atomic coordinates (x 10^4) and equivalent isotropic displacement parameters ($Å^2$x 10^3) for Sm(3c)$(NO_3)_3$. U(eq) is defined as one third of the trace of the orthogonalized U_{ij} tensor.

	x	y	z	U(eq)
Sm(1)	2614(1)	9854(1)	10725(1)	17(1)
O(1N)	1681(1)	9930(2)	10282(1)	24(1)
O(2N)	2103(1)	8736(2)	9430(1)	25(1)
O(3N)	1300(1)	9167(4)	9066(2)	59(1)
O(4N)	2231(1)	9080(2)	11948(1)	30(1)
O(5N)	2325(1)	7032(2)	11139(1)	25(1)
O(6N)	1981(1)	6627(2)	12244(1)	29(1)
O(7N)	3396(1)	11611(3)	10968(1)	32(1)
O(8N)	3267(1)	10223(2)	12045(1)	32(1)
O(9N)	3894(1)	11978(3)	12150(2)	48(1)
N(1N)	1682(1)	9263(3)	9576(1)	26(1)
N(2N)	2170(1)	7537(2)	11790(1)	21(1)
N(3N)	3532(1)	11286(3)	11737(2)	28(1)
O(1)	3196(1)	7693(2)	10522(1)	27(1)
O(2)	2289(1)	12330(2)	11318(1)	20(1)
N(1)	3034(1)	9972(2)	9368(1)	21(1)
N(2)	2403(1)	12297(2)	9735(1)	18(1)
N(3)	3816(1)	6463(3)	9991(2)	26(1)
N(4)	4414(1)	7960(3)	9406(2)	29(1)
N(5)	1542(1)	13665(2)	11004(1)	20(1)
N(6)	986(1)	15825(3)	10530(1)	24(1)
C(1)	3285(1)	8629(3)	9184(2)	23(1)
C(2)	3347(1)	8239(3)	8376(2)	25(1)
C(3)	3164(1)	9341(3)	7744(2)	25(1)
C(4)	2925(1)	10780(3)	7938(2)	24(1)
C(5)	2861(1)	11038(3)	8758(2)	21(1)
C(6)	2120(1)	13506(3)	9978(2)	19(1)
C(7)	2010(1)	14957(3)	9534(2)	22(1)
C(8)	2194(1)	15150(3)	8795(2)	25(1)
C(9)	2473(1)	13884(3)	8521(2)	24(1)
C(10)	2572(1)	12467(3)	9009(2)	21(1)
C(11)	3439(1)	7567(3)	9938(2)	23(1)
C(12)	3914(1)	5407(3)	10746(2)	32(1)
C(13)	4176(1)	6372(4)	11499(2)	38(1)
C(14)	4186(1)	6512(3)	9453(2)	27(1)
C(15)	4270(1)	5108(4)	9014(2)	34(1)
C(16)	4619(1)	5229(4)	8484(2)	38(1)
C(17)	4864(1)	6709(4)	8422(2)	36(1)
C(18)	4752(1)	8063(4)	8883(2)	34(1)
C(19)	4994(2)	9720(5)	8820(3)	54(1)
C(20)	1978(1)	13139(3)	10816(1)	18(1)
C(21)	1427(1)	13376(3)	11851(2)	23(1)
C(22)	1138(1)	11782(4)	11901(2)	36(1)
C(23)	1140(1)	14293(3)	10389(2)	22(1)
C(24)	940(1)	13309(3)	9723(2)	26(1)

C(25)	567(1)	13999(4)	9135(2)	30(1)
C(26)	403(1)	15594(4)	9253(2)	30(1)
C(27)	616(1)	16470(3)	9960(2)	27(1)
C(28)	444(1)	18190(3)	10128(2)	34(1)
N(1S)	256(1)	9868(4)	7778(2)	57(1)
C(1S)	490(1)	8782(4)	7629(2)	35(1)
C(2S)	786(1)	7382(4)	7431(2)	33(1)

Table S6 Bond lengths [Å] and angles [°] for Sm(3c)(NO_3)$_3$.

Sm(1)-O(1)	2.4048(17)	C(7)-C(8)	1.386(4)
Sm(1)-O(2)	2.4470(16)	C(7)-H(7A)	0.9500
Sm(1)-O(4N)	2.4745(18)	C(8)-C(9)	1.388(4)
Sm(1)-O(2N)	2.4922(18)	C(8)-H(8A)	0.9500
Sm(1)-O(1N)	2.5011(18)	C(9)-C(10)	1.393(3)
Sm(1)-O(7N)	2.5168(19)	C(9)-H(9A)	0.9500
Sm(1)-O(5N)	2.5364(17)	C(12)-C(13)	1.526(5)
Sm(1)-N(2)	2.5528(19)	C(12)-H(12A)	0.9900
Sm(1)-O(8N)	2.561(2)	C(12)-H(12B)	0.9900
Sm(1)-N(1)	2.649(2)	C(13)-H(13A)	0.9800
Sm(1)-N(1N)	2.915(2)	C(13)-H(13B)	0.9800
Sm(1)-N(2N)	2.941(2)	C(13)-H(13C)	0.9800
O(1N)-N(1N)	1.271(3)	C(14)-C(15)	1.380(4)
O(2N)-N(1N)	1.272(3)	C(15)-C(16)	1.383(4)
O(3N)-N(1N)	1.213(3)	C(15)-H(15A)	0.9500
O(4N)-N(2N)	1.277(3)	C(16)-C(17)	1.376(5)
O(5N)-N(2N)	1.271(3)	C(16)-H(16A)	0.9500
O(6N)-N(2N)	1.215(3)	C(17)-C(18)	1.388(4)
O(7N)-N(3N)	1.274(3)	C(17)-H(17A)	0.9500
O(8N)-N(3N)	1.272(3)	C(18)-C(19)	1.499(4)
O(9N)-N(3N)	1.226(3)	C(19)-H(19A)	0.9800
O(1)-C(11)	1.246(3)	C(19)-H(19B)	0.9800
O(2)-C(20)	1.254(3)	C(19)-H(19C)	0.9800
N(1)-C(1)	1.338(3)	C(21)-C(22)	1.515(3)
N(1)-C(5)	1.340(3)	C(21)-H(21A)	0.9900
N(2)-C(6)	1.339(3)	C(21)-H(21B)	0.9900
N(2)-C(10)	1.345(3)	C(22)-H(22A)	0.9800
N(3)-C(11)	1.345(3)	C(22)-H(22B)	0.9800
N(3)-C(14)	1.436(3)	C(22)-H(22C)	0.9800
N(3)-C(12)	1.484(3)	C(23)-C(24)	1.381(4)
N(4)-C(14)	1.328(4)	C(24)-C(25)	1.387(4)
N(4)-C(18)	1.351(4)	C(24)-H(24A)	0.9500
N(5)-C(20)	1.333(3)	C(25)-C(26)	1.386(4)
N(5)-C(23)	1.442(3)	C(25)-H(25A)	0.9500
N(5)-C(21)	1.483(3)	C(26)-C(27)	1.393(4)
N(6)-C(23)	1.337(3)	C(26)-H(26A)	0.9500
N(6)-C(27)	1.350(4)	C(27)-C(28)	1.503(4)
C(1)-C(2)	1.393(4)	C(28)-H(28A)	0.9800
C(1)-C(11)	1.500(4)	C(28)-H(28B)	0.9800
C(2)-C(3)	1.388(4)	C(28)-H(28C)	0.9800
C(2)-H(2A)	0.9500	N(1S)-C(1S)	1.131(5)
C(3)-C(4)	1.391(4)	C(1S)-C(2S)	1.450(4)
C(3)-H(3A)	0.9500	C(2S)-H(2SA)	0.9800
C(4)-C(5)	1.392(4)	C(2S)-H(2SB)	0.9800
C(4)-H(4A)	0.9500	C(2S)-H(2SC)	0.9800
C(5)-C(10)	1.487(3)		
C(6)-C(7)	1.384(3)	O(1)-Sm(1)-O(2)	158.77(6)
C(6)-C(20)	1.509(3)	O(1)-Sm(1)-O(4N)	107.28(6)

O(2)-Sm(1)-O(4N)	70.26(6)	N(1)-Sm(1)-N(1N)	84.95(6)
O(1)-Sm(1)-O(2N)	84.40(6)	O(1)-Sm(1)-N(2N)	87.65(6)
O(2)-Sm(1)-O(2N)	116.52(6)	O(2)-Sm(1)-N(2N)	94.47(5)
O(4N)-Sm(1)-O(2N)	110.17(6)	O(4N)-Sm(1)-N(2N)	25.47(6)
O(1)-Sm(1)-O(1N)	128.74(6)	O(2N)-Sm(1)-N(2N)	92.72(6)
O(2)-Sm(1)-O(1N)	71.58(5)	O(1N)-Sm(1)-N(2N)	72.32(6)
O(4N)-Sm(1)-O(1N)	72.74(7)	O(7N)-Sm(1)-N(2N)	132.74(6)
O(2N)-Sm(1)-O(1N)	51.35(6)	O(5N)-Sm(1)-N(2N)	25.48(6)
O(1)-Sm(1)-O(7N)	83.27(7)	N(2)-Sm(1)-N(2N)	142.68(6)
O(2)-Sm(1)-O(7N)	79.80(6)	O(8N)-Sm(1)-N(2N)	82.42(6)
O(4N)-Sm(1)-O(7N)	117.81(7)	N(1)-Sm(1)-N(2N)	142.09(6)
O(2N)-Sm(1)-O(7N)	131.99(6)	N(1N)-Sm(1)-N(2N)	83.24(6)
O(1N)-Sm(1)-O(7N)	143.88(6)	N(1N)-O(1N)-Sm(1)	95.66(14)
O(1)-Sm(1)-O(5N)	67.76(6)	N(1N)-O(2N)-Sm(1)	96.06(14)
O(2)-Sm(1)-O(5N)	118.83(5)	N(2N)-O(4N)-Sm(1)	98.12(13)
O(4N)-Sm(1)-O(5N)	50.94(6)	N(2N)-O(5N)-Sm(1)	95.34(13)
O(2N)-Sm(1)-O(5N)	75.46(6)	N(3N)-O(7N)-Sm(1)	97.76(14)
O(1N)-Sm(1)-O(5N)	76.08(6)	N(3N)-O(8N)-Sm(1)	95.70(15)
O(7N)-Sm(1)-O(5N)	138.69(6)	O(3N)-N(1N)-O(1N)	121.4(2)
O(1)-Sm(1)-N(2)	123.74(6)	O(3N)-N(1N)-O(2N)	122.1(2)
O(2)-Sm(1)-N(2)	64.01(6)	O(1N)-N(1N)-O(2N)	116.5(2)
O(4N)-Sm(1)-N(2)	128.75(6)	O(3N)-N(1N)-Sm(1)	173.7(2)
O(2N)-Sm(1)-N(2)	73.15(6)	O(1N)-N(1N)-Sm(1)	58.62(12)
O(1N)-Sm(1)-N(2)	72.00(6)	O(2N)-N(1N)-Sm(1)	58.22(12)
O(7N)-Sm(1)-N(2)	75.92(6)	O(6N)-N(2N)-O(5N)	123.1(2)
O(5N)-Sm(1)-N(2)	144.76(6)	O(6N)-N(2N)-O(4N)	121.3(2)
O(1)-Sm(1)-O(8N)	80.10(7)	O(5N)-N(2N)-O(4N)	115.57(19)
O(2)-Sm(1)-O(8N)	79.26(6)	O(6N)-N(2N)-Sm(1)	177.70(17)
O(4N)-Sm(1)-O(8N)	70.88(7)	O(5N)-N(2N)-Sm(1)	59.18(11)
O(2N)-Sm(1)-O(8N)	163.91(6)	O(4N)-N(2N)-Sm(1)	56.41(11)
O(1N)-Sm(1)-O(8N)	139.21(6)	O(9N)-N(3N)-O(8N)	122.6(3)
O(7N)-Sm(1)-O(8N)	50.34(6)	O(9N)-N(3N)-O(7N)	121.3(2)
O(5N)-Sm(1)-O(8N)	94.55(6)	O(8N)-N(3N)-O(7N)	116.0(2)
N(2)-Sm(1)-O(8N)	119.43(6)	O(9N)-N(3N)-Sm(1)	175.7(2)
O(1)-Sm(1)-N(1)	62.13(6)	O(8N)-N(3N)-Sm(1)	59.07(13)
O(2)-Sm(1)-N(1)	122.14(6)	O(7N)-N(3N)-Sm(1)	57.09(12)
O(4N)-Sm(1)-N(1)	167.42(6)	C(11)-O(1)-Sm(1)	127.03(16)
O(2N)-Sm(1)-N(1)	63.78(6)	C(20)-O(2)-Sm(1)	114.35(14)
O(1N)-Sm(1)-N(1)	107.89(6)	C(1)-N(1)-C(5)	118.7(2)
O(7N)-Sm(1)-N(1)	69.44(7)	C(1)-N(1)-Sm(1)	117.41(16)
O(5N)-Sm(1)-N(1)	116.61(6)	C(5)-N(1)-Sm(1)	119.98(15)
N(2)-Sm(1)-N(1)	61.65(6)	C(6)-N(2)-C(10)	118.6(2)
O(8N)-Sm(1)-N(1)	111.67(7)	C(6)-N(2)-Sm(1)	117.01(15)
O(1)-Sm(1)-N(1N)	107.80(7)	C(10)-N(2)-Sm(1)	124.32(15)
O(2)-Sm(1)-N(1N)	93.42(6)	C(11)-N(3)-C(14)	122.6(2)
O(4N)-Sm(1)-N(1N)	92.57(7)	C(11)-N(3)-C(12)	117.6(2)
O(2N)-Sm(1)-N(1N)	25.72(6)	C(14)-N(3)-C(12)	118.4(2)
O(1N)-Sm(1)-N(1N)	25.72(6)	C(14)-N(4)-C(18)	117.3(2)
O(7N)-Sm(1)-N(1N)	143.52(6)	C(20)-N(5)-C(23)	122.8(2)
O(5N)-Sm(1)-N(1N)	75.72(6)	C(20)-N(5)-C(21)	119.6(2)
N(2)-Sm(1)-N(1N)	69.04(6)	C(23)-N(5)-C(21)	116.97(19)
O(8N)-Sm(1)-N(1N)	163.31(6)	C(23)-N(6)-C(27)	117.0(2)

N(1)-C(1)-C(2)	122.7(2)	C(17)-C(16)-H(16A)	120.3
N(1)-C(1)-C(11)	111.1(2)	C(15)-C(16)-H(16A)	120.3
C(2)-C(1)-C(11)	125.9(2)	C(16)-C(17)-C(18)	119.6(3)
C(3)-C(2)-C(1)	118.2(2)	C(16)-C(17)-H(17A)	120.2
C(3)-C(2)-H(2A)	120.9	C(18)-C(17)-H(17A)	120.2
C(1)-C(2)-H(2A)	120.9	N(4)-C(18)-C(17)	121.5(3)
C(2)-C(3)-C(4)	119.3(2)	N(4)-C(18)-C(19)	116.4(3)
C(2)-C(3)-H(3A)	120.4	C(17)-C(18)-C(19)	122.0(3)
C(4)-C(3)-H(3A)	120.4	C(18)-C(19)-H(19A)	109.5
C(3)-C(4)-C(5)	118.7(2)	C(18)-C(19)-H(19B)	109.5
C(3)-C(4)-H(4A)	120.7	H(19A)-C(19)-H(19B)	109.5
C(5)-C(4)-H(4A)	120.7	C(18)-C(19)-H(19C)	109.5
N(1)-C(5)-C(4)	122.3(2)	H(19A)-C(19)-H(19C)	109.5
N(1)-C(5)-C(10)	115.3(2)	H(19B)-C(19)-H(19C)	109.5
C(4)-C(5)-C(10)	122.4(2)	O(2)-C(20)-N(5)	122.3(2)
N(2)-C(6)-C(7)	123.1(2)	O(2)-C(20)-C(6)	116.8(2)
N(2)-C(6)-C(20)	111.6(2)	N(5)-C(20)-C(6)	120.9(2)
C(7)-C(6)-C(20)	125.1(2)	N(5)-C(21)-C(22)	111.9(2)
C(6)-C(7)-C(8)	118.2(2)	N(5)-C(21)-H(21A)	109.2
C(6)-C(7)-H(7A)	120.9	C(22)-C(21)-H(21A)	109.2
C(8)-C(7)-H(7A)	120.9	N(5)-C(21)-H(21B)	109.2
C(7)-C(8)-C(9)	119.3(2)	C(22)-C(21)-H(21B)	109.2
C(7)-C(8)-H(8A)	120.3	H(21A)-C(21)-H(21B)	107.9
C(9)-C(8)-H(8A)	120.3	C(21)-C(22)-H(22A)	109.5
C(8)-C(9)-C(10)	118.9(2)	C(21)-C(22)-H(22B)	109.5
C(8)-C(9)-H(9A)	120.6	H(22A)-C(22)-H(22B)	109.5
C(10)-C(9)-H(9A)	120.6	C(21)-C(22)-H(22C)	109.5
N(2)-C(10)-C(9)	121.8(2)	H(22A)-C(22)-H(22C)	109.5
N(2)-C(10)-C(5)	115.6(2)	H(22B)-C(22)-H(22C)	109.5
C(9)-C(10)-C(5)	122.6(2)	N(6)-C(23)-C(24)	125.2(2)
O(1)-C(11)-N(3)	119.6(2)	N(6)-C(23)-N(5)	115.2(2)
O(1)-C(11)-C(1)	118.1(2)	C(24)-C(23)-N(5)	119.6(2)
N(3)-C(11)-C(1)	122.3(2)	C(23)-C(24)-C(25)	117.2(2)
N(3)-C(12)-C(13)	111.6(2)	C(23)-C(24)-H(24A)	121.4
N(3)-C(12)-H(12A)	109.3	C(25)-C(24)-H(24A)	121.4
C(13)-C(12)-H(12A)	109.3	C(26)-C(25)-C(24)	119.3(3)
N(3)-C(12)-H(12B)	109.3	C(26)-C(25)-H(25A)	120.4
C(13)-C(12)-H(12B)	109.3	C(24)-C(25)-H(25A)	120.4
H(12A)-C(12)-H(12B)	108.0	C(25)-C(26)-C(27)	119.3(2)
C(12)-C(13)-H(13A)	109.5	C(25)-C(26)-H(26A)	120.3
C(12)-C(13)-H(13B)	109.5	C(27)-C(26)-H(26A)	120.3
H(13A)-C(13)-H(13B)	109.5	N(6)-C(27)-C(26)	122.0(2)
C(12)-C(13)-H(13C)	109.5	N(6)-C(27)-C(28)	116.5(3)
H(13A)-C(13)-H(13C)	109.5	C(26)-C(27)-C(28)	121.5(3)
H(13B)-C(13)-H(13C)	109.5	C(27)-C(28)-H(28A)	109.5
N(4)-C(14)-C(15)	125.1(3)	C(27)-C(28)-H(28B)	109.5
N(4)-C(14)-N(3)	115.8(2)	H(28A)-C(28)-H(28B)	109.5
C(15)-C(14)-N(3)	119.1(3)	C(27)-C(28)-H(28C)	109.5
C(14)-C(15)-C(16)	117.0(3)	H(28A)-C(28)-H(28C)	109.5
C(14)-C(15)-H(15A)	121.5	H(28B)-C(28)-H(28C)	109.5
C(16)-C(15)-H(15A)	121.5	N(1S)-C(1S)-C(2S)	179.3(4)
C(17)-C(16)-C(15)	119.5(3)	C(1S)-C(2S)-H(2SA)	109.5

C(1S)-C(2S)-H(2SB)	109.5	H(2SA)-C(2S)-H(2SC)	109.5
H(2SA)-C(2S)-H(2SB)	109.5	H(2SB)-C(2S)-H(2SC)	109.5
C(1S)-C(2S)-H(2SC)	109.5		

Table S7 Anisotropic displacement parameters ($Å^2 x 10^3$) for $Sm(3c)(NO_3)_3$. The anisotropic displacement factor exponent takes the form: $-2\pi^2[h^2 a^{*2}U_{11} + ... + 2 h k a^* b^* U_{12}]$

	U_{11}	U_{22}	U_{33}	U_{23}	U_{13}	U_{12}
Sm(1)	22(1)	15(1)	14(1)	1(1)	4(1)	2(1)
O(1N)	27(1)	22(1)	22(1)	-4(1)	5(1)	-2(1)
O(2N)	33(1)	20(1)	22(1)	-3(1)	6(1)	1(1)
O(3N)	30(1)	104(2)	38(1)	-29(1)	-6(1)	-8(1)
O(4N)	51(1)	19(1)	23(1)	-4(1)	14(1)	-7(1)
O(5N)	34(1)	20(1)	21(1)	-2(1)	8(1)	1(1)
O(6N)	36(1)	25(1)	28(1)	4(1)	10(1)	-5(1)
O(7N)	32(1)	38(1)	25(1)	4(1)	1(1)	-6(1)
O(8N)	33(1)	38(1)	23(1)	6(1)	2(1)	1(1)
O(9N)	34(1)	62(2)	42(1)	-4(1)	-7(1)	-9(1)
N(1N)	28(1)	27(1)	23(1)	-4(1)	4(1)	-7(1)
N(2N)	24(1)	19(1)	21(1)	2(1)	2(1)	0(1)
N(3N)	23(1)	34(1)	27(1)	-1(1)	-2(1)	2(1)
O(1)	32(1)	24(1)	26(1)	5(1)	10(1)	9(1)
O(2)	27(1)	16(1)	15(1)	0(1)	2(1)	1(1)
N(1)	23(1)	20(1)	20(1)	0(1)	5(1)	2(1)
N(2)	21(1)	17(1)	16(1)	0(1)	2(1)	-1(1)
N(3)	28(1)	22(1)	30(1)	4(1)	9(1)	7(1)
N(4)	29(1)	34(1)	25(1)	-3(1)	6(1)	0(1)
N(5)	25(1)	17(1)	18(1)	0(1)	5(1)	2(1)
N(6)	26(1)	21(1)	24(1)	3(1)	7(1)	3(1)
C(1)	21(1)	22(1)	25(1)	0(1)	5(1)	3(1)
C(2)	24(1)	24(1)	26(1)	-4(1)	6(1)	4(1)
C(3)	25(1)	33(1)	19(1)	-6(1)	7(1)	2(1)
C(4)	24(1)	27(1)	19(1)	1(1)	3(1)	3(1)
C(5)	21(1)	20(1)	21(1)	1(1)	5(1)	1(1)
C(6)	22(1)	17(1)	17(1)	-2(1)	3(1)	-2(1)
C(7)	31(1)	17(1)	17(1)	-1(1)	6(1)	1(1)
C(8)	37(1)	19(1)	20(1)	4(1)	8(1)	2(1)
C(9)	35(1)	22(1)	18(1)	3(1)	8(1)	2(1)
C(10)	24(1)	21(1)	18(1)	2(1)	4(1)	1(1)
C(11)	25(1)	18(1)	26(1)	-1(1)	6(1)	3(1)
C(12)	35(1)	25(1)	38(2)	10(1)	10(1)	9(1)
C(13)	35(1)	44(2)	35(2)	10(1)	6(1)	9(1)
C(14)	23(1)	31(1)	26(1)	1(1)	4(1)	7(1)
C(15)	31(1)	32(1)	40(2)	-3(1)	8(1)	7(1)
C(16)	34(1)	43(2)	39(2)	-8(1)	9(1)	13(1)
C(17)	28(1)	53(2)	29(2)	-3(1)	9(1)	4(1)
C(18)	31(1)	46(2)	26(2)	-3(1)	5(1)	-5(1)
C(19)	67(2)	59(2)	44(2)	-13(2)	27(2)	-28(2)
C(20)	26(1)	12(1)	15(1)	-3(1)	3(1)	-2(1)
C(21)	30(1)	22(1)	20(1)	1(1)	9(1)	2(1)
C(22)	46(2)	30(1)	36(2)	2(1)	18(1)	-8(1)
C(23)	23(1)	22(1)	22(1)	2(1)	7(1)	2(1)
C(24)	27(1)	23(1)	27(1)	-1(1)	5(1)	0(1)

C(25)	26(1)	37(1)	26(1)	-2(1)	3(1)	-2(1)
C(26)	24(1)	37(1)	29(2)	8(1)	4(1)	4(1)
C(27)	25(1)	28(1)	28(1)	7(1)	8(1)	4(1)
C(28)	34(1)	31(1)	37(2)	7(1)	8(1)	12(1)
N(1S)	46(2)	67(2)	56(2)	3(2)	-3(1)	21(2)
C(1S)	30(1)	46(2)	27(2)	4(1)	-3(1)	-4(1)
C(2S)	35(1)	34(1)	30(2)	-5(1)	7(1)	-10(1)

Table S8 Hydrogen coordinates (x 10^4) and isotropic displacement parameters ($Å^2$x 10^3) for Sm(3c)(NO_3)$_3$.

	x	y	z	U(eq)
H(2A)	3511	7245	8259	30
H(3A)	3201	9114	7186	30
H(4A)	2808	11572	7520	28
H(7A)	1813	15798	9730	26
H(8A)	2130	16140	8480	30
H(9A)	2595	13981	8009	29
H(12A)	4127	4457	10641	39
H(12B)	3591	4963	10866	39
H(13A)	4246	5631	11980	57
H(13B)	3959	7279	11622	57
H(13C)	4494	6827	11379	57
H(15A)	4096	4103	9072	41
H(16A)	4690	4299	8167	46
H(17A)	5107	6803	8065	44
H(19A)	4732	10562	8672	82
H(19B)	5208	9679	8391	82
H(19C)	5198	10003	9356	82
H(21A)	1227	14316	12009	28
H(21B)	1746	13330	12253	28
H(22A)	1066	11641	12466	54
H(22B)	1340	10845	11762	54
H(22C)	822	11826	11506	54
H(24A)	1053	12206	9671	31
H(25A)	426	13386	8655	36
H(26A)	147	16085	8856	36
H(28A)	736	18871	10352	51
H(28B)	214	18139	10532	51
H(28C)	271	18681	9609	51
H(2SA)	943	7651	6947	39
H(2SB)	567	6415	7305	39
H(2SC)	1047	7133	7908	39

Table S9 Torsion angles [°] for Sm(3c)(NO_3)$_3$.

O(1)-Sm(1)-O(1N)-N(1N)	-40.28(16)
O(2)-Sm(1)-O(1N)-N(1N)	146.75(14)
O(4N)-Sm(1)-O(1N)-N(1N)	-138.87(14)
O(2N)-Sm(1)-O(1N)-N(1N)	-3.57(13)
O(7N)-Sm(1)-O(1N)-N(1N)	107.36(15)
O(5N)-Sm(1)-O(1N)-N(1N)	-85.94(14)
N(2)-Sm(1)-O(1N)-N(1N)	78.93(14)
O(8N)-Sm(1)-O(1N)-N(1N)	-166.60(13)
N(1)-Sm(1)-O(1N)-N(1N)	27.95(14)
N(2N)-Sm(1)-O(1N)-N(1N)	-112.15(14)
O(1)-Sm(1)-O(2N)-N(1N)	155.65(14)
O(2)-Sm(1)-O(2N)-N(1N)	-28.09(15)
O(4N)-Sm(1)-O(2N)-N(1N)	49.27(15)
O(1N)-Sm(1)-O(2N)-N(1N)	3.57(13)
O(7N)-Sm(1)-O(2N)-N(1N)	-128.63(14)
O(5N)-Sm(1)-O(2N)-N(1N)	87.21(14)
N(2)-Sm(1)-O(2N)-N(1N)	-76.57(14)
O(8N)-Sm(1)-O(2N)-N(1N)	140.1(2)

N(1)-Sm(1)-O(2N)-N(1N)	-142.74(15)
N(2N)-Sm(1)-O(2N)-N(1N)	68.28(14)
O(1)-Sm(1)-O(4N)-N(2N)	-41.16(16)
O(2)-Sm(1)-O(4N)-N(2N)	161.09(16)
O(2N)-Sm(1)-O(4N)-N(2N)	49.15(16)
O(1N)-Sm(1)-O(4N)-N(2N)	84.98(15)
O(7N)-Sm(1)-O(4N)-N(2N)	-132.61(14)
O(5N)-Sm(1)-O(4N)-N(2N)	-0.87(13)
N(2)-Sm(1)-O(4N)-N(2N)	133.35(14)
O(8N)-Sm(1)-O(4N)-N(2N)	-113.79(16)
N(1)-Sm(1)-O(4N)-N(2N)	-9.9(4)
N(1N)-Sm(1)-O(4N)-N(2N)	68.38(15)
O(1)-Sm(1)-O(5N)-N(2N)	139.03(15)
O(2)-Sm(1)-O(5N)-N(2N)	-18.57(15)
O(4N)-Sm(1)-O(5N)-N(2N)	0.87(13)
O(2N)-Sm(1)-O(5N)-N(2N)	-131.14(14)
O(1N)-Sm(1)-O(5N)-N(2N)	-78.04(13)
O(7N)-Sm(1)-O(5N)-N(2N)	90.11(15)
N(2)-Sm(1)-O(5N)-N(2N)	-103.52(15)
O(8N)-Sm(1)-O(5N)-N(2N)	61.67(14)
N(1)-Sm(1)-O(5N)-N(2N)	178.68(13)
N(1N)-Sm(1)-O(5N)-N(2N)	-104.56(14)
O(1)-Sm(1)-O(7N)-N(3N)	-85.02(15)
O(2)-Sm(1)-O(7N)-N(3N)	82.13(15)
O(4N)-Sm(1)-O(7N)-N(3N)	21.00(17)
O(2N)-Sm(1)-O(7N)-N(3N)	-161.23(13)
O(1N)-Sm(1)-O(7N)-N(3N)	119.84(15)
O(5N)-Sm(1)-O(7N)-N(3N)	-40.38(19)
N(2)-Sm(1)-O(7N)-N(3N)	147.67(16)
O(8N)-Sm(1)-O(7N)-N(3N)	-2.32(13)
N(1)-Sm(1)-O(7N)-N(3N)	-147.72(16)
N(1N)-Sm(1)-O(7N)-N(3N)	164.00(13)
N(2N)-Sm(1)-O(7N)-N(3N)	-4.53(19)
O(1)-Sm(1)-O(8N)-N(3N)	91.76(15)
O(2)-Sm(1)-O(8N)-N(3N)	-83.27(14)
O(4N)-Sm(1)-O(8N)-N(3N)	-155.93(16)
O(2N)-Sm(1)-O(8N)-N(3N)	107.5(2)
O(1N)-Sm(1)-O(8N)-N(3N)	-127.87(14)
O(7N)-Sm(1)-O(8N)-N(3N)	2.32(13)
O(5N)-Sm(1)-O(8N)-N(3N)	158.22(14)
N(2)-Sm(1)-O(8N)-N(3N)	-31.52(16)
N(1)-Sm(1)-O(8N)-N(3N)	37.22(16)
N(1N)-Sm(1)-O(8N)-N(3N)	-148.4(2)
N(2N)-Sm(1)-O(8N)-N(3N)	-179.32(15)
Sm(1)-O(1N)-N(1N)-O(3N)	-172.6(3)
Sm(1)-O(1N)-N(1N)-O(2N)	6.1(2)
Sm(1)-O(2N)-N(1N)-O(3N)	172.5(3)
Sm(1)-O(2N)-N(1N)-O(1N)	-6.1(2)
O(1)-Sm(1)-N(1N)-O(1N)	148.02(13)
O(2)-Sm(1)-N(1N)-O(1N)	-31.41(14)
O(4N)-Sm(1)-N(1N)-O(1N)	38.96(14)
O(2N)-Sm(1)-N(1N)-O(1N)	173.6(2)
O(7N)-Sm(1)-N(1N)-O(1N)	-108.84(15)
O(5N)-Sm(1)-N(1N)-O(1N)	87.49(13)
N(2)-Sm(1)-N(1N)-O(1N)	-91.87(14)
O(8N)-Sm(1)-N(1N)-O(1N)	31.8(3)
N(1)-Sm(1)-N(1N)-O(1N)	-153.40(14)
N(2N)-Sm(1)-N(1N)-O(1N)	62.70(13)
O(1)-Sm(1)-N(1N)-O(2N)	-25.53(15)
O(2)-Sm(1)-N(1N)-O(2N)	155.03(14)
O(4N)-Sm(1)-N(1N)-O(2N)	-134.60(14)
O(1N)-Sm(1)-N(1N)-O(2N)	-173.6(2)
O(7N)-Sm(1)-N(1N)-O(2N)	77.60(17)
O(5N)-Sm(1)-N(1N)-O(2N)	-86.07(14)
N(2)-Sm(1)-N(1N)-O(2N)	94.57(14)
O(8N)-Sm(1)-N(1N)-O(2N)	-141.7(2)
N(1)-Sm(1)-N(1N)-O(2N)	33.04(14)
N(2N)-Sm(1)-N(1N)-O(2N)	-110.86(14)
Sm(1)-O(5N)-N(2N)-O(6N)	179.5(2)
Sm(1)-O(5N)-N(2N)-O(4N)	-1.4(2)
Sm(1)-O(4N)-N(2N)-O(6N)	-179.43(19)
Sm(1)-O(4N)-N(2N)-O(5N)	1.5(2)
O(1)-Sm(1)-N(2N)-O(5N)	-37.41(14)
O(2)-Sm(1)-N(2N)-O(5N)	163.75(14)
O(4N)-Sm(1)-N(2N)-O(5N)	-178.4(2)
O(2N)-Sm(1)-N(2N)-O(5N)	46.87(14)
O(1N)-Sm(1)-N(2N)-O(5N)	94.70(14)
O(7N)-Sm(1)-N(2N)-O(5N)	-116.01(14)
N(2)-Sm(1)-N(2N)-O(5N)	112.25(15)
O(8N)-Sm(1)-N(2N)-O(5N)	-117.72(14)
N(1)-Sm(1)-N(2N)-O(5N)	-1.92(18)
N(1N)-Sm(1)-N(2N)-O(5N)	70.83(14)
O(1)-Sm(1)-N(2N)-O(4N)	141.03(15)
O(2)-Sm(1)-N(2N)-O(4N)	-17.82(15)
O(2N)-Sm(1)-N(2N)-O(4N)	-134.70(15)
O(1N)-Sm(1)-N(2N)-O(4N)	-86.87(15)
O(7N)-Sm(1)-N(2N)-O(4N)	62.42(17)
O(5N)-Sm(1)-N(2N)-O(4N)	178.4(2)
N(2)-Sm(1)-N(2N)-O(4N)	-69.32(18)
O(8N)-Sm(1)-N(2N)-O(4N)	60.71(15)
N(1)-Sm(1)-N(2N)-O(4N)	176.51(14)
N(1N)-Sm(1)-N(2N)-O(4N)	-110.74(15)
Sm(1)-O(8N)-N(3N)-O(9N)	175.4(2)
Sm(1)-O(8N)-N(3N)-O(7N)	-3.9(2)
Sm(1)-O(7N)-N(3N)-O(9N)	-175.3(2)
Sm(1)-O(7N)-N(3N)-O(8N)	4.0(2)
O(1)-Sm(1)-N(3N)-O(8N)	-84.16(15)
O(2)-Sm(1)-N(3N)-O(8N)	91.45(15)
O(4N)-Sm(1)-N(3N)-O(8N)	22.73(15)
O(2N)-Sm(1)-N(3N)-O(8N)	-141.66(16)
O(1N)-Sm(1)-N(3N)-O(8N)	85.55(18)
O(7N)-Sm(1)-N(3N)-O(8N)	-175.8(2)
O(5N)-Sm(1)-N(3N)-O(8N)	-24.71(16)
N(2)-Sm(1)-N(3N)-O(8N)	152.67(14)

N(1)-Sm(1)-N(3N)-O(8N)	-145.79(15)
N(1N)-Sm(1)-N(3N)-O(8N)	141.2(2)
N(2N)-Sm(1)-N(3N)-O(8N)	0.71(16)
O(1)-Sm(1)-N(3N)-O(7N)	91.64(15)
O(2)-Sm(1)-N(3N)-O(7N)	-92.75(15)
O(4N)-Sm(1)-N(3N)-O(7N)	-161.47(15)
O(2N)-Sm(1)-N(3N)-O(7N)	34.1(2)
O(1N)-Sm(1)-N(3N)-O(7N)	-98.65(18)
O(5N)-Sm(1)-N(3N)-O(7N)	151.09(14)
N(2)-Sm(1)-N(3N)-O(7N)	-31.53(16)
O(8N)-Sm(1)-N(3N)-O(7N)	175.8(2)
N(1)-Sm(1)-N(3N)-O(7N)	30.01(15)
N(1N)-Sm(1)-N(3N)-O(7N)	-43.0(3)
N(2N)-Sm(1)-N(3N)-O(7N)	176.51(14)
O(2)-Sm(1)-O(1)-C(11)	-105.3(2)
O(4N)-Sm(1)-O(1)-C(11)	174.8(2)
O(2N)-Sm(1)-O(1)-C(11)	65.4(2)
O(1N)-Sm(1)-O(1)-C(11)	93.4(2)
O(7N)-Sm(1)-O(1)-C(11)	-68.1(2)
O(5N)-Sm(1)-O(1)-C(11)	142.0(2)
N(2)-Sm(1)-O(1)-C(11)	0.0(2)
O(8N)-Sm(1)-O(1)-C(11)	-118.9(2)
N(1)-Sm(1)-O(1)-C(11)	2.2(2)
N(1N)-Sm(1)-O(1)-C(11)	76.3(2)
N(2N)-Sm(1)-O(1)-C(11)	158.4(2)
O(1)-Sm(1)-O(2)-C(20)	149.45(17)
O(4N)-Sm(1)-O(2)-C(20)	-123.55(16)
O(2N)-Sm(1)-O(2)-C(20)	-20.22(16)
O(1N)-Sm(1)-O(2)-C(20)	-45.83(15)
O(7N)-Sm(1)-O(2)-C(20)	111.83(15)
O(5N)-Sm(1)-O(2)-C(20)	-107.61(15)
N(2)-Sm(1)-O(2)-C(20)	32.63(15)
O(8N)-Sm(1)-O(2)-C(20)	163.09(16)
N(1)-Sm(1)-O(2)-C(20)	54.14(17)
N(1N)-Sm(1)-O(2)-C(20)	-32.04(15)
N(2N)-Sm(1)-O(2)-C(20)	-115.51(15)
O(1)-Sm(1)-N(1)-C(1)	11.47(17)
O(2)-Sm(1)-N(1)-C(1)	167.40(16)
O(4N)-Sm(1)-N(1)-C(1)	-22.6(4)
O(2N)-Sm(1)-N(1)-C(1)	-86.43(18)
O(1N)-Sm(1)-N(1)-C(1)	-113.51(18)
O(7N)-Sm(1)-N(1)-C(1)	104.73(19)
O(5N)-Sm(1)-N(1)-C(1)	-30.5(2)
N(2)-Sm(1)-N(1)-C(1)	-170.6(2)
O(8N)-Sm(1)-N(1)-C(1)	76.66(19)
N(1N)-Sm(1)-N(1)-C(1)	-101.73(18)
N(2N)-Sm(1)-N(1)-C(1)	-29.5(2)
O(1)-Sm(1)-N(1)-C(5)	169.0(2)
O(2)-Sm(1)-N(1)-C(5)	-35.0(2)
O(4N)-Sm(1)-N(1)-C(5)	134.9(3)
O(2N)-Sm(1)-N(1)-C(5)	71.14(18)
O(1N)-Sm(1)-N(1)-C(5)	44.06(19)

O(7N)-Sm(1)-N(1)-C(5)	-97.70(18)
O(5N)-Sm(1)-N(1)-C(5)	127.11(17)
N(2)-Sm(1)-N(1)-C(5)	-13.03(17)
O(8N)-Sm(1)-N(1)-C(5)	-125.76(18)
N(1N)-Sm(1)-N(1)-C(5)	55.85(18)
N(2N)-Sm(1)-N(1)-C(5)	128.03(17)
O(1)-Sm(1)-N(2)-C(6)	-172.20(15)
O(2)-Sm(1)-N(2)-C(6)	-15.06(15)
O(4N)-Sm(1)-N(2)-C(6)	14.11(19)
O(2N)-Sm(1)-N(2)-C(6)	116.76(17)
O(1N)-Sm(1)-N(2)-C(6)	62.75(16)
O(7N)-Sm(1)-N(2)-C(6)	-100.43(17)
O(5N)-Sm(1)-N(2)-C(6)	88.80(18)
O(8N)-Sm(1)-N(2)-C(6)	-74.19(17)
N(1)-Sm(1)-N(2)-C(6)	-174.40(18)
N(1N)-Sm(1)-N(2)-C(6)	89.88(17)
N(2N)-Sm(1)-N(2)-C(6)	45.2(2)
O(1)-Sm(1)-N(2)-C(10)	5.3(2)
O(2)-Sm(1)-N(2)-C(10)	162.5(2)
O(4N)-Sm(1)-N(2)-C(10)	-168.36(17)
O(2N)-Sm(1)-N(2)-C(10)	-65.71(18)
O(1N)-Sm(1)-N(2)-C(10)	-119.72(19)
O(7N)-Sm(1)-N(2)-C(10)	77.10(18)
O(5N)-Sm(1)-N(2)-C(10)	-93.7(2)
O(8N)-Sm(1)-N(2)-C(10)	103.33(18)
N(1)-Sm(1)-N(2)-C(10)	3.12(17)
N(1N)-Sm(1)-N(2)-C(10)	-92.59(19)
N(2N)-Sm(1)-N(2)-C(10)	-137.30(17)
C(5)-N(1)-C(1)-C(2)	-4.2(4)
Sm(1)-N(1)-C(1)-C(2)	153.64(19)
C(5)-N(1)-C(1)-C(11)	-178.8(2)
Sm(1)-N(1)-C(1)-C(11)	-20.9(3)
N(1)-C(1)-C(2)-C(3)	3.7(4)
C(11)-C(1)-C(2)-C(3)	177.4(2)
C(1)-C(2)-C(3)-C(4)	-0.2(4)
C(2)-C(3)-C(4)-C(5)	-2.4(4)
C(1)-N(1)-C(5)-C(4)	1.3(4)
Sm(1)-N(1)-C(5)-C(4)	-155.94(18)
C(1)-N(1)-C(5)-C(10)	178.2(2)
Sm(1)-N(1)-C(5)-C(10)	20.9(3)
C(3)-C(4)-C(5)-N(1)	2.0(4)
C(3)-C(4)-C(5)-C(10)	-174.7(2)
C(10)-N(2)-C(6)-C(7)	-2.8(3)
Sm(1)-N(2)-C(6)-C(7)	174.92(18)
C(10)-N(2)-C(6)-C(20)	-178.2(2)
Sm(1)-N(2)-C(6)-C(20)	-0.5(2)
N(2)-C(6)-C(7)-C(8)	1.2(4)
C(20)-C(6)-C(7)-C(8)	176.0(2)
C(6)-C(7)-C(8)-C(9)	1.1(4)
C(7)-C(8)-C(9)-C(10)	-1.7(4)
C(6)-N(2)-C(10)-C(9)	2.1(3)
Sm(1)-N(2)-C(10)-C(9)	-175.41(18)

C(6)-N(2)-C(10)-C(5)	-176.9(2)	C(14)-N(4)-C(18)-C(17)	-0.9(4)
Sm(1)-N(2)-C(10)-C(5)	5.6(3)	C(14)-N(4)-C(18)-C(19)	178.3(3)
C(8)-C(9)-C(10)-N(2)	0.1(4)	C(16)-C(17)-C(18)-N(4)	1.2(5)
C(8)-C(9)-C(10)-C(5)	179.0(2)	C(16)-C(17)-C(18)-C(19)	-178.0(3)
N(1)-C(5)-C(10)-N(2)	-17.3(3)	Sm(1)-O(2)-C(20)-N(5)	132.38(19)
C(4)-C(5)-C(10)-N(2)	159.5(2)	Sm(1)-O(2)-C(20)-C(6)	-47.9(2)
N(1)-C(5)-C(10)-C(9)	163.8(2)	C(23)-N(5)-C(20)-O(2)	-167.1(2)
C(4)-C(5)-C(10)-C(9)	-19.4(4)	C(21)-N(5)-C(20)-O(2)	3.6(3)
Sm(1)-O(1)-C(11)-N(3)	167.69(17)	C(23)-N(5)-C(20)-C(6)	13.2(3)
Sm(1)-O(1)-C(11)-C(1)	-14.2(3)	C(21)-N(5)-C(20)-C(6)	-176.2(2)
C(14)-N(3)-C(11)-O(1)	-164.6(2)	N(2)-C(6)-C(20)-O(2)	31.9(3)
C(12)-N(3)-C(11)-O(1)	1.7(4)	C(7)-C(6)-C(20)-O(2)	-143.4(2)
C(14)-N(3)-C(11)-C(1)	17.4(4)	N(2)-C(6)-C(20)-N(5)	-148.3(2)
C(12)-N(3)-C(11)-C(1)	-176.3(2)	C(7)-C(6)-C(20)-N(5)	36.3(3)
N(1)-C(1)-C(11)-O(1)	23.0(3)	C(20)-N(5)-C(21)-C(22)	-92.8(3)
C(2)-C(1)-C(11)-O(1)	-151.3(2)	C(23)-N(5)-C(21)-C(22)	78.4(3)
N(1)-C(1)-C(11)-N(3)	-158.9(2)	C(27)-N(6)-C(23)-C(24)	-2.2(4)
C(2)-C(1)-C(11)-N(3)	26.7(4)	C(27)-N(6)-C(23)-N(5)	178.8(2)
C(11)-N(3)-C(12)-C(13)	-75.1(3)	C(20)-N(5)-C(23)-N(6)	-123.1(2)
C(14)-N(3)-C(12)-C(13)	91.8(3)	C(21)-N(5)-C(23)-N(6)	66.1(3)
C(18)-N(4)-C(14)-C(15)	0.4(4)	C(20)-N(5)-C(23)-C(24)	57.9(3)
C(18)-N(4)-C(14)-N(3)	-177.8(2)	C(21)-N(5)-C(23)-C(24)	-113.0(2)
C(11)-N(3)-C(14)-N(4)	51.8(4)	N(6)-C(23)-C(24)-C(25)	3.4(4)
C(12)-N(3)-C(14)-N(4)	-114.3(3)	N(5)-C(23)-C(24)-C(25)	-177.7(2)
C(11)-N(3)-C(14)-C(15)	-126.4(3)	C(23)-C(24)-C(25)-C(26)	-2.1(4)
C(12)-N(3)-C(14)-C(15)	67.4(4)	C(24)-C(25)-C(26)-C(27)	0.1(4)
N(4)-C(14)-C(15)-C(16)	0.0(5)	C(23)-N(6)-C(27)-C(26)	-0.1(4)
N(3)-C(14)-C(15)-C(16)	178.1(3)	C(23)-N(6)-C(27)-C(28)	179.7(2)
C(14)-C(15)-C(16)-C(17)	0.2(5)	C(25)-C(26)-C(27)-N(6)	1.2(4)
C(15)-C(16)-C(17)-C(18)	-0.7(5)	C(25)-C(26)-C(27)-C(28)	-178.7(2)

Structure parameters for $Dy(3c)(NO_3)_3$ – 1st types of crystals

Table S10 Atomic coordinates (x 10^4) and equivalent isotropic displacement parameters ($Å^2$x 10^3) for $Dy(3c)(NO_3)_3$ – 1st types of crystals. U(eq) is defined as one third of the trace of the orthogonalized U^{ij} tensor.

	x	y	z	U(eq)
Dy(1)	2384(1)	-111(1)	4306(1)	15(1)
O(1)	2707(2)	2326(5)	3711(3)	15(1)
O(2)	1795(2)	-2205(6)	4431(3)	20(1)
N(1)	2597(2)	2269(7)	5292(4)	16(1)
N(2)	3460(2)	3643(7)	4013(3)	17(1)
N(3)	4019(2)	5804(7)	4495(4)	17(1)
N(4)	1964(2)	-40(8)	5632(3)	17(1)
N(5)	1184(2)	-3533(7)	4955(4)	21(1)
N(6)	581(2)	-2125(8)	5564(4)	26(2)
C(1)	2430(3)	2441(8)	6023(4)	16(2)
C(2)	2539(3)	3841(9)	6521(5)	21(2)
C(3)	2825(3)	5125(9)	6259(4)	21(1)
C(4)	3004(2)	4935(9)	5515(4)	18(1)

C(5)	2886(3)	3484(8)	5056(4)	14(1)
C(6)	3021(3)	3128(8)	4208(4)	15(2)
C(7)	3573(3)	3380(9)	3175(4)	20(2)
C(8)	3850(3)	1765(9)	3104(5)	31(2)
C(9)	3857(3)	4264(9)	4639(5)	17(2)
C(10)	4062(3)	3271(9)	5298(4)	21(2)
C(11)	4439(3)	3962(10)	5880(5)	26(2)
C(12)	4602(3)	5560(9)	5769(5)	24(2)
C(13)	4389(3)	6461(9)	5062(5)	23(2)
C(14)	4559(3)	8190(9)	4904(5)	28(2)
C(15)	2141(3)	994(8)	6260(4)	15(1)
C(16)	2074(3)	687(9)	7073(4)	17(2)
C(17)	1834(3)	-775(9)	7246(4)	20(2)
C(18)	1649(3)	-1837(9)	6605(4)	21(2)
C(19)	1711(3)	-1410(8)	5797(4)	17(2)
C(20)	1553(3)	-2401(9)	5017(5)	21(2)
C(21)	1080(3)	-4548(9)	4173(5)	32(2)
C(22)	821(3)	-3528(11)	3448(5)	34(2)
C(23)	815(3)	-3549(9)	5496(5)	22(2)
C(24)	743(3)	-4994(11)	5918(5)	33(2)
C(25)	395(3)	-4909(12)	6451(5)	37(2)
C(26)	151(3)	-3461(11)	6539(5)	30(2)
C(27)	245(3)	-2055(11)	6091(5)	31(2)
C(28)	-6(4)	-406(12)	6177(6)	53(3)
O(1N)	2869(2)	-1294(6)	5577(3)	20(1)
O(2N)	3298(2)	-93(6)	4729(3)	22(1)
O(3N)	3682(2)	-1039(8)	5925(4)	44(2)
O(4N)	1628(2)	1652(6)	4072(3)	26(1)
O(5N)	1755(2)	240(6)	3000(3)	27(1)
O(6N)	1122(2)	1981(8)	2887(4)	40(2)
O(7N)	2674(2)	-2927(6)	3927(3)	22(1)
O(8N)	2753(2)	-901(6)	3097(3)	26(1)
O(9N)	3018(2)	-3353(6)	2812(3)	25(1)
N(1N)	3299(2)	-816(7)	5434(4)	19(1)
N(2N)	1492(3)	1314(8)	3308(4)	27(2)
N(3N)	2825(2)	-2434(7)	3267(4)	19(1)
N(1S)	4724(3)	5082(11)	2197(5)	50(2)
C(1S)	4498(3)	6188(11)	2376(5)	28(2)
C(2S)	4221(3)	7604(10)	2596(5)	32(2)

Table S11 Bond lengths [Å] and angles [°] for Dy(3c)$(NO_3)_3$ – 1st types of crystals.

Dy(1)-O(2)	2.346(5)	Dy(1)-N(3N)	2.902(6)
Dy(1)-O(1)	2.407(5)	O(1)-C(6)	1.248(8)
Dy(1)-O(8N)	2.433(5)	O(2)-C(20)	1.253(8)
Dy(1)-O(1N)	2.446(5)	N(1)-C(5)	1.345(8)
Dy(1)-O(2N)	2.450(4)	N(1)-C(1)	1.348(8)
Dy(1)-O(4N)	2.461(5)	N(2)-C(6)	1.345(9)
Dy(1)-N(1)	2.497(6)	N(2)-C(9)	1.439(9)
Dy(1)-O(7N)	2.501(5)	N(2)-C(7)	1.462(8)
Dy(1)-O(5N)	2.503(5)	N(3)-C(9)	1.343(8)
Dy(1)-N(4)	2.603(5)	N(3)-C(13)	1.349(9)
Dy(1)-N(1N)	2.877(6)	N(4)-C(15)	1.340(8)

N(4)-C(19)	1.345(8)	C(28)-H(28B)	0.9800
N(5)-C(20)	1.341(9)	C(28)-H(28C)	0.9800
N(5)-C(23)	1.436(9)	O(1N)-N(1N)	1.281(7)
N(5)-C(21)	1.496(9)	O(2N)-N(1N)	1.285(7)
N(6)-C(23)	1.320(10)	O(3N)-N(1N)	1.210(8)
N(6)-C(27)	1.351(10)	O(4N)-N(2N)	1.266(8)
C(1)-C(2)	1.387(9)	O(5N)-N(2N)	1.272(8)
C(1)-C(15)	1.485(9)	O(6N)-N(2N)	1.234(8)
C(2)-C(3)	1.396(9)	O(7N)-N(3N)	1.273(7)
C(2)-H(2A)	0.9500	O(8N)-N(3N)	1.268(7)
C(3)-C(4)	1.386(9)	O(9N)-N(3N)	1.222(7)
C(3)-H(3A)	0.9500	N(1S)-C(1S)	1.143(10)
C(4)-C(5)	1.390(9)	C(1S)-C(2S)	1.436(11)
C(4)-H(4A)	0.9500	C(2S)-H(2SC)	0.9800
C(5)-C(6)	1.513(9)	C(2S)-H(2SA)	0.9800
C(7)-C(8)	1.509(10)	C(2S)-H(2SB)	0.9800
C(7)-H(7A)	0.9900		
C(7)-H(7B)	0.9900	O(2)-Dy(1)-O(1)	155.86(17)
C(8)-H(8A)	0.9800	O(2)-Dy(1)-O(8N)	105.11(16)
C(8)-H(8B)	0.9800	O(1)-Dy(1)-O(8N)	70.20(16)
C(8)-H(8C)	0.9800	O(2)-Dy(1)-O(1N)	85.62(17)
C(9)-C(10)	1.374(10)	O(1)-Dy(1)-O(1N)	118.42(16)
C(10)-C(11)	1.384(10)	O(8N)-Dy(1)-O(1N)	110.34(17)
C(10)-H(10A)	0.9500	O(2)-Dy(1)-O(2N)	130.27(17)
C(11)-C(12)	1.376(10)	O(1)-Dy(1)-O(2N)	72.07(16)
C(11)-H(11A)	0.9500	O(8N)-Dy(1)-O(2N)	72.39(17)
C(12)-C(13)	1.398(10)	O(1N)-Dy(1)-O(2N)	52.64(16)
C(12)-H(12A)	0.9500	O(2)-Dy(1)-O(4N)	82.32(18)
C(13)-C(14)	1.496(10)	O(1)-Dy(1)-O(4N)	79.59(17)
C(14)-H(14A)	0.9800	O(8N)-Dy(1)-O(4N)	117.89(17)
C(14)-H(14B)	0.9800	O(1N)-Dy(1)-O(4N)	131.77(17)
C(14)-H(14C)	0.9800	O(2N)-Dy(1)-O(4N)	144.26(17)
C(15)-C(16)	1.387(9)	O(2)-Dy(1)-N(1)	125.26(17)
C(16)-C(17)	1.391(9)	O(1)-Dy(1)-N(1)	65.05(17)
C(16)-H(16A)	0.9500	O(8N)-Dy(1)-N(1)	129.53(18)
C(17)-C(18)	1.375(10)	O(1N)-Dy(1)-N(1)	73.77(17)
C(17)-H(17A)	0.9500	O(2N)-Dy(1)-N(1)	72.69(17)
C(18)-C(19)	1.395(9)	O(4N)-Dy(1)-N(1)	75.87(18)
C(18)-H(18A)	0.9500	O(2)-Dy(1)-O(7N)	67.70(17)
C(19)-C(20)	1.497(10)	O(1)-Dy(1)-O(7N)	118.96(17)
C(21)-C(22)	1.508(11)	O(8N)-Dy(1)-O(7N)	51.52(16)
C(21)-H(21A)	0.9900	O(1N)-Dy(1)-O(7N)	73.30(16)
C(21)-H(21B)	0.9900	O(2N)-Dy(1)-O(7N)	74.45(16)
C(22)-H(22A)	0.9800	O(4N)-Dy(1)-O(7N)	140.06(17)
C(22)-H(22B)	0.9800	N(1)-Dy(1)-O(7N)	143.18(17)
C(22)-H(22C)	0.9800	O(2)-Dy(1)-O(5N)	77.31(17)
C(23)-C(24)	1.378(10)	O(1)-Dy(1)-O(5N)	78.93(16)
C(24)-C(25)	1.384(11)	O(8N)-Dy(1)-O(5N)	69.90(18)
C(24)-H(24A)	0.9500	O(1N)-Dy(1)-O(5N)	162.14(16)
C(25)-C(26)	1.354(12)	O(2N)-Dy(1)-O(5N)	138.30(16)
C(25)-H(25A)	0.9500	O(4N)-Dy(1)-O(5N)	51.43(17)
C(26)-C(27)	1.389(11)	N(1)-Dy(1)-O(5N)	120.62(18)
C(26)-H(26A)	0.9500	O(7N)-Dy(1)-O(5N)	95.08(17)
C(27)-C(28)	1.503(12)	O(2)-Dy(1)-N(4)	62.99(17)
C(28)-H(28A)	0.9800	O(1)-Dy(1)-N(4)	123.62(18)

O(8N)-Dy(1)-N(4)	166.13(19)	C(5)-C(4)-H(4A)	120.7
O(1N)-Dy(1)-N(4)	63.53(16)	N(1)-C(5)-C(4)	123.0(6)
O(2N)-Dy(1)-N(4)	109.09(15)	N(1)-C(5)-C(6)	111.3(6)
O(4N)-Dy(1)-N(4)	69.42(17)	C(4)-C(5)-C(6)	125.4(6)
N(1)-Dy(1)-N(4)	62.39(19)	O(1)-C(6)-N(2)	122.5(6)
O(7N)-Dy(1)-N(4)	114.98(18)	O(1)-C(6)-C(5)	116.5(6)
O(5N)-Dy(1)-N(4)	111.83(16)	N(2)-C(6)-C(5)	120.9(6)
O(2)-Dy(1)-N(1N)	109.06(17)	N(2)-C(7)-C(8)	112.2(6)
O(1)-Dy(1)-N(1N)	94.92(17)	N(2)-C(7)-H(7A)	109.2
O(8N)-Dy(1)-N(1N)	92.23(17)	C(8)-C(7)-H(7A)	109.2
O(1N)-Dy(1)-N(1N)	26.29(16)	N(2)-C(7)-H(7B)	109.2
O(2N)-Dy(1)-N(1N)	26.39(15)	C(8)-C(7)-H(7B)	109.2
O(4N)-Dy(1)-N(1N)	144.46(16)	H(7A)-C(7)-H(7B)	107.9
N(1)-Dy(1)-N(1N)	70.12(17)	C(7)-C(8)-H(8A)	109.5
O(7N)-Dy(1)-N(1N)	73.06(16)	C(7)-C(8)-H(8B)	109.5
O(5N)-Dy(1)-N(1N)	162.12(17)	H(8A)-C(8)-H(8B)	109.5
N(4)-Dy(1)-N(1N)	85.62(16)	C(7)-C(8)-H(8C)	109.5
O(2)-Dy(1)-N(3N)	86.70(17)	H(8A)-C(8)-H(8C)	109.5
O(1)-Dy(1)-N(3N)	94.31(17)	H(8B)-C(8)-H(8C)	109.5
O(8N)-Dy(1)-N(3N)	25.61(16)	N(3)-C(9)-C(10)	124.7(7)
O(1N)-Dy(1)-N(3N)	91.65(16)	N(3)-C(9)-N(2)	114.9(7)
O(2N)-Dy(1)-N(3N)	70.97(16)	C(10)-C(9)-N(2)	120.4(6)
O(4N)-Dy(1)-N(3N)	133.68(16)	C(9)-C(10)-C(11)	117.1(7)
N(1)-Dy(1)-N(3N)	142.34(17)	C(9)-C(10)-H(10A)	121.5
O(7N)-Dy(1)-N(3N)	25.92(15)	C(11)-C(10)-H(10A)	121.5
O(5N)-Dy(1)-N(3N)	82.26(17)	C(12)-C(11)-C(10)	119.9(8)
N(4)-Dy(1)-N(3N)	140.87(18)	C(12)-C(11)-H(11A)	120.1
N(1N)-Dy(1)-N(3N)	81.49(16)	C(10)-C(11)-H(11A)	120.1
C(6)-O(1)-Dy(1)	114.3(4)	C(11)-C(12)-C(13)	119.5(7)
C(20)-O(2)-Dy(1)	127.2(4)	C(11)-C(12)-H(12A)	120.2
C(5)-N(1)-C(1)	118.3(6)	C(13)-C(12)-H(12A)	120.2
C(5)-N(1)-Dy(1)	116.9(4)	N(3)-C(13)-C(12)	121.0(7)
C(1)-N(1)-Dy(1)	124.7(5)	N(3)-C(13)-C(14)	117.3(7)
C(6)-N(2)-C(9)	121.5(6)	C(12)-C(13)-C(14)	121.7(7)
C(6)-N(2)-C(7)	120.4(6)	C(13)-C(14)-H(14A)	109.5
C(9)-N(2)-C(7)	117.6(6)	C(13)-C(14)-H(14B)	109.5
C(9)-N(3)-C(13)	117.7(7)	H(14A)-C(14)-H(14B)	109.5
C(15)-N(4)-C(19)	118.8(6)	C(13)-C(14)-H(14C)	109.5
C(15)-N(4)-Dy(1)	119.7(4)	H(14A)-C(14)-H(14C)	109.5
C(19)-N(4)-Dy(1)	117.1(4)	H(14B)-C(14)-H(14C)	109.5
C(20)-N(5)-C(23)	123.0(6)	N(4)-C(15)-C(16)	122.0(6)
C(20)-N(5)-C(21)	117.6(6)	N(4)-C(15)-C(1)	115.0(6)
C(23)-N(5)-C(21)	117.8(6)	C(16)-C(15)-C(1)	123.0(6)
C(23)-N(6)-C(27)	118.2(7)	C(15)-C(16)-C(17)	118.9(7)
N(1)-C(1)-C(2)	121.8(6)	C(15)-C(16)-H(16A)	120.5
N(1)-C(1)-C(15)	114.7(6)	C(17)-C(16)-H(16A)	120.5
C(2)-C(1)-C(15)	123.4(6)	C(18)-C(17)-C(16)	119.4(7)
C(1)-C(2)-C(3)	119.5(7)	C(18)-C(17)-H(17A)	120.3
C(1)-C(2)-H(2A)	120.2	C(16)-C(17)-H(17A)	120.3
C(3)-C(2)-H(2A)	120.2	C(17)-C(18)-C(19)	118.5(7)
C(4)-C(3)-C(2)	118.6(7)	C(17)-C(18)-H(18A)	120.7
C(4)-C(3)-H(3A)	120.7	C(19)-C(18)-H(18A)	120.7
C(2)-C(3)-H(3A)	120.7	N(4)-C(19)-C(18)	122.3(6)
C(3)-C(4)-C(5)	118.6(6)	N(4)-C(19)-C(20)	110.2(6)
C(3)-C(4)-H(4A)	120.7	C(18)-C(19)-C(20)	127.3(6)

O(2)-C(20)-N(5)	120.4(7)
O(2)-C(20)-C(19)	117.8(6)
N(5)-C(20)-C(19)	121.7(6)
N(5)-C(21)-C(22)	111.4(6)
N(5)-C(21)-H(21A)	109.3
C(22)-C(21)-H(21A)	109.3
N(5)-C(21)-H(21B)	109.3
C(22)-C(21)-H(21B)	109.3
H(21A)-C(21)-H(21B)	108.0
C(21)-C(22)-H(22A)	109.5
C(21)-C(22)-H(22B)	109.5
H(22A)-C(22)-H(22B)	109.5
C(21)-C(22)-H(22C)	109.5
H(22A)-C(22)-H(22C)	109.5
H(22B)-C(22)-H(22C)	109.5
N(6)-C(23)-C(24)	125.1(7)
N(6)-C(23)-N(5)	115.9(6)
C(24)-C(23)-N(5)	119.0(7)
C(23)-C(24)-C(25)	116.1(8)
C(23)-C(24)-H(24A)	122.0
C(25)-C(24)-H(24A)	122.0
C(26)-C(25)-C(24)	120.1(8)
C(26)-C(25)-H(25A)	119.9
C(24)-C(25)-H(25A)	119.9
C(25)-C(26)-C(27)	120.4(8)
C(25)-C(26)-H(26A)	119.8
C(27)-C(26)-H(26A)	119.8
N(6)-C(27)-C(26)	120.0(8)
N(6)-C(27)-C(28)	117.2(8)
C(26)-C(27)-C(28)	122.8(8)
C(27)-C(28)-H(28A)	109.5
C(27)-C(28)-H(28B)	109.5
H(28A)-C(28)-H(28B)	109.5
C(27)-C(28)-H(28C)	109.5
H(28A)-C(28)-H(28C)	109.5
H(28B)-C(28)-H(28C)	109.5
N(1N)-O(1N)-Dy(1)	96.0(4)
N(1N)-O(2N)-Dy(1)	95.7(4)
N(2N)-O(4N)-Dy(1)	97.2(4)
N(2N)-O(5N)-Dy(1)	95.0(4)
N(3N)-O(7N)-Dy(1)	94.9(4)
N(3N)-O(8N)-Dy(1)	98.4(4)
O(3N)-N(1N)-O(1N)	122.5(6)
O(3N)-N(1N)-O(2N)	121.9(6)
O(1N)-N(1N)-O(2N)	115.5(6)
O(3N)-N(1N)-Dy(1)	176.9(5)
O(1N)-N(1N)-Dy(1)	57.8(3)
O(2N)-N(1N)-Dy(1)	57.9(3)
O(6N)-N(2N)-O(4N)	121.8(7)
O(6N)-N(2N)-O(5N)	122.0(7)
O(4N)-N(2N)-O(5N)	116.2(6)
O(6N)-N(2N)-Dy(1)	177.4(5)
O(4N)-N(2N)-Dy(1)	57.2(4)
O(5N)-N(2N)-Dy(1)	59.1(3)
O(9N)-N(3N)-O(8N)	121.3(6)
O(9N)-N(3N)-O(7N)	123.6(6)
O(8N)-N(3N)-O(7N)	115.2(6)
O(9N)-N(3N)-Dy(1)	177.2(5)
O(8N)-N(3N)-Dy(1)	56.0(3)
O(7N)-N(3N)-Dy(1)	59.2(3)
N(1S)-C(1S)-C(2S)	178.7(9)
C(1S)-C(2S)-H(2SC)	109.5
C(1S)-C(2S)-H(2SA)	109.5
H(2SC)-C(2S)-H(2SA)	109.5
C(1S)-C(2S)-H(2SB)	109.5
H(2SC)-C(2S)-H(2SB)	109.5
H(2SA)-C(2S)-H(2SB)	109.5

Table S12 Anisotropic displacement parameters (Å^{2}x 10^3) for Dy(3c)(NO$_3$)$_3$ – 1st types of crystals. The anisotropic displacement factor exponent takes the form: $-2\pi^2$[h^2 $a^{*2}U^{11}$ + ... + 2 h k a* b* U^{12}]

	U^{11}	U^{22}	U^{33}	U^{23}	U^{13}	U^{12}
Dy(1)	19(1)	14(1)	12(1)	-1(1)	4(1)	-2(1)
O(1)	23(3)	15(2)	8(2)	-1(2)	2(2)	-1(2)
O(2)	23(3)	22(3)	19(3)	-6(2)	9(2)	-8(2)
N(1)	16(3)	15(3)	17(3)	3(2)	1(2)	-2(2)
N(2)	25(3)	14(3)	14(3)	3(2)	8(3)	-1(2)
N(3)	22(4)	18(3)	13(3)	1(2)	6(3)	-6(3)
N(4)	21(3)	19(3)	14(3)	0(3)	7(2)	2(3)
N(5)	21(3)	24(3)	19(3)	-3(3)	6(3)	-8(3)
N(6)	26(4)	38(4)	15(3)	3(3)	6(3)	-3(3)
C(1)	17(4)	22(4)	10(3)	2(3)	6(3)	2(3)
C(2)	27(4)	22(4)	17(4)	-5(3)	8(3)	-5(3)
C(3)	30(4)	12(3)	21(3)	-4(3)	6(3)	-8(3)
C(4)	26(3)	15(3)	14(3)	0(3)	10(3)	-1(4)

C(5)	16(4)	14(3)	10(3)	0(3)	0(3)	2(3)
C(6)	24(4)	9(3)	13(3)	3(3)	2(3)	6(3)
C(7)	24(4)	26(4)	11(3)	0(3)	9(3)	-3(3)
C(8)	43(5)	29(4)	26(4)	-7(3)	21(4)	-1(4)
C(9)	9(4)	23(3)	21(4)	-2(3)	10(3)	-2(3)
C(10)	26(4)	20(4)	18(4)	-1(3)	6(3)	-2(3)
C(11)	18(4)	37(5)	22(4)	5(4)	4(3)	6(4)
C(12)	17(4)	28(4)	26(4)	-2(3)	3(3)	5(3)
C(13)	17(4)	27(4)	27(4)	-8(3)	11(3)	-3(3)
C(14)	36(5)	24(4)	25(4)	-6(3)	5(4)	-11(4)
C(15)	14(4)	21(3)	11(3)	1(3)	2(3)	5(3)
C(16)	15(4)	23(3)	13(4)	-4(3)	2(3)	-4(3)
C(17)	15(4)	33(4)	13(4)	7(3)	7(3)	-8(3)
C(18)	15(4)	27(4)	21(4)	1(3)	3(3)	-4(3)
C(19)	14(4)	22(4)	16(4)	-1(3)	3(3)	-4(3)
C(20)	27(4)	18(4)	19(4)	3(3)	5(3)	1(3)
C(21)	32(5)	29(4)	35(5)	-7(3)	8(4)	-9(3)
C(22)	21(4)	48(5)	32(5)	-2(4)	5(4)	-13(4)
C(23)	22(4)	25(4)	21(4)	0(3)	4(3)	-10(3)
C(24)	31(4)	30(4)	39(4)	8(5)	8(3)	-4(5)
C(25)	34(4)	46(5)	33(4)	14(5)	7(3)	-19(5)
C(26)	22(4)	49(5)	19(4)	2(4)	6(3)	-8(4)
C(27)	25(5)	53(5)	15(4)	3(4)	6(3)	6(4)
C(28)	57(6)	71(7)	38(5)	18(5)	25(5)	27(6)
O(1N)	27(3)	18(2)	18(3)	1(2)	6(2)	1(2)
O(2N)	24(2)	19(2)	22(2)	0(3)	4(2)	2(3)
O(3N)	21(3)	80(5)	26(3)	16(3)	-6(3)	4(3)
O(4N)	29(3)	31(3)	18(3)	-6(2)	3(2)	2(2)
O(5N)	32(3)	31(3)	17(2)	-2(2)	0(2)	-5(3)
O(6N)	27(3)	56(4)	34(4)	-1(3)	-6(3)	4(3)
O(7N)	26(3)	19(3)	22(3)	4(2)	7(2)	-3(2)
O(8N)	42(3)	13(2)	24(3)	0(2)	13(3)	4(2)
O(9N)	28(3)	27(3)	21(3)	-2(2)	9(2)	9(2)
N(1N)	29(4)	20(3)	9(3)	-5(2)	6(3)	6(3)
N(2N)	24(4)	32(4)	27(4)	4(3)	6(3)	-2(3)
N(3N)	19(3)	26(3)	9(3)	1(2)	1(3)	0(3)
N(1S)	43(4)	55(5)	47(5)	-1(5)	-3(4)	8(5)
C(1S)	31(5)	37(5)	18(4)	4(4)	4(4)	-4(4)
C(2S)	41(5)	39(5)	18(4)	-5(4)	7(4)	-5(4)

Table S13 Hydrogen coordinates (x 10^4) and isotropic displacement parameters (Å^{2}x 10^3) for Dy(3c)$(NO_3)_3$ – 1st types of crystals.

	x	y	z	U(eq)
H(2A)	2420	3926	7037	25
H(3A)	2895	6108	6584	25
H(4A)	3203	5779	5322	21
H(7A)	3780	4319	3029	23
H(7B)	3256	3371	2770	23
H(8A)	3920	1644	2535	47
H(8B)	3643	828	3232	47
H(8C)	4166	1773	3499	47

H(10A)	3950	2160	5352	25
H(11A)	4585	3335	6355	31
H(12A)	4856	6047	6171	29
H(14A)	4270	8856	4646	42
H(14B)	4807	8144	4529	42
H(14C)	4711	8703	5433	42
H(16A)	2190	1461	7504	21
H(17A)	1799	-1036	7803	24
H(18A)	1483	-2840	6710	25
H(21A)	866	-5511	4262	38
H(21B)	1400	-4987	4042	38
H(22A)	743	-4240	2954	50
H(22B)	1043	-2620	3334	50
H(22C)	510	-3059	3584	50
H(24A)	922	-5990	5848	40
H(25A)	326	-5869	6756	45
H(26A)	-84	-3406	6910	36
H(28A)	247	480	6251	80
H(28B)	-177	-437	6662	80
H(28C)	-251	-185	5673	80
H(2SC)	4450	8548	2735	48
H(2SA)	4064	7326	3079	48
H(2SB)	3961	7903	2124	48

Table S14 Torsion angles [°] for Dy(3c)(NO_3)$_3$ – 1st types of crystals.

C(5)-N(1)-C(1)-C(2)	1.5(10)
Dy(1)-N(1)-C(1)-C(2)	-176.3(5)
C(5)-N(1)-C(1)-C(15)	-176.2(6)
Dy(1)-N(1)-C(1)-C(15)	6.0(8)
N(1)-C(1)-C(2)-C(3)	0.8(11)
C(15)-C(1)-C(2)-C(3)	178.3(6)
C(1)-C(2)-C(3)-C(4)	-1.9(10)
C(2)-C(3)-C(4)-C(5)	0.8(10)
C(1)-N(1)-C(5)-C(4)	-2.6(10)
Dy(1)-N(1)-C(5)-C(4)	175.3(5)
C(1)-N(1)-C(5)-C(6)	-177.5(6)
Dy(1)-N(1)-C(5)-C(6)	0.4(7)
C(3)-C(4)-C(5)-N(1)	1.5(10)
C(3)-C(4)-C(5)-C(6)	175.6(6)
Dy(1)-O(1)-C(6)-N(2)	132.2(5)
Dy(1)-O(1)-C(6)-C(5)	-47.1(6)
C(9)-N(2)-C(6)-O(1)	-167.4(6)
C(7)-N(2)-C(6)-O(1)	4.5(9)
C(9)-N(2)-C(6)-C(5)	11.9(9)
C(7)-N(2)-C(6)-C(5)	-176.3(6)
N(1)-C(5)-C(6)-O(1)	30.7(8)
C(4)-C(5)-C(6)-O(1)	-144.0(6)
N(1)-C(5)-C(6)-N(2)	-148.6(6)
C(4)-C(5)-C(6)-N(2)	36.7(10)
C(6)-N(2)-C(7)-C(8)	-92.3(8)
C(9)-N(2)-C(7)-C(8)	79.9(8)
C(13)-N(3)-C(9)-C(10)	-3.8(10)
C(13)-N(3)-C(9)-N(2)	179.8(6)
C(6)-N(2)-C(9)-N(3)	-123.7(6)
C(7)-N(2)-C(9)-N(3)	64.3(8)
C(6)-N(2)-C(9)-C(10)	59.8(9)
C(7)-N(2)-C(9)-C(10)	-112.3(7)
N(3)-C(9)-C(10)-C(11)	4.5(11)
N(2)-C(9)-C(10)-C(11)	-179.3(6)
C(9)-C(10)-C(11)-C(12)	-1.9(11)
C(10)-C(11)-C(12)-C(13)	-1.0(11)
C(9)-N(3)-C(13)-C(12)	0.6(10)
C(9)-N(3)-C(13)-C(14)	-178.8(6)
C(11)-C(12)-C(13)-N(3)	1.7(11)
C(11)-C(12)-C(13)-C(14)	-178.9(7)
C(19)-N(4)-C(15)-C(16)	0.5(10)
Dy(1)-N(4)-C(15)-C(16)	-155.3(5)
C(19)-N(4)-C(15)-C(1)	178.0(6)
Dy(1)-N(4)-C(15)-C(1)	22.2(7)
N(1)-C(1)-C(15)-N(4)	-18.3(9)
C(2)-C(1)-C(15)-N(4)	164.0(7)
N(1)-C(1)-C(15)-C(16)	159.2(7)
C(2)-C(1)-C(15)-C(16)	-18.5(11)
N(4)-C(15)-C(16)-C(17)	2.7(10)
C(1)-C(15)-C(16)-C(17)	-174.6(6)
C(15)-C(16)-C(17)-C(18)	-3.0(10)
C(16)-C(17)-C(18)-C(19)	0.2(10)
C(15)-N(4)-C(19)-C(18)	-3.5(10)
Dy(1)-N(4)-C(19)-C(18)	152.9(5)

C(15)-N(4)-C(19)-C(20)-179.2(6)
Dy(1)-N(4)-C(19)-C(20)-22.8(7)
C(17)-C(18)-C(19)-N(4)3.1(11)
C(17)-C(18)-C(19)-C(20)178.1(7)
Dy(1)-O(2)-C(20)-N(5) 171.7(5)
Dy(1)-O(2)-C(20)-C(19)-11.9(9)
C(23)-N(5)-C(20)-O(2)-164.3(7)
C(21)-N(5)-C(20)-O(2) 0.8(10)
C(23)-N(5)-C(20)-C(19)19.4(11)
C(21)-N(5)-C(20)-C(19)-175.5(6)
N(4)-C(19)-C(20)-O(2) 22.9(9)
C(18)-C(19)-C(20)-O(2)-152.6(7)
N(4)-C(19)-C(20)-N(5)-160.7(6)
C(18)-C(19)-C(20)-N(5)23.8(11)
C(20)-N(5)-C(21)-C(22)-73.4(9)
C(23)-N(5)-C(21)-C(22)92.5(8)
C(27)-N(6)-C(23)-C(24)0.4(12)
C(27)-N(6)-C(23)-N(5)-177.4(6)
C(20)-N(5)-C(23)-N(6) 51.7(10)
C(21)-N(5)-C(23)-N(6)-113.3(7)
C(20)-N(5)-C(23)-C(24)-126.2(8)
C(21)-N(5)-C(23)-C(24)68.7(9)
N(6)-C(23)-C(24)-C(25)0.1(12)
N(5)-C(23)-C(24)-C(25)177.9(7)
C(23)-C(24)-C(25)-C(26)-0.6(12)
C(24)-C(25)-C(26)-C(27)0.7(12)
C(23)-N(6)-C(27)-C(26)-0.4(11)
C(23)-N(6)-C(27)-C(28)178.2(8)
C(25)-C(26)-C(27)-N(6)-0.2(12)
C(25)-C(26)-C(27)-C(28)-178.7(8)
Dy(1)-O(1N)-N(1N)-O(3N)176.3(6)
Dy(1)-O(1N)-N(1N)-O(2N)-4.2(5)
Dy(1)-O(2N)-N(1N)-O(3N)-176.3(6)
Dy(1)-O(2N)-N(1N)-O(1N)4.2(5)
Dy(1)-O(4N)-N(2N)-O(6N)-177.2(6)
Dy(1)-O(4N)-N(2N)-O(5N)3.3(6)
Dy(1)-O(5N)-N(2N)-O(6N)177.3(6)
Dy(1)-O(5N)-N(2N)-O(4N)-3.3(6)
Dy(1)-O(8N)-N(3N)-O(9N)-179.1(5)
Dy(1)-O(8N)-N(3N)-O(7N)2.2(6)
Dy(1)-O(7N)-N(3N)-O(9N)179.2(6)
Dy(1)-O(7N)-N(3N)-O(8N)-2.1(6)

Structure parameters for Dy(3c)$(NO_3)_3$ – 2nd types of crystals

The large peak of residual electron density with the magnitude of more than 3 $eAng^{-3}$ was located near the N5 atom. This peak was considered as the solvent water molecule since a possible O-H...N hydrogen bond could be presupposed given that the distance between the N5 atom and this peak is approximately 3 Ang. Unfortunately, the modeling of this peak by the oxygen atom of a solvent water molecule led to an unreasonably large atomic displacement parameters, while corresponding disorder modeling was unsuccessful. The SQUEEZE procedure was performed, at that the electrons' count within the solvent accessible voids was 10 that is in a good agreement with the number of electrons in a water molecule. The data in cif-file (Formula, molecular weight, density etc.) are calculated for the model with the solvent water molecule.

Table S15 Atomic coordinates (x 10^4) and equivalent isotropic displacement parameters (Å^{2}x 10^3) for Dy(3c)$(NO_3)_3$ – 2nd types of crystals. U(eq) is defined as one third of the trace of the orthogonalized U^{ij} tensor.

	x	y	z	U(eq)
Dy(1)	7394(1)	5142(1)	4303(1)	15(1)
O(1)	6806(1)	7271(3)	4472(2)	18(1)
O(2)	7719(1)	2688(3)	3712(2)	13(1)
N(1)	6187(1)	8504(4)	4996(2)	17(1)
N(2)	8463(1)	1357(4)	4018(2)	14(1)
N(5)	5600(1)	7014(4)	5601(2)	22(1)
N(15)	6976(1)	5040(4)	5652(2)	13(1)
N(20)	9019(1)	-806(4)	4494(2)	18(1)
N(30)	7606(1)	2724(4)	5292(2)	12(1)
C(1)	6563(2)	7425(5)	5054(3)	16(1)
C(2)	6088(2)	9567(5)	4234(3)	22(1)
C(3)	5831(2)	8601(6)	3492(3)	29(1)

C(4)	5818(2)	8474(5)	5539(3)	18(1)
C(6)	5261(2)	6921(6)	6125(3)	26(1)
C(6A)	5035(2)	5231(7)	6209(3)	47(2)
C(7)	5142(2)	8280(6)	6564(3)	30(1)
C(8)	5373(2)	9774(6)	6487(3)	31(1)
C(9)	5726(2)	9902(6)	5961(3)	25(1)
C(10)	6722(2)	6391(5)	5825(2)	14(1)
C(11)	6660(2)	6809(5)	6627(3)	20(1)
C(12)	6846(2)	5716(5)	7265(3)	20(1)
C(13)	7086(2)	4288(5)	7091(2)	16(1)
C(14)	7150(2)	3987(5)	6262(2)	13(1)
C(16)	8030(2)	1879(4)	4209(2)	12(1)
C(17)	8576(2)	1648(5)	3168(3)	18(1)
C(18)	8865(2)	3235(6)	3119(3)	29(1)
C(19)	8864(2)	729(5)	4636(2)	16(1)
C(21)	9390(2)	-1445(5)	5062(3)	20(1)
C(21A)	9556(2)	-3183(5)	4894(3)	26(1)
C(22)	9600(2)	-563(5)	5764(3)	22(1)
C(23)	9440(2)	1043(6)	5878(3)	22(1)
C(24)	9067(2)	1725(5)	5298(3)	20(1)
C(25)	7893(2)	1522(5)	5052(2)	12(1)
C(26)	8009(2)	72(5)	5508(2)	14(1)
C(27)	7828(2)	-113(5)	6248(2)	19(1)
C(28)	7547(2)	1144(5)	6514(3)	19(1)
C(29)	7441(2)	2550(5)	6024(2)	13(1)
O(1N)	7891(1)	6285(3)	5584(2)	18(1)
O(2N)	8312(1)	5090(3)	4736(2)	17(1)
O(3N)	8692(1)	5974(5)	5935(2)	46(1)
O(4N)	6756(1)	4824(4)	2992(2)	26(1)
O(5N)	6628(1)	3377(4)	4055(2)	22(1)
O(6N)	6119(1)	3118(5)	2883(2)	41(1)
O(7N)	7772(1)	5927(3)	3086(2)	24(1)
O(8N)	7681(1)	7967(3)	3901(2)	17(1)
O(9N)	8026(1)	8387(4)	2801(2)	22(1)
N(1N)	8310(1)	5798(4)	5435(2)	17(1)
N(2N)	6486(1)	3764(5)	3298(2)	24(1)
N(3N)	7837(1)	7466(4)	3248(2)	16(1)
N(1S)	9726(2)	5069(6)	7208(3)	48(1)
C(1S)	9507(2)	6194(6)	7372(3)	26(1)
C(2S)	9222(2)	7596(6)	7588(3)	27(1)

Table S16 Bond lengths [Å] and angles [°] for Dy(3c)(NO_3)$_3$ – 2nd types of crystals.

Dy(1)-O(1)	2.387(3)	Dy(1)-N(1N)	2.890(4)
Dy(1)-O(2)	2.424(3)	Dy(1)-N(3N)	2.928(3)
Dy(1)-O(7N)	2.460(3)	O(1)-C(1)	1.247(5)
Dy(1)-O(1N)	2.468(3)	O(2)-C(16)	1.252(5)
Dy(1)-O(2N)	2.475(3)	N(1)-C(1)	1.332(5)
Dy(1)-O(5N)	2.496(3)	N(1)-C(4)	1.440(5)
Dy(1)-O(8N)	2.521(3)	N(1)-C(2)	1.494(5)
Dy(1)-N(30)	2.529(3)	N(2)-C(16)	1.331(5)
Dy(1)-O(4N)	2.531(3)	N(2)-C(19)	1.446(5)
Dy(1)-N(15)	2.636(3)	N(2)-C(17)	1.486(5)

N(5)-C(4)	1.325(5)	C(27)-C(28)	1.379(6)
N(5)-C(6)	1.356(5)	C(27)-H(27A)	0.9500
N(15)-C(14)	1.332(5)	C(28)-C(29)	1.387(5)
N(15)-C(10)	1.340(5)	C(28)-H(28A)	0.9500
N(20)-C(19)	1.336(5)	O(1N)-N(1N)	1.266(4)
N(20)-C(21)	1.352(5)	O(2N)-N(1N)	1.273(4)
N(30)-C(25)	1.339(5)	O(3N)-N(1N)	1.216(5)
N(30)-C(29)	1.349(5)	O(4N)-N(2N)	1.276(5)
C(1)-C(10)	1.509(5)	O(5N)-N(2N)	1.270(4)
C(2)-C(3)	1.508(6)	O(6N)-N(2N)	1.224(5)
C(2)-H(2A)	0.9900	O(7N)-N(3N)	1.271(4)
C(2)-H(2B)	0.9900	O(8N)-N(3N)	1.275(4)
C(3)-H(3A)	0.9800	O(9N)-N(3N)	1.211(4)
C(3)-H(3B)	0.9800	N(1S)-C(1S)	1.138(6)
C(3)-H(3C)	0.9800	C(1S)-C(2S)	1.442(7)
C(4)-C(9)	1.382(6)	C(2S)-H(2SC)	0.9800
C(6)-C(7)	1.373(7)	C(2S)-H(2SB)	0.9800
C(6)-C(6A)	1.506(6)	C(2S)-H(2SA)	0.9800
C(6A)-H(6AA)	0.9800		
C(6A)-H(6AB)	0.9800	O(1)-Dy(1)-O(2)	157.36(10)
C(6A)-H(6AC)	0.9800	O(1)-Dy(1)-O(7N)	106.51(10)
C(7)-C(8)	1.370(7)	O(2)-Dy(1)-O(7N)	70.20(9)
C(7)-H(7A)	0.9500	O(1)-Dy(1)-O(1N)	85.31(10)
C(8)-C(9)	1.390(6)	O(2)-Dy(1)-O(1N)	117.11(9)
C(8)-H(8A)	0.9500	O(7N)-Dy(1)-O(1N)	110.16(10)
C(9)-H(9A)	0.9500	O(1)-Dy(1)-O(2N)	129.82(9)
C(10)-C(11)	1.387(5)	O(2)-Dy(1)-O(2N)	71.63(9)
C(11)-C(12)	1.390(6)	O(7N)-Dy(1)-O(2N)	72.59(10)
C(11)-H(11A)	0.9500	O(1N)-Dy(1)-O(2N)	51.80(9)
C(12)-C(13)	1.372(6)	O(1)-Dy(1)-O(5N)	82.56(10)
C(12)-H(12A)	0.9500	O(2)-Dy(1)-O(5N)	79.69(9)
C(13)-C(14)	1.410(5)	O(7N)-Dy(1)-O(5N)	117.51(10)
C(13)-H(13A)	0.9500	O(1N)-Dy(1)-O(5N)	132.31(9)
C(14)-C(29)	1.486(5)	O(2N)-Dy(1)-O(5N)	144.07(9)
C(16)-C(25)	1.510(5)	O(1)-Dy(1)-O(8N)	67.98(9)
C(17)-C(18)	1.506(6)	O(2)-Dy(1)-O(8N)	118.80(9)
C(17)-H(17A)	0.9900	O(7N)-Dy(1)-O(8N)	51.12(9)
C(17)-H(17B)	0.9900	O(1N)-Dy(1)-O(8N)	74.67(9)
C(18)-H(18A)	0.9800	O(2N)-Dy(1)-O(8N)	75.54(9)
C(18)-H(18B)	0.9800	O(5N)-Dy(1)-O(8N)	138.98(9)
C(18)-H(18C)	0.9800	O(1)-Dy(1)-N(30)	124.30(10)
C(19)-C(24)	1.382(6)	O(2)-Dy(1)-N(30)	64.45(9)
C(21)-C(22)	1.386(6)	O(7N)-Dy(1)-N(30)	129.03(10)
C(21)-C(21A)	1.506(6)	O(1N)-Dy(1)-N(30)	73.28(9)
C(21A)-H(21A)	0.9800	O(2N)-Dy(1)-N(30)	72.14(10)
C(21A)-H(21B)	0.9800	O(5N)-Dy(1)-N(30)	76.23(10)
C(21A)-H(21C)	0.9800	O(8N)-Dy(1)-N(30)	144.10(10)
C(22)-C(23)	1.385(6)	O(1)-Dy(1)-O(4N)	77.99(10)
C(22)-H(22A)	0.9500	O(2)-Dy(1)-O(4N)	79.93(9)
C(23)-C(24)	1.380(6)	O(7N)-Dy(1)-O(4N)	70.32(10)
C(23)-H(23A)	0.9500	O(1N)-Dy(1)-O(4N)	162.49(9)
C(24)-H(24A)	0.9500	O(2N)-Dy(1)-O(4N)	139.04(9)
C(25)-C(26)	1.390(5)	O(5N)-Dy(1)-O(4N)	50.93(9)
C(26)-C(27)	1.382(5)	O(8N)-Dy(1)-O(4N)	93.96(9)
C(26)-H(26A)	0.9500	N(30)-Dy(1)-O(4N)	120.78(10)

O(1)-Dy(1)-N(15)	62.49(9)	C(2)-C(3)-H(3A)	109.5
O(2)-Dy(1)-N(15)	122.76(9)	C(2)-C(3)-H(3B)	109.5
O(7N)-Dy(1)-N(15)	166.91(10)	H(3A)-C(3)-H(3B)	109.5
O(1N)-Dy(1)-N(15)	63.66(10)	C(2)-C(3)-H(3C)	109.5
O(2N)-Dy(1)-N(15)	108.26(9)	H(3A)-C(3)-H(3C)	109.5
O(5N)-Dy(1)-N(15)	69.84(10)	H(3B)-C(3)-H(3C)	109.5
O(8N)-Dy(1)-N(15)	115.98(9)	N(5)-C(4)-C(9)	125.3(4)
N(30)-Dy(1)-N(15)	61.89(10)	N(5)-C(4)-N(1)	115.2(4)
O(4N)-Dy(1)-N(15)	111.83(10)	C(9)-C(4)-N(1)	119.5(4)
O(1)-Dy(1)-N(1N)	108.52(10)	N(5)-C(6)-C(7)	121.8(4)
O(2)-Dy(1)-N(1N)	94.08(9)	N(5)-C(6)-C(6A)	115.8(4)
O(7N)-Dy(1)-N(1N)	92.22(10)	C(7)-C(6)-C(6A)	122.4(4)
O(1N)-Dy(1)-N(1N)	25.83(9)	C(6)-C(6A)-H(6AA)	109.5
O(2N)-Dy(1)-N(1N)	26.01(9)	C(6)-C(6A)-H(6AB)	109.5
O(5N)-Dy(1)-N(1N)	144.48(9)	H(6AA)-C(6A)-H(6AB)	109.5
O(8N)-Dy(1)-N(1N)	74.43(9)	C(6)-C(6A)-H(6AC)	109.5
N(30)-Dy(1)-N(1N)	69.68(10)	H(6AA)-C(6A)-H(6AC)	109.5
O(4N)-Dy(1)-N(1N)	162.54(10)	H(6AB)-C(6A)-H(6AC)	109.5
N(15)-Dy(1)-N(1N)	85.25(10)	C(8)-C(7)-C(6)	119.9(4)
O(1)-Dy(1)-N(3N)	87.56(9)	C(8)-C(7)-H(7A)	120.1
O(2)-Dy(1)-N(3N)	94.26(9)	C(6)-C(7)-H(7A)	120.1
O(7N)-Dy(1)-N(3N)	25.44(9)	C(7)-C(8)-C(9)	119.6(4)
O(1N)-Dy(1)-N(3N)	92.27(9)	C(7)-C(8)-H(8A)	120.2
O(2N)-Dy(1)-N(3N)	71.75(9)	C(9)-C(8)-H(8A)	120.2
O(5N)-Dy(1)-N(3N)	132.80(9)	C(4)-C(9)-C(8)	116.4(4)
O(8N)-Dy(1)-N(3N)	25.70(8)	C(4)-C(9)-H(9A)	121.8
N(30)-Dy(1)-N(3N)	142.40(10)	C(8)-C(9)-H(9A)	121.8
O(4N)-Dy(1)-N(3N)	81.87(10)	N(15)-C(10)-C(11)	122.8(4)
N(15)-Dy(1)-N(3N)	141.67(9)	N(15)-C(10)-C(1)	110.9(3)
N(1N)-Dy(1)-N(3N)	82.24(9)	C(11)-C(10)-C(1)	126.1(4)
C(1)-O(1)-Dy(1)	127.3(2)	C(10)-C(11)-C(12)	117.8(4)
C(16)-O(2)-Dy(1)	114.6(2)	C(10)-C(11)-H(11A)	121.1
C(1)-N(1)-C(4)	123.2(3)	C(12)-C(11)-H(11A)	121.1
C(1)-N(1)-C(2)	117.5(3)	C(13)-C(12)-C(11)	120.0(4)
C(4)-N(1)-C(2)	118.2(3)	C(13)-C(12)-H(12A)	120.0
C(16)-N(2)-C(19)	122.6(3)	C(11)-C(12)-H(12A)	120.0
C(16)-N(2)-C(17)	119.5(3)	C(12)-C(13)-C(14)	118.6(4)
C(19)-N(2)-C(17)	117.3(3)	C(12)-C(13)-H(13A)	120.7
C(4)-N(5)-C(6)	117.0(4)	C(14)-C(13)-H(13A)	120.7
C(14)-N(15)-C(10)	119.3(3)	N(15)-C(14)-C(13)	121.4(4)
C(14)-N(15)-Dy(1)	119.6(2)	N(15)-C(14)-C(29)	116.0(3)
C(10)-N(15)-Dy(1)	117.2(2)	C(13)-C(14)-C(29)	122.5(4)
C(19)-N(20)-C(21)	117.0(4)	O(2)-C(16)-N(2)	122.8(4)
C(25)-N(30)-C(29)	118.5(3)	O(2)-C(16)-C(25)	116.4(3)
C(25)-N(30)-Dy(1)	116.8(2)	N(2)-C(16)-C(25)	120.8(3)
C(29)-N(30)-Dy(1)	124.7(3)	N(2)-C(17)-C(18)	111.8(3)
O(1)-C(1)-N(1)	120.4(4)	N(2)-C(17)-H(17A)	109.3
O(1)-C(1)-C(10)	117.7(4)	C(18)-C(17)-H(17A)	109.3
N(1)-C(1)-C(10)	121.9(4)	N(2)-C(17)-H(17B)	109.3
N(1)-C(2)-C(3)	111.6(4)	C(18)-C(17)-H(17B)	109.3
N(1)-C(2)-H(2A)	109.3	H(17A)-C(17)-H(17B)	107.9
C(3)-C(2)-H(2A)	109.3	C(17)-C(18)-H(18A)	109.5
N(1)-C(2)-H(2B)	109.3	C(17)-C(18)-H(18B)	109.5
C(3)-C(2)-H(2B)	109.3	H(18A)-C(18)-H(18B)	109.5
H(2A)-C(2)-H(2B)	108.0	C(17)-C(18)-H(18C)	109.5

H(18A)-C(18)-H(18C)	109.5
H(18B)-C(18)-H(18C)	109.5
N(20)-C(19)-C(24)	125.0(4)
N(20)-C(19)-N(2)	115.1(4)
C(24)-C(19)-N(2)	119.9(4)
N(20)-C(21)-C(22)	121.9(4)
N(20)-C(21)-C(21A)	116.1(4)
C(22)-C(21)-C(21A)	122.0(4)
C(21)-C(21A)-H(21A)	109.5
C(21)-C(21A)-H(21B)	109.5
H(21A)-C(21A)-H(21B)	109.5
C(21)-C(21A)-H(21C)	109.5
H(21A)-C(21A)-H(21C)	109.5
H(21B)-C(21A)-H(21C)	109.5
C(21)-C(22)-C(23)	119.5(4)
C(21)-C(22)-H(22A)	120.2
C(23)-C(22)-H(22A)	120.2
C(24)-C(23)-C(22)	119.3(4)
C(24)-C(23)-H(23A)	120.4
C(22)-C(23)-H(23A)	120.4
C(23)-C(24)-C(19)	117.3(4)
C(23)-C(24)-H(24A)	121.4
C(19)-C(24)-H(24A)	121.4
N(30)-C(25)-C(26)	122.7(3)
N(30)-C(25)-C(16)	111.8(3)
C(26)-C(25)-C(16)	125.3(3)
C(27)-C(26)-C(25)	118.4(4)
C(27)-C(26)-H(26A)	120.8
C(25)-C(26)-H(26A)	120.8
C(28)-C(27)-C(26)	119.3(4)
C(28)-C(27)-H(27A)	120.3
C(26)-C(27)-H(27A)	120.3
C(27)-C(28)-C(29)	119.3(4)
C(27)-C(28)-H(28A)	120.4
C(29)-C(28)-H(28A)	120.4
N(30)-C(29)-C(28)	121.8(4)
N(30)-C(29)-C(14)	114.7(3)
C(28)-C(29)-C(14)	123.5(4)
N(1N)-O(1N)-Dy(1)	96.0(2)
N(1N)-O(2N)-Dy(1)	95.5(2)
N(2N)-O(4N)-Dy(1)	95.5(2)
N(2N)-O(5N)-Dy(1)	97.3(2)
N(3N)-O(7N)-Dy(1)	98.3(2)
N(3N)-O(8N)-Dy(1)	95.3(2)
O(3N)-N(1N)-O(1N)	121.9(4)
O(3N)-N(1N)-O(2N)	121.6(4)
O(1N)-N(1N)-O(2N)	116.5(3)
O(3N)-N(1N)-Dy(1)	175.7(3)
O(1N)-N(1N)-Dy(1)	58.12(19)
O(2N)-N(1N)-Dy(1)	58.48(19)
O(6N)-N(2N)-O(5N)	121.3(4)
O(6N)-N(2N)-O(4N)	122.4(4)
O(5N)-N(2N)-O(4N)	116.2(4)
O(6N)-N(2N)-Dy(1)	177.0(3)
O(5N)-N(2N)-Dy(1)	57.3(2)
O(4N)-N(2N)-Dy(1)	59.0(2)
O(9N)-N(3N)-O(7N)	122.0(3)
O(9N)-N(3N)-O(8N)	122.8(3)
O(7N)-N(3N)-O(8N)	115.2(3)
O(9N)-N(3N)-Dy(1)	178.1(3)
O(7N)-N(3N)-Dy(1)	56.22(19)
O(8N)-N(3N)-Dy(1)	59.03(18)
N(1S)-C(1S)-C(2S)	178.8(6)
C(1S)-C(2S)-H(2SC)	109.5
C(1S)-C(2S)-H(2SB)	109.5
H(2SC)-C(2S)-H(2SB)	109.5
C(1S)-C(2S)-H(2SA)	109.5
H(2SC)-C(2S)-H(2SA)	109.5
H(2SB)-C(2S)-H(2SA)	109.5

Table S17 Anisotropic displacement parameters (Å^{2}x 10^3) for Dy(3c)$(NO_3)_3$ – 2nd types of crystals. The anisotropic displacement factor exponent takes the form: $-2\pi^2$[h^2 $a^{*2}U^{11}$ + ... + 2 h k a* b* U^{12}]

	U^{11}	U^{22}	U^{33}	U^{23}	U^{13}	U^{12}
Dy(1)	21(1)	13(1)	12(1)	1(1)	5(1)	3(1)
O(1)	24(2)	17(1)	15(2)	6(1)	11(1)	7(1)
O(2)	17(2)	12(1)	10(1)	1(1)	2(1)	3(1)
N(1)	22(2)	15(2)	18(2)	3(1)	9(2)	3(2)
N(2)	19(2)	13(2)	10(2)	0(1)	5(1)	2(1)
N(5)	23(2)	26(2)	17(2)	-4(2)	4(2)	-2(2)
N(15)	17(2)	14(2)	10(2)	-1(1)	5(1)	2(1)
N(20)	20(2)	16(2)	18(2)	2(1)	6(2)	2(2)
N(30)	15(2)	13(2)	9(2)	1(1)	2(1)	1(1)
C(1)	17(2)	11(2)	20(2)	1(2)	4(2)	0(2)
C(2)	28(3)	15(2)	25(2)	7(2)	7(2)	10(2)
C(3)	24(3)	35(3)	26(3)	5(2)	0(2)	7(2)

C(4)	17(2)	21(2)	16(2)	3(2)	2(2)	6(2)
C(6)	27(3)	38(3)	15(2)	-3(2)	4(2)	-10(2)
C(6A)	62(4)	51(4)	33(3)	-9(3)	23(3)	-30(3)
C(7)	20(3)	49(3)	22(2)	-2(2)	9(2)	4(2)
C(8)	27(3)	41(3)	26(2)	-9(2)	7(2)	13(2)
C(9)	23(2)	23(2)	29(2)	-3(2)	7(2)	3(2)
C(10)	14(2)	14(2)	13(2)	2(2)	4(2)	0(2)
C(11)	18(2)	20(2)	23(2)	-5(2)	8(2)	3(2)
C(12)	19(2)	25(2)	16(2)	-2(2)	6(2)	1(2)
C(13)	18(2)	20(2)	10(2)	2(2)	4(2)	1(2)
C(14)	12(2)	16(2)	12(2)	2(2)	2(2)	-2(2)
C(16)	19(2)	7(2)	12(2)	-2(2)	5(2)	0(2)
C(17)	25(2)	18(2)	14(2)	1(2)	10(2)	3(2)
C(18)	38(3)	27(2)	26(3)	3(2)	17(2)	-3(2)
C(19)	17(2)	16(2)	15(2)	4(2)	7(2)	3(2)
C(21)	20(2)	21(2)	22(2)	8(2)	11(2)	5(2)
C(21A)	24(3)	23(2)	31(3)	7(2)	4(2)	10(2)
C(22)	18(2)	26(2)	19(2)	6(2)	2(2)	3(2)
C(23)	16(2)	30(2)	19(2)	-1(2)	3(2)	-3(2)
C(24)	19(2)	18(2)	22(2)	0(2)	5(2)	2(2)
C(25)	16(2)	13(2)	8(2)	-3(2)	2(2)	-2(2)
C(26)	21(2)	9(2)	14(2)	-2(2)	5(2)	2(2)
C(27)	33(2)	11(2)	15(2)	6(2)	6(2)	7(2)
C(28)	29(3)	18(2)	11(2)	3(2)	8(2)	3(2)
C(29)	16(2)	14(2)	11(2)	-2(2)	4(2)	0(2)
O(1N)	24(2)	15(1)	15(2)	0(1)	5(1)	0(1)
O(2N)	21(2)	16(1)	15(1)	-4(1)	5(1)	0(1)
O(3N)	25(2)	81(3)	31(2)	-22(2)	-3(2)	-8(2)
O(4N)	31(2)	30(2)	15(2)	4(1)	2(1)	1(2)
O(5N)	25(2)	26(2)	15(2)	4(1)	0(1)	-2(1)
O(6N)	26(2)	62(3)	31(2)	-4(2)	-8(2)	-10(2)
O(7N)	46(2)	15(2)	14(2)	-2(1)	12(1)	-9(1)
O(8N)	26(2)	12(1)	15(2)	-2(1)	8(1)	2(1)
O(9N)	30(2)	20(2)	18(2)	4(1)	10(1)	-6(1)
N(1N)	20(2)	16(2)	15(2)	-2(1)	3(2)	-7(2)
N(2N)	21(2)	28(2)	23(2)	-1(2)	3(2)	4(2)
N(3N)	20(2)	15(2)	12(2)	1(1)	2(2)	1(1)
N(1S)	37(3)	59(3)	43(3)	2(2)	-5(2)	17(3)
C(1S)	21(3)	34(3)	20(2)	5(2)	-3(2)	-1(2)
C(2S)	32(3)	28(2)	23(2)	1(2)	8(2)	-9(2)

Table S18 Hydrogen coordinates (x 10^4) and isotropic displacement parameters ($Å^2$x 10^3) for Dy(3c)$(NO_3)_3$ – 2nd types of crystals

	x	y	z	U(eq)
H(2A)	6408	10014	4112	27
H(2B)	5875	10520	4338	27
H(3A)	5764	9341	3008	43
H(3B)	5515	8149	3613	43
H(3C)	6047	7686	3372	43
H(6AA)	5301	4429	6404	71
H(6AB)	4854	4875	5667	71

H(6AC)	4803	5289	6610	71
H(7A)	4900	8185	6921	36
H(8A)	5293	10717	6791	37
H(9A)	5895	10917	5895	30
H(11A)	6495	7808	6737	24
H(12A)	6807	5958	7821	23
H(13A)	7207	3519	7520	19
H(17A)	8259	1706	2769	22
H(17B)	8772	700	3005	22
H(18A)	8934	3380	2552	43
H(18B)	9181	3177	3509	43
H(18C)	8668	4180	3265	43
H(21A)	9266	-3844	4647	39
H(21B)	9713	-3698	5417	39
H(21C)	9797	-3138	4508	39
H(22A)	9852	-1057	6164	26
H(23A)	9585	1669	6351	26
H(24A)	8954	2832	5351	24
H(26A)	8207	-771	5316	17
H(27A)	7897	-1096	6570	23
H(28A)	7426	1047	7028	22
H(2SC)	9055	7297	8057	41
H(2SB)	8971	7904	7109	41
H(2SA)	9447	8539	7745	41

Table S19. Torsion angles [°] for Dy(3c)(NO_3)$_3$ – 2nd types of crystals.

Dy(1)-O(1)-C(1)-N(1)	-168.4(3)	N(1)-C(1)-C(10)-N(15)	158.7(4)
Dy(1)-O(1)-C(1)-C(10)	13.4(5)	O(1)-C(1)-C(10)-C(11)	151.6(4)
C(4)-N(1)-C(1)-O(1)	164.6(4)	N(1)-C(1)-C(10)-C(11)	-26.6(6)
C(2)-N(1)-C(1)-O(1)	-3.1(6)	N(15)-C(10)-C(11)-C(12)	-3.8(6)
C(4)-N(1)-C(1)-C(10)	-17.3(6)	C(1)-C(10)-C(11)-C(12)	-177.9(4)
C(2)-N(1)-C(1)-C(10)	175.0(4)	C(10)-C(11)-C(12)-C(13)	0.6(6)
C(1)-N(1)-C(2)-C(3)	75.9(5)	C(11)-C(12)-C(13)-C(14)	1.7(6)
C(4)-N(1)-C(2)-C(3)	-92.5(4)	C(10)-N(15)-C(14)-C(13)	-2.0(6)
C(6)-N(5)-C(4)-C(9)	-0.5(7)	Dy(1)-N(15)-C(14)-C(13)	155.1(3)
C(6)-N(5)-C(4)-N(1)	178.1(4)	C(10)-N(15)-C(14)-C(29)	-178.1(3)
C(1)-N(1)-C(4)-N(5)	-51.9(6)	Dy(1)-N(15)-C(14)-C(29)	-21.0(4)
C(2)-N(1)-C(4)-N(5)	115.7(4)	C(12)-C(13)-C(14)-N(15)	-1.0(6)
C(1)-N(1)-C(4)-C(9)	126.8(4)	C(12)-C(13)-C(14)-C(29)	174.8(4)
C(2)-N(1)-C(4)-C(9)	-65.6(5)	Dy(1)-O(2)-C(16)-N(2)	-132.5(3)
C(4)-N(5)-C(6)-C(7)	1.1(7)	Dy(1)-O(2)-C(16)-C(25)	47.2(4)
C(4)-N(5)-C(6)-C(6A)	-177.6(4)	C(19)-N(2)-C(16)-O(2)	167.0(4)
N(5)-C(6)-C(7)-C(8)	-1.0(7)	C(17)-N(2)-C(16)-O(2)	-3.6(6)
C(6A)-C(6)-C(7)-C(8)	177.6(5)	C(19)-N(2)-C(16)-C(25)	-12.6(5)
C(6)-C(7)-C(8)-C(9)	0.2(7)	C(17)-N(2)-C(16)-C(25)	176.7(3)
N(5)-C(4)-C(9)-C(8)	-0.2(7)	C(16)-N(2)-C(17)-C(18)	93.5(5)
N(1)-C(4)-C(9)-C(8)	-178.8(4)	C(19)-N(2)-C(17)-C(18)	-77.6(4)
C(7)-C(8)-C(9)-C(4)	0.4(7)	C(21)-N(20)-C(19)-C(24)	2.7(6)
C(14)-N(15)-C(10)-C(11)	4.5(6)	C(21)-N(20)-C(19)-N(2)	-179.4(4)
Dy(1)-N(15)-C(10)-C(11)	-153.1(3)	C(16)-N(2)-C(19)-N(20)	123.3(4)
C(14)-N(15)-C(10)-C(1)	179.4(3)	C(17)-N(2)-C(19)-N(20)	-65.8(5)
Dy(1)-N(15)-C(10)-C(1)	21.8(4)	C(16)-N(2)-C(19)-C(24)	-58.7(5)
O(1)-C(1)-C(10)-N(15)	-23.2(5)	C(17)-N(2)-C(19)-C(24)	112.2(4)

C(19)-N(20)-C(21)-C(22)	0.4(6)	Dy(1)-N(30)-C(29)-C(28)	176.1(3)
C(19)-N(20)-C(21)-C(21A)	179.4(4)	C(25)-N(30)-C(29)-C(14)	176.6(3)
N(20)-C(21)-C(22)-C(23)	-2.3(6)	Dy(1)-N(30)-C(29)-C(14)	-5.0(5)
C(21A)-C(21)-C(22)-C(23)	178.7(4)	C(27)-C(28)-C(29)-N(30)	0.1(6)
C(21)-C(22)-C(23)-C(24)	1.2(6)	C(27)-C(28)-C(29)-C(14)	-178.7(4)
C(22)-C(23)-C(24)-C(19)	1.6(6)	N(15)-C(14)-C(29)-N(30)	17.1(5)
N(20)-C(19)-C(24)-C(23)	-3.7(6)	C(13)-C(14)-C(29)-N(30)	-159.0(4)
N(2)-C(19)-C(24)-C(23)	178.5(4)	N(15)-C(14)-C(29)-C(28)	-164.1(4)
C(29)-N(30)-C(25)-C(26)	3.0(6)	C(13)-C(14)-C(29)-C(28)	19.8(6)
Dy(1)-N(30)-C(25)-C(26)	-175.5(3)	Dy(1)-O(1N)-N(1N)-O(3N)	-174.9(4)
C(29)-N(30)-C(25)-C(16)	178.1(3)	Dy(1)-O(1N)-N(1N)-O(2N)	4.1(3)
Dy(1)-N(30)-C(25)-C(16)	-0.4(4)	Dy(1)-O(2N)-N(1N)-O(3N)	175.0(4)
O(2)-C(16)-C(25)-N(30)	-30.7(5)	Dy(1)-O(2N)-N(1N)-O(1N)	-4.1(3)
N(2)-C(16)-C(25)-N(30)	149.0(4)	Dy(1)-O(5N)-N(2N)-O(6N)	176.9(4)
O(2)-C(16)-C(25)-C(26)	144.3(4)	Dy(1)-O(5N)-N(2N)-O(4N)	-1.4(4)
N(2)-C(16)-C(25)-C(26)	-36.1(6)	Dy(1)-O(4N)-N(2N)-O(6N)	-176.9(4)
N(30)-C(25)-C(26)-C(27)	-1.4(6)	Dy(1)-O(4N)-N(2N)-O(5N)	1.4(4)
C(16)-C(25)-C(26)-C(27)	-175.9(4)	Dy(1)-O(7N)-N(3N)-O(9N)	179.2(3)
C(25)-C(26)-C(27)-C(28)	-0.8(6)	Dy(1)-O(7N)-N(3N)-O(8N)	-2.1(4)
C(26)-C(27)-C(28)-C(29)	1.5(6)	Dy(1)-O(8N)-N(3N)-O(9N)	-179.3(3)
C(25)-N(30)-C(29)-C(28)	-2.3(6)	Dy(1)-O(8N)-N(3N)-O(7N)	2.0(3)

UV-vis Titration Data in CH_3CN

2c ligand with trivalent lanthanide ions

2c with $La(NO_3)_3 \cdot 6H_2O$

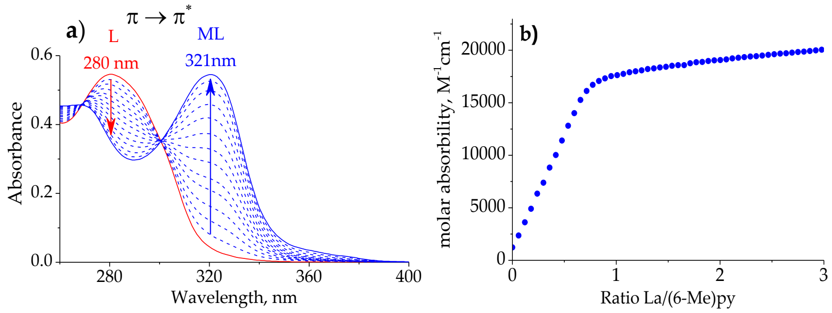

Spectrophotometric titration in “dry” CH_3CN solution: 1 μl of 3.91 mM titrant solution $La(NO_3)_3 \cdot 6H_2O$ was added to 2 ml of $3.26 \cdot 10^{-2}$ mM 2c: a) spectrophotometric data, where red line – 2c spectrum, blue dashed lines – aliquots of $La(NO_3)_3 \cdot 6H_2O$ and blue line – complex $[La2c](NO_3)_3$; b) titration curve at 321 nm.

2c with $Ce(NO_3)_3 \cdot 6H_2O$

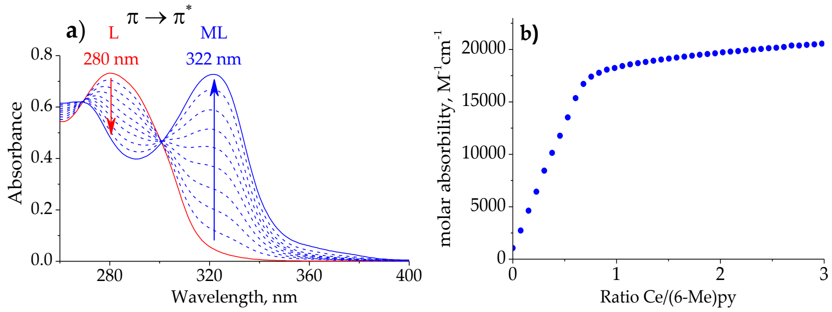

Spectrophotometric titration in "dry" CH_3CN solution: 1 µl of 6.60 mM titrant solution $Ce(NO_3)_3{\cdot}6H_2O$ was added to 2 ml of $4.35{\cdot}10^{-2}$ mM 2c: a) spectrophotometric data, where red line – 2c spectrum, blue dashed lines – aliquots of $Ce(NO_3)_3{\cdot}6H_2O$ and blue line – complex $[Ce2c](NO_3)_3$; b) titration curve at 322 nm.

2c with $Pr(NO_3)_3{\cdot}6H_2O$

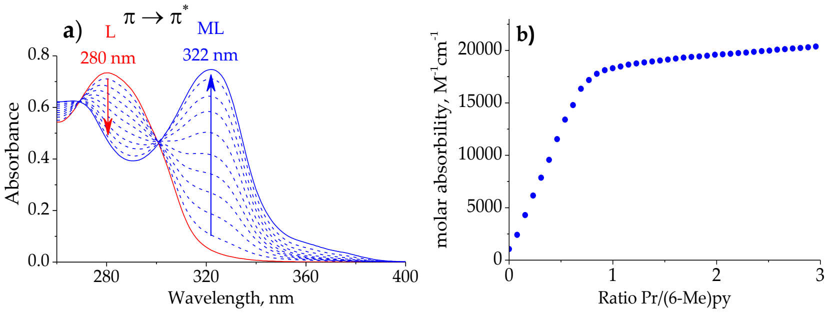

Spectrophotometric titration in "dry" CH_3CN solution: 1 µl of 6.72 mM titrant solution $Pr(NO_3)_3{\cdot}6H_2O$ was added to 2 ml of $4.35{\cdot}10^{-2}$ mM 2c: a) spectrophotometric data, where red line – 2c spectrum, blue dashed lines – aliquots of $Pr(NO_3)_3{\cdot}6H_2O$ and blue line – complex $[Pr2c](NO_3)_3$; b) titration curve at 322 nm.

2c with $Nd(NO_3)_3{\cdot}6H_2O$

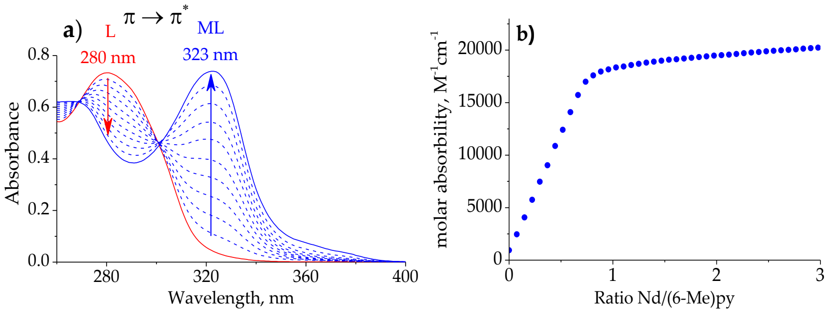

Spectrophotometric titration in "dry" CH_3CN solution: 1 µl of 6.45 mM titrant solution $Nd(NO_3)_3{\cdot}6H_2O$ was added to 2 ml of $4.35{\cdot}10^{-2}$ mM 2c: a) spectrophotometric data, where red line – 2c spectrum, blue dashed lines – aliquots of $Nd(NO_3)_3{\cdot}6H_2O$ and blue line – complex $[Nd2c](NO_3)_3$; b) titration curve at 323 nm.

2c with $Sm(NO_3)_3{\cdot}6H_2O$

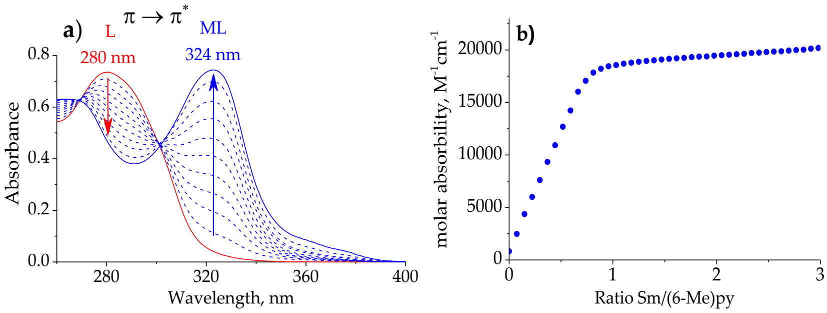

Spectrophotometric titration in "dry" CH_3CN solution: 1 µl of 6.45 mM titrant solution $Sm(NO_3)_3{\cdot}6H_2O$ was added to 2 ml of $4.35{\cdot}10^{-2}$ mM 2c: a) spectrophotometric data, where red line – 2c spectrum, blue dashed lines – aliquots of $Sm(NO_3)_3{\cdot}6H_2O$ and blue line – complex $[Sm2c](NO_3)_3$; b) titration curve at 324 nm.

2c with $Gd(NO_3)_3{\cdot}6H_2O$

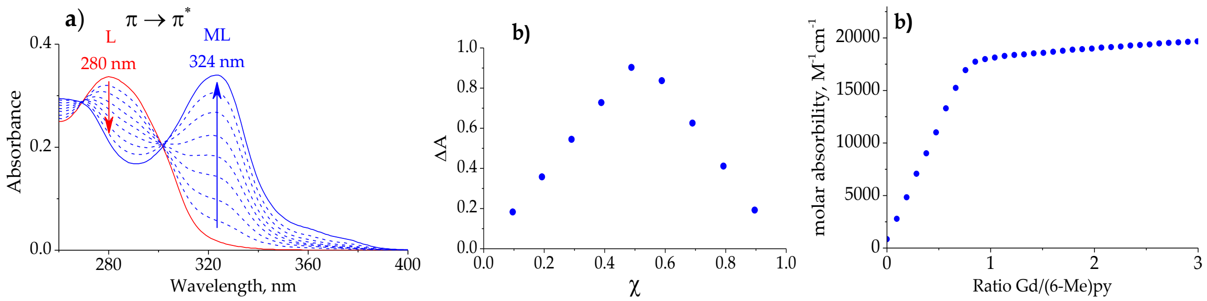

Spectrophotometric titration in "dry" CH_3CN solution: 1 µl of 3.82 mM titrant solution $Gd(NO_3)_3{\cdot}6H_2O$ was added to 2 ml of $2.01{\cdot}10^{-2}$ mM 2c: a) spectrophotometric data, where red line – 2c spectrum, blue dashed lines – aliquots of $Gd(NO_3)_3{\cdot}6H_2O$ and blue line – complex $[Gd2c](NO_3)_3$; b) the method of continuous variation for 0,106 mM 2c with 0,111 mM $Gd(NO_3)_3{\cdot}6H_2O$ at 324 nm; c) titration curve at 324 nm.

2c with $Er(NO_3)_3{\cdot}5H_2O$

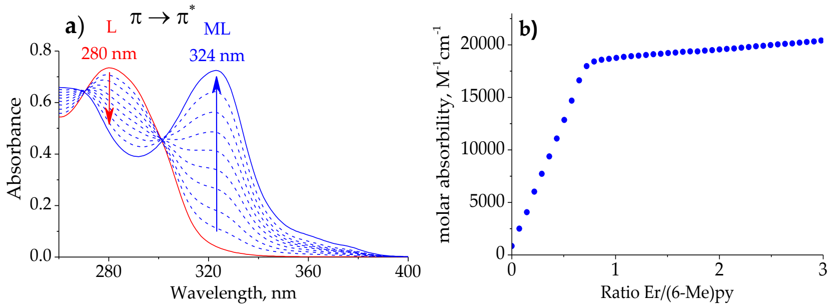

Spectrophotometric titration in "dry" CH_3CN solution: 1 µl of 6.30 mM titrant solution $Er(NO_3)_3{\cdot}6H_2O$ was added to 2 ml of $4.35{\cdot}10^{-2}$ mM 2c: a) spectrophotometric data, where red line – 2c spectrum, blue dashed lines – aliquots of $Er(NO_3)_3{\cdot}6H_2O$ and blue line – complex $[Er2c](NO_3)_3$; b) titration curve at 324 nm.

2c with $Tm(NO_3)_3{\cdot}5H_2O$

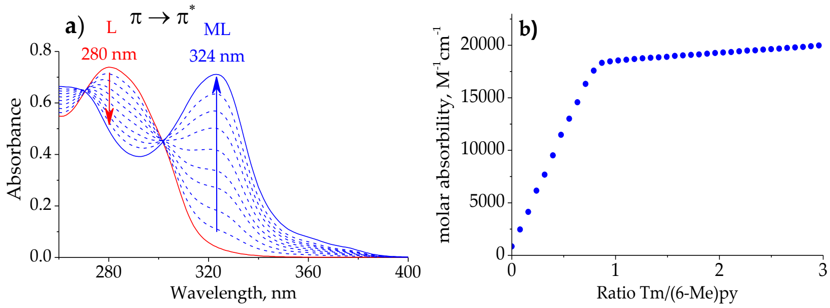

Spectrophotometric titration in "dry" CH_3CN solution: 1 µl of 6.90 mM titrant solution $Er(NO_3)_3{\cdot}6H_2O$ was added to 2 ml of $4.35{\cdot}10^{-2}$ mM 2c: a) spectrophotometric data, where red line – 2c spectrum, blue dashed lines – aliquots of $Er(NO_3)_3{\cdot}6H_2O$ and blue line – complex $[Er2c](NO_3)_3$; b) titration curve at 324 nm.

2c with $Yb(NO_3)_3{\cdot}5H_2O$

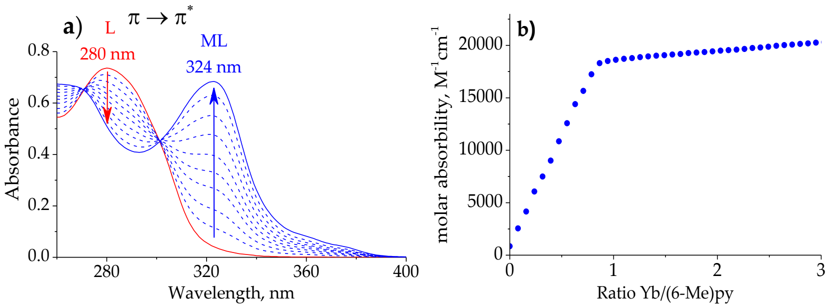

Spectrophotometric titration in "dry" CH_3CN solution: 1 µl of 6.88 mM titrant solution $Yb(NO_3)_3{\cdot}6H_2O$ was added to 2 ml of $4.35{\cdot}10^{-2}$ mM 2c: a) spectrophotometric data, where red line – 2c spectrum, blue dashed lines – aliquots of $Yb(NO_3)_3{\cdot}6H_2O$ and blue line – complex $[Yb2c](NO_3)_3$; b) titration curve at 324 nm.

Repeated check of selective result

2c with $Tm(NO_3)_3{\cdot}5H_2O$

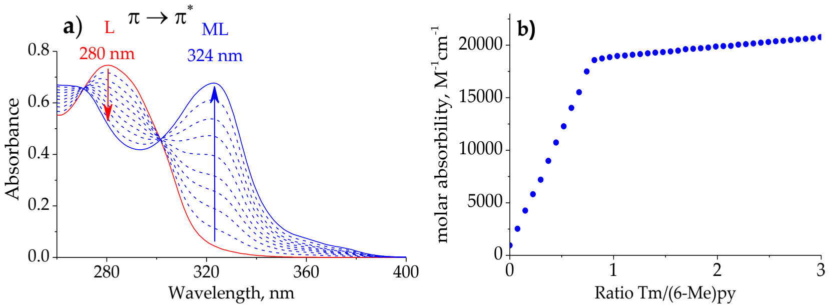

Spectrophotometric titration in "dry" CH_3CN solution: 1 µl of 6.90 mM titrant solution $Er(NO_3)_3{\cdot}6H_2O$ was added to 2 ml of $4.35{\cdot}10^{-2}$ mM 2c: a) spectrophotometric data, where red line – 2c spectrum, blue dashed lines – aliquots of $Er(NO_3)_3{\cdot}6H_2O$ and blue line – complex $[Er2c](NO_3)_3$; b) titration curve at 324 nm.

Table 20 *Log β_1 values for the stability of trivalent lanthanides ions with 2c ligand in acetonitrile*

Ln^{3+} / L	La	Ce	Pr	Nd	Sm	Gd	Er	Tm	Yb
**3c**	6.02 ±0.04	5.89 ±0.04	5.97 ±0.04	6.05 ±0.05	6.18 ±0.06	6.36 ±0.05	6.56 ±0.1	6.71 ±0.1	6.98 ±0.2

Table 21 Repeated-measured log β_1 values for the stability of lanthanides ions with 2c ligand acetonitrile

Ln^{3+} / L	Tm 1	Tm 2
**3c**	6.73 ±0.1	6.68 ±0.1

NMR of Ligands and their REE complexes

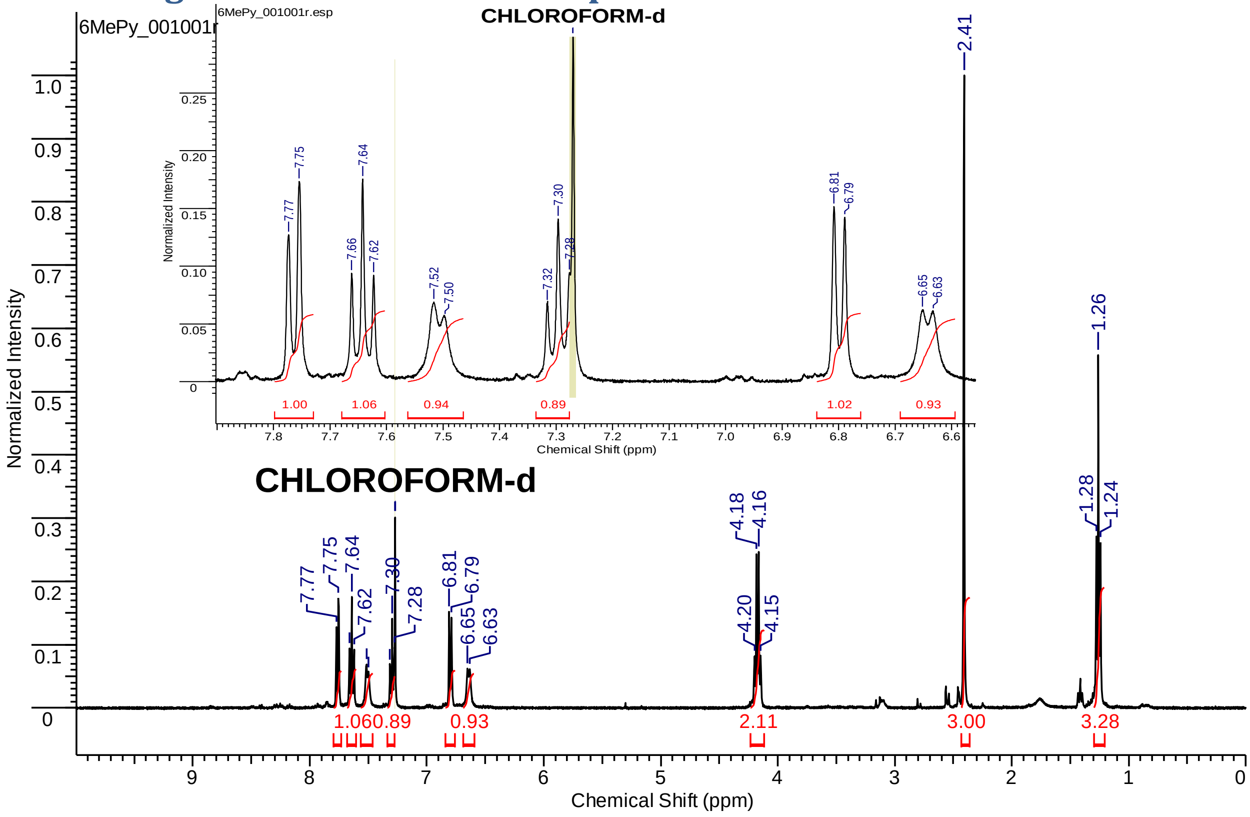

Fig S1 1H Spectrum of 6-Me-2-Py amide 2c in CDCl3

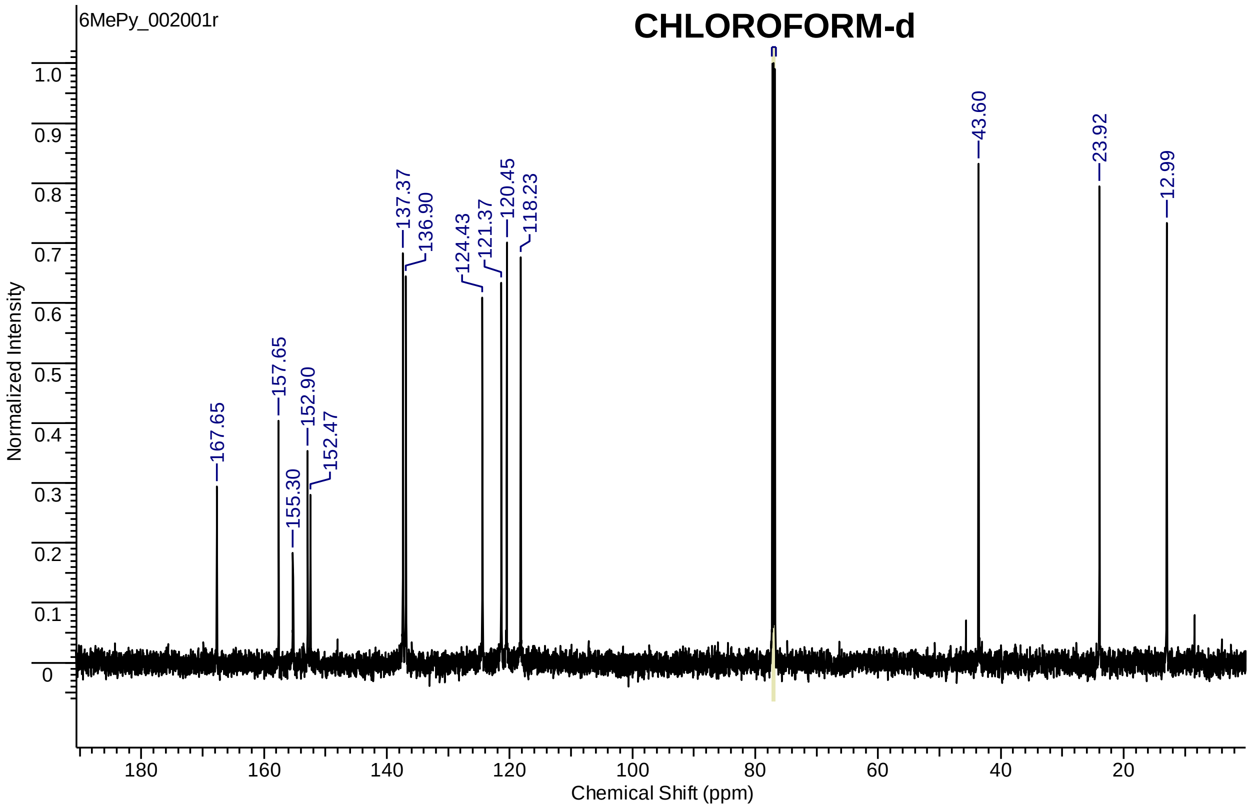

Fig S2 13C Spectrum of 6-Me-2-Py amide 2c in CDCl3

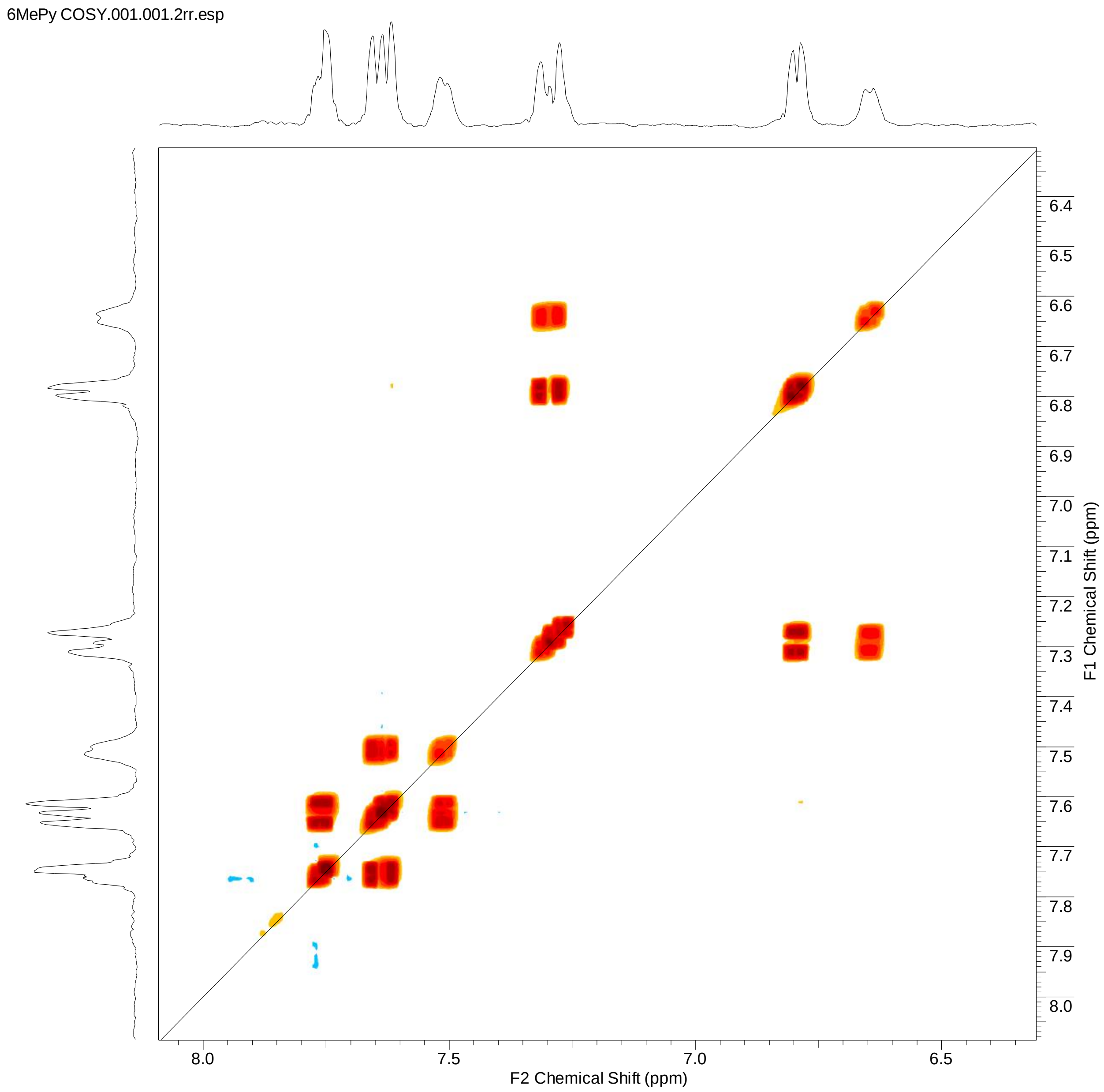

Fig S3 2D COSY Spectrum of 6-Me-2-Py ligand 2c in CDCl3

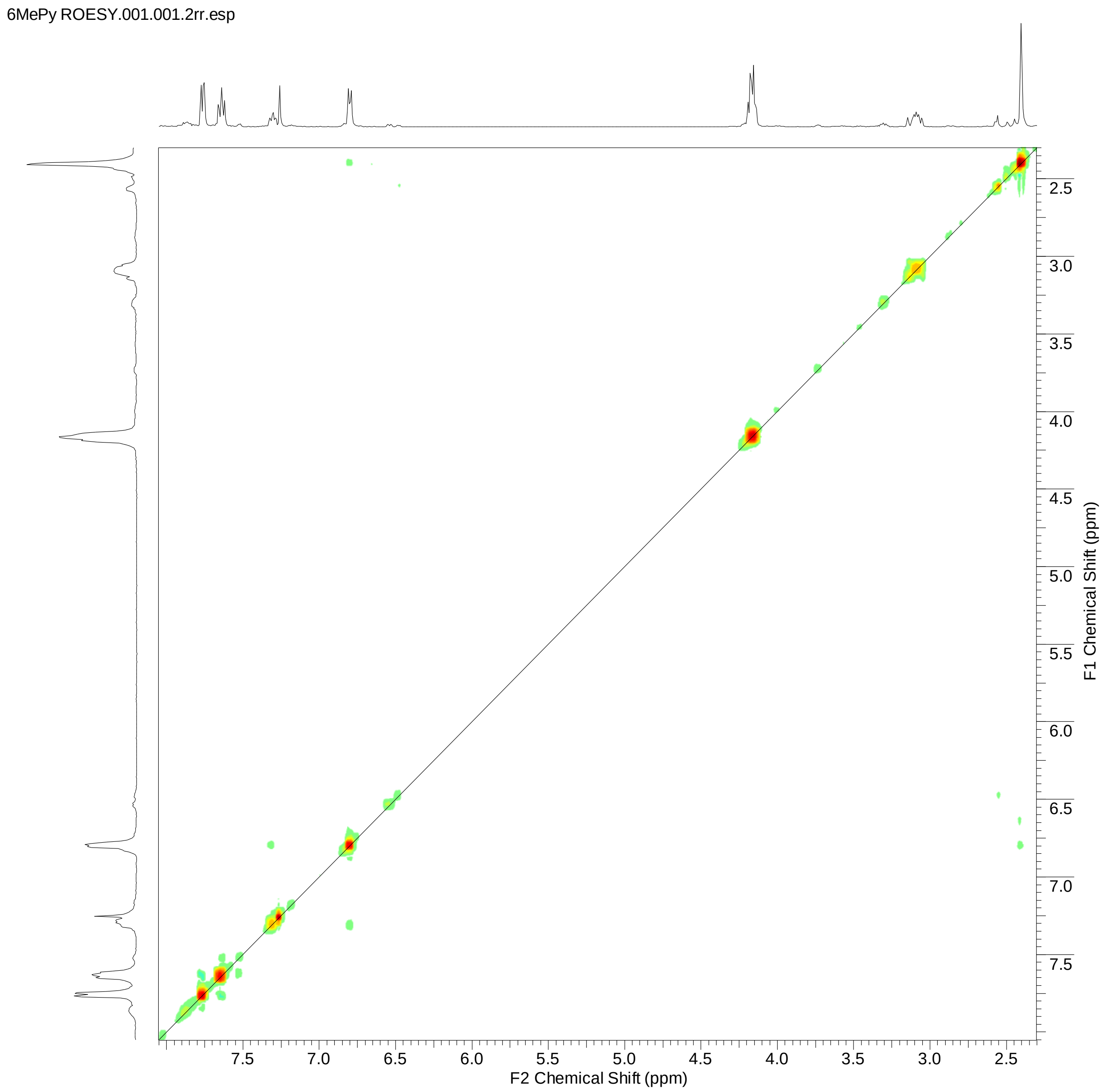

Fig S4 2D ROESY Spectrum of 6-Me-2-Py ligand 2c in CDCl3

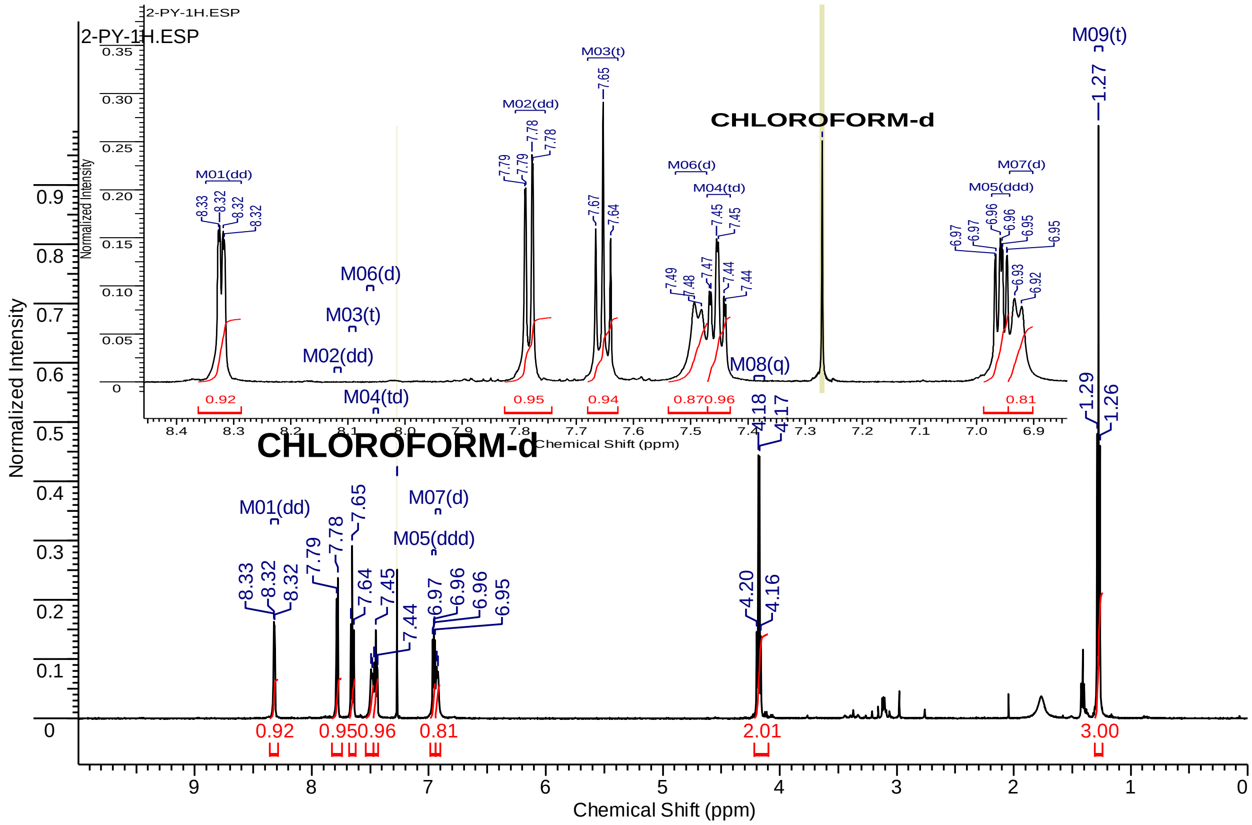

Fig S5 1H Spectrum of 2-Py ligand 2b in CDCl3

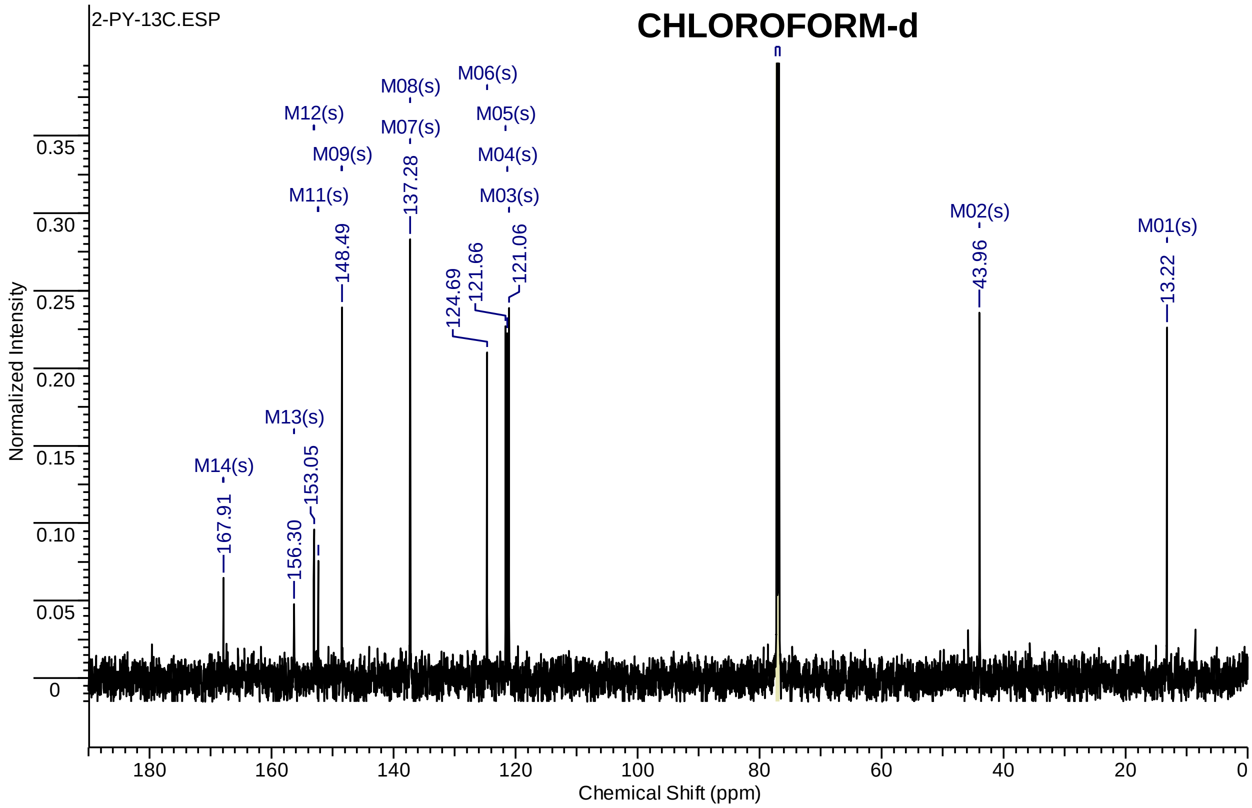

Fig S6 13C Spectrum of 2-Py ligand 2b in CDCl3

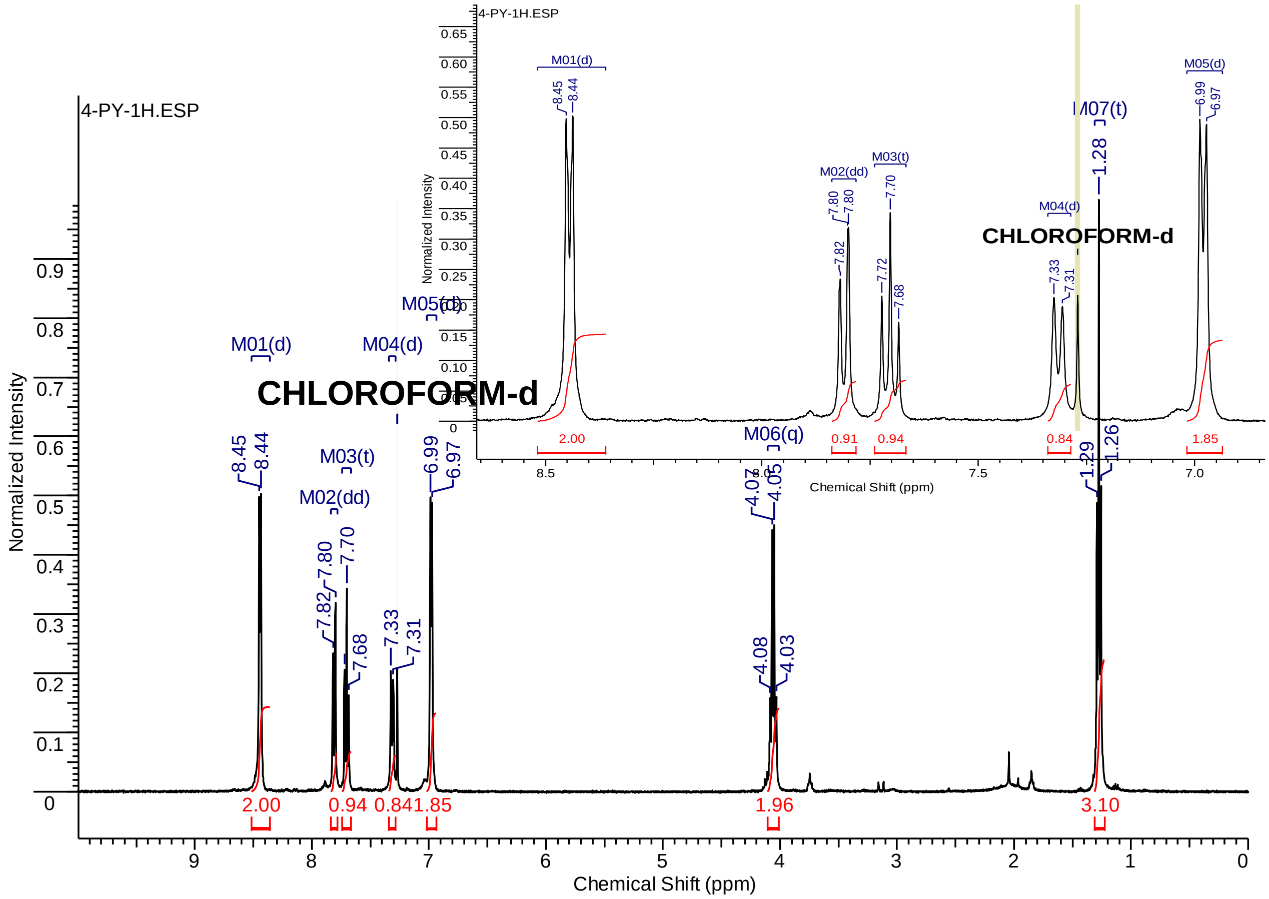

Fig 7 1H Spectrum of 4-Py ligand 2d in CDCl3

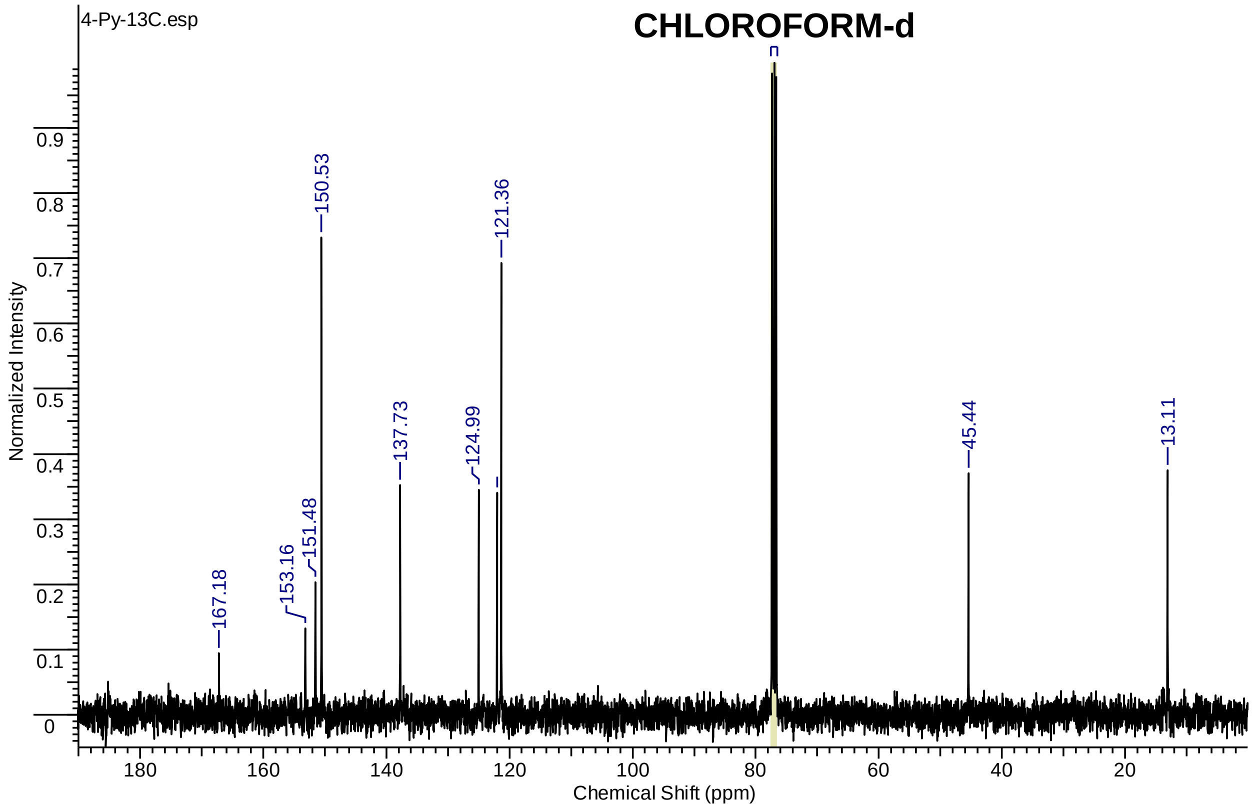

Fig S8 13C Spectrum of 4-Py ligand 2d in CDCl3

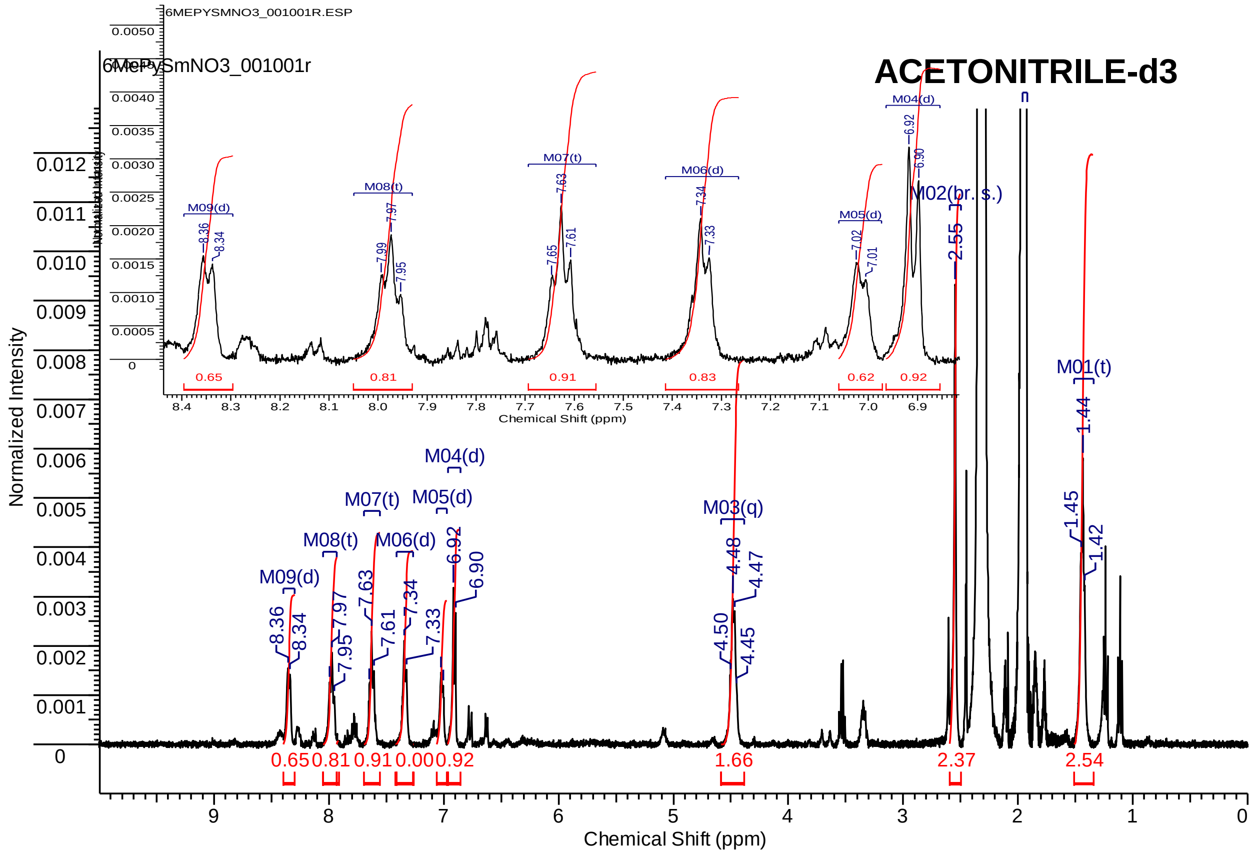

Fig S9 1H Spectrum of 3c-Sm in acetonitrile-d3

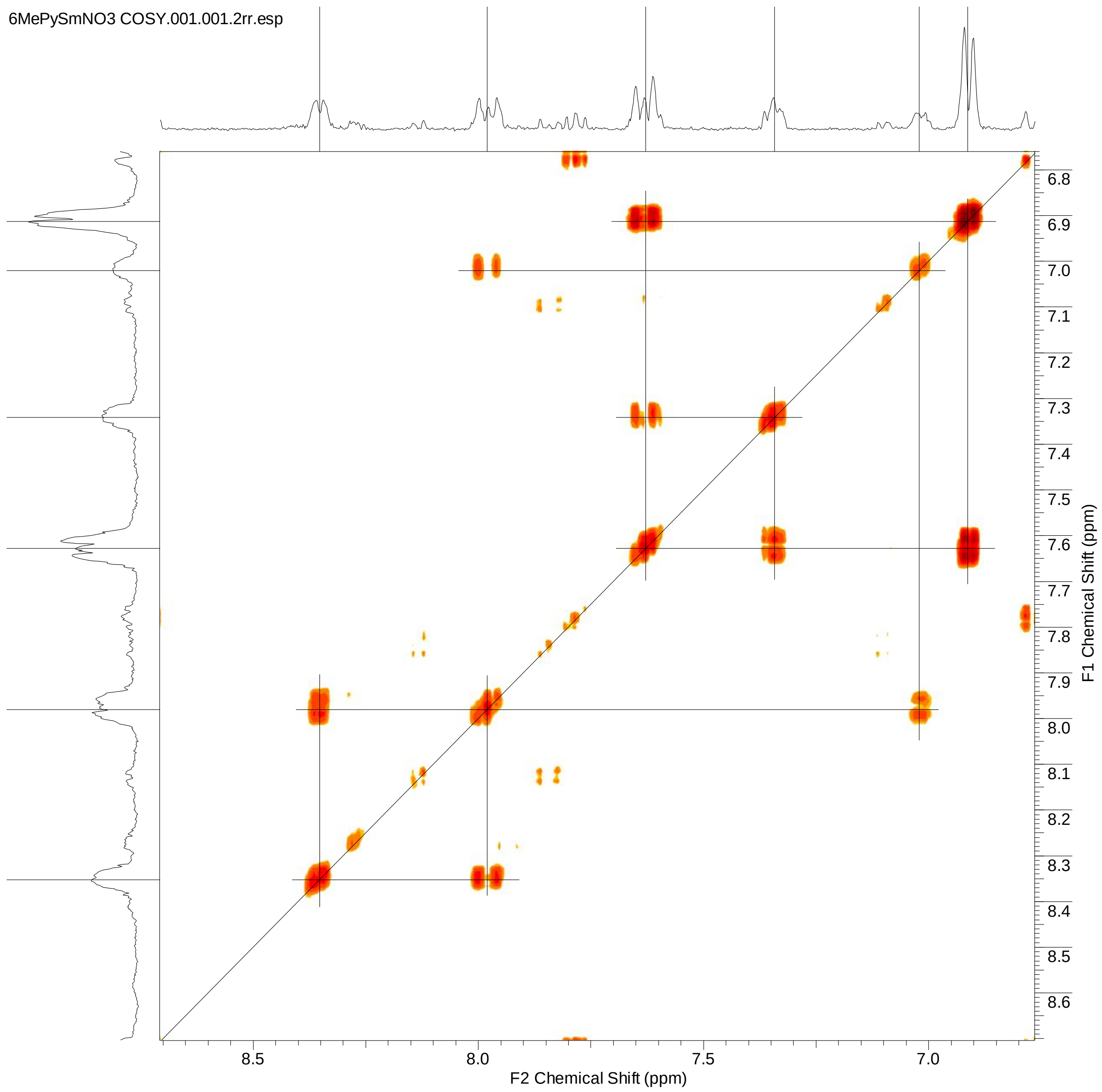

Fig S10 2D COSY Spectrum of 3c-Sm in acetonitrile-d3

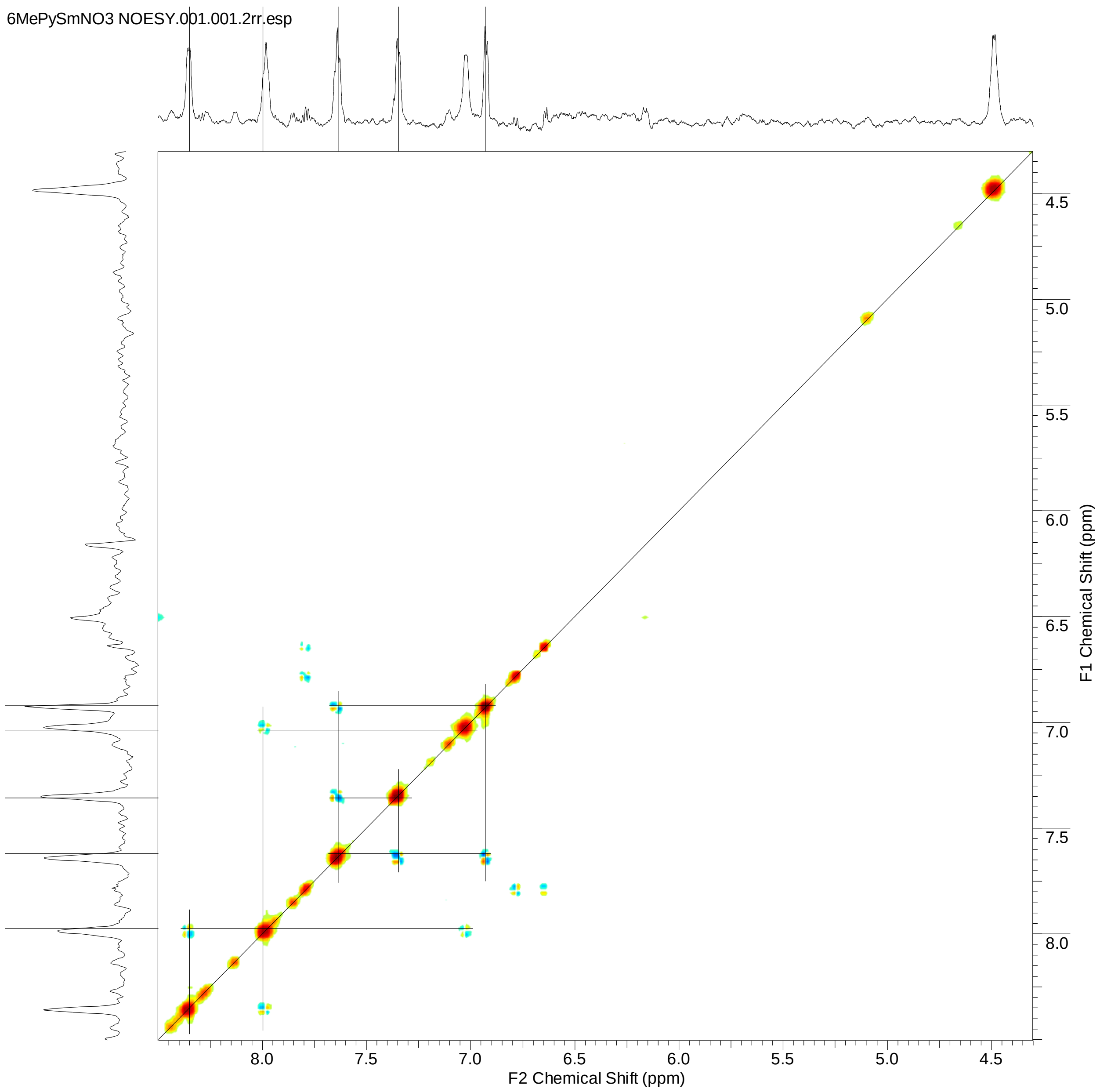

Fig S11 2D NESY Spectrum of 3c-Sm in acetonitrile-d3

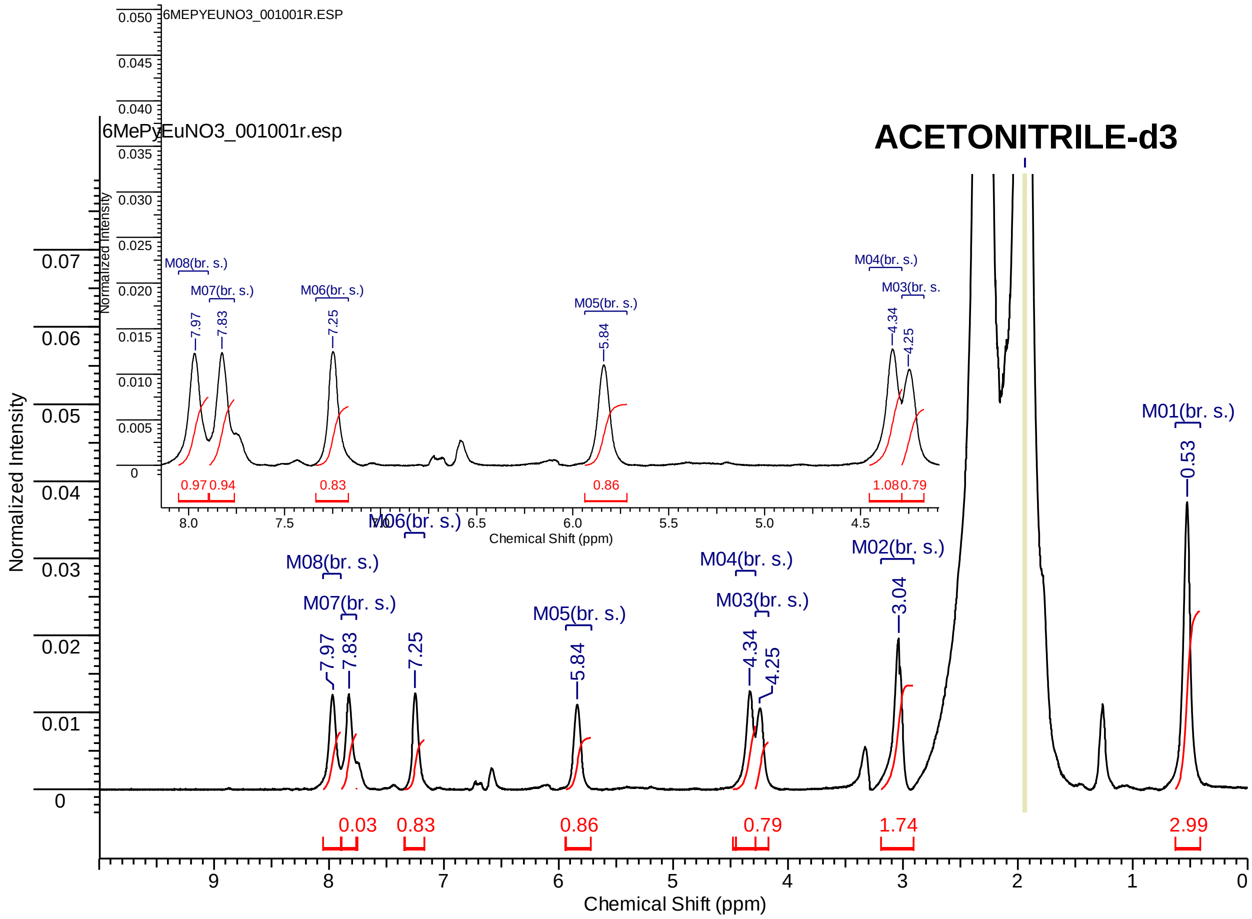

Fig 12 1H Spectrum of 3c-Eu in acetonitrile-d3

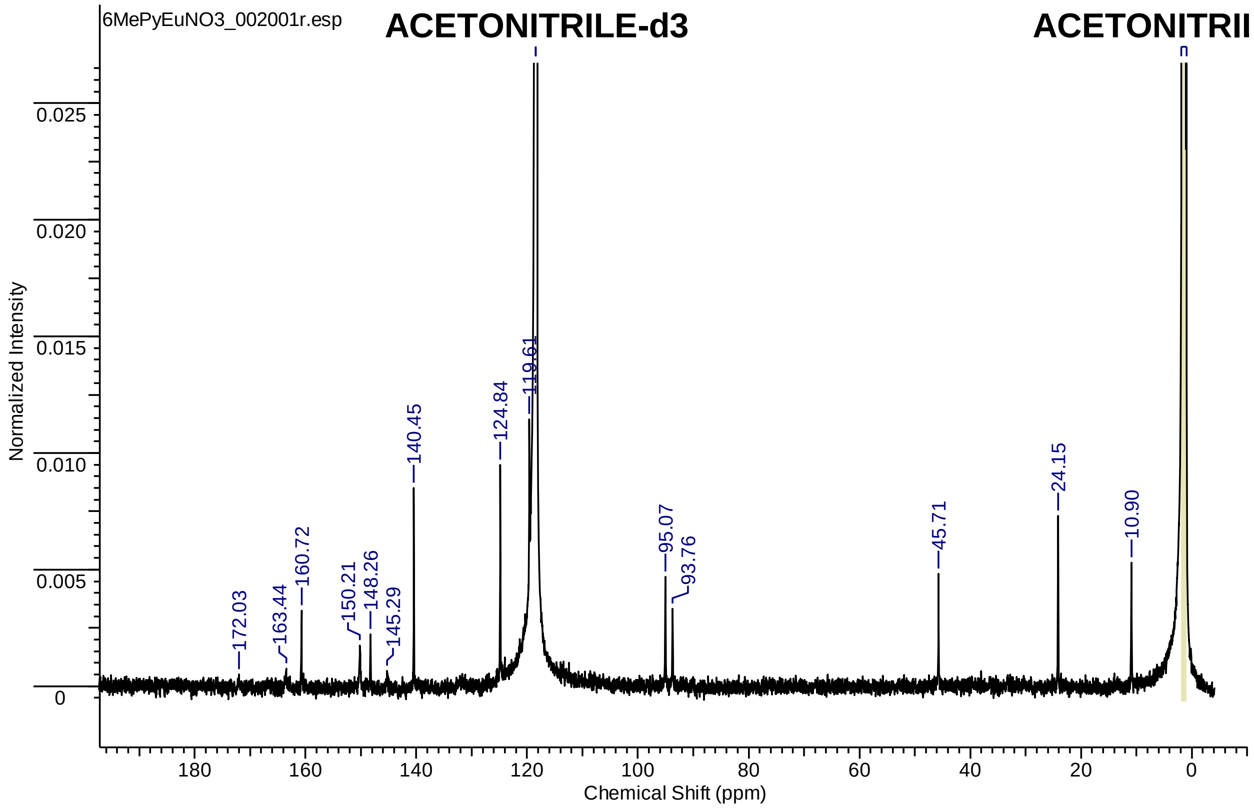

Fig S13 13C Spectrum of 3c-Eu in acetonitrile-d3

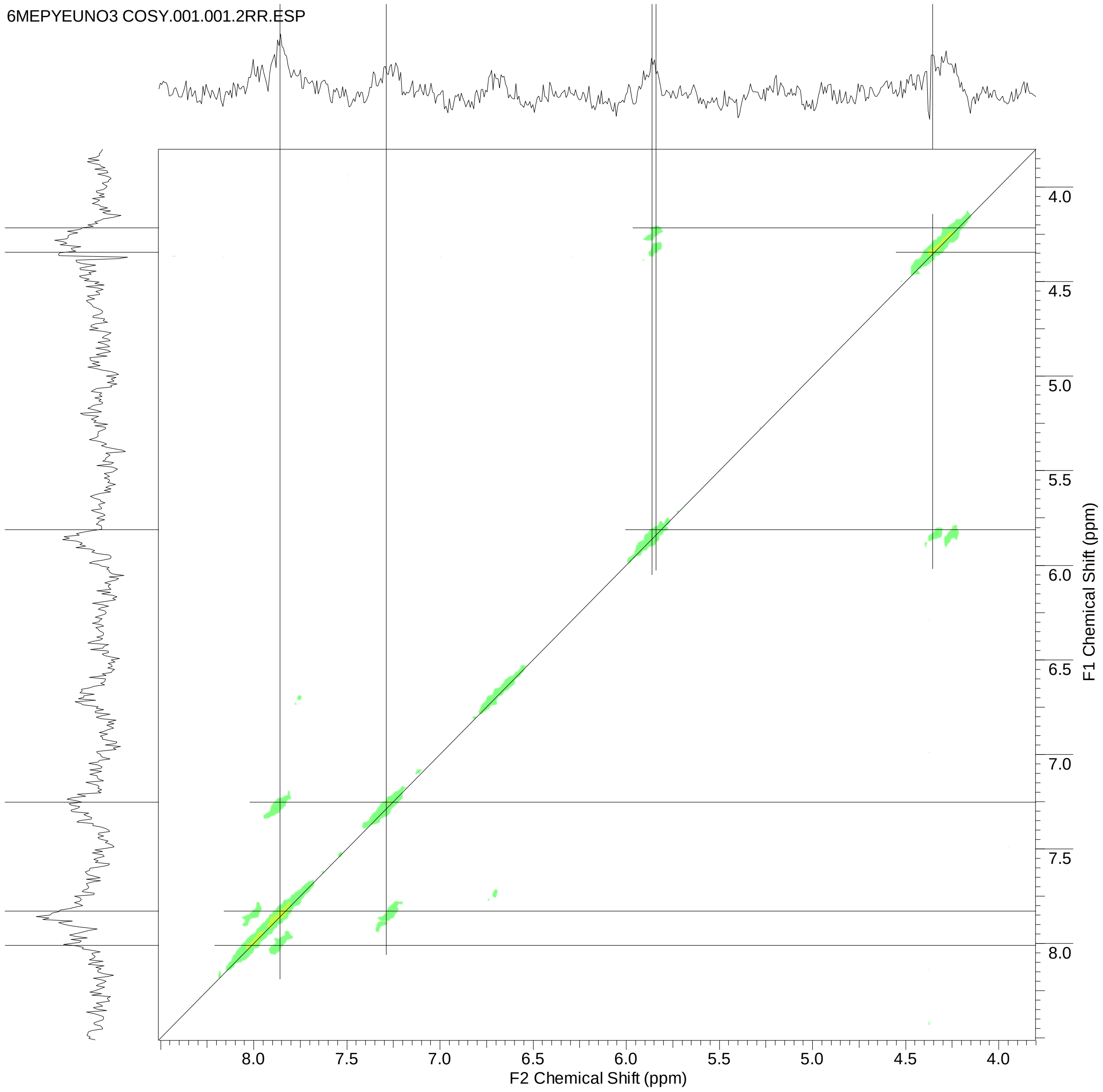

Fig S14 2D COSY Spectrum of 3c-Eu in acetonitrile-d3
